# ﻿The diatom genus *Ulnaria* (Bacillariophyta) in China

**DOI:** 10.3897/phytokeys.228.101080

**Published:** 2023-06-23

**Authors:** Bing Liu

**Affiliations:** 1 College of Biology and Environmental Sciences, Jishou University, Jishou 416000, China Jishou University Jishou China

**Keywords:** Girdle band structures, life history stages, striation patterns, *Ulnaria*, valvocopula

## Abstract

This study deals with *Ulnaria* species found and described from two regions of China with large climate differences during the period of 2014–2022. The first region, located in the Wuling Mountains and Hunan province, has a subtropical climate and the second in Qinghai, a northwest province of China, has a highland continental climate which is characterized by a cold and long winter and warm, short summer. Previously there were nine new *Ulnaria* species published from the first region. This study describes 14 additional new *Ulnaria* taxa, nine of which were found in the first region and five of which were found in the second region. A key to the *Ulnaria* species that have been described from China is provided. The main morphological characteristics for 63 *Ulnaria* taxa are summarized in Appendices which allow the division of these *Ulnaria* taxa into three groups: the seven members of group one all possess both uniseriate striae and valve marginal spines, the 42 members of group two all possess uniseriate or mostly uniseriate striae but without the valve marginal spines, and the 14 members of group three all possess mostly biseriate striae and without valve marginal spines. To summarize the morphological characters of the published *Ulnaria* taxa and 14 taxa described in this study several conclusions for the characterization of *Ulnaria* are drawn: 1) each cell has two valve-appressed, long plate-like plastids; 2) living cells of many *Ulnaria* species often lie in girdle view on a slide because they have deep mantles and some copulae associated with either the epivalve or the hypovalve so that the cell depth is often larger than the valve width; 3) the basic structures forming a valve include sternum, virgae, and vimines/viminules; 4) the valvocopula is a closed hoop which has a similar ultrastructure in all *Ulnaria* taxa but differs from the other copulae in structure; 5) the configuration of girdle bands is a common condition; 6) the life history of *Ulnaria* can be divided into the four series of successive stages: auxospore, initial cell, pre-normal vegetative cell, and normal vegetative cell, which is very similar to the life history of *Hannaeainaequidentata* (Lagerstedt) Genkal and Kharitonov; 7) the closed valvocopula is proposed as a definition character for the genus *Ulnaria* because demonstrating all girdle bands closed is impracticable.

## ﻿Introduction

Historically, the generic name “*Synedra*” has been used in two different senses by diatom researchers from around the world. This situation continued until Compère (2001) proposed that *Synedra*, lectotypified by *S.balthica* Ehrenberg [=*S.gaillonii* (Bory) Ehrenberg], would remain the correct name and to use *Ulnaria* at the genus name for freshwater taxa associated with *S.ulna* (Nitzsch) Ehrenberg and *S.ungeriana* (Grunow) D.M. Williams. This study focuses on the *Ulnaria* from China. However, there were no diatom taxa from China placed in the genus *Ulnaria* until Williams (2011) transferred Synedraulnavar.repanda Q.X. Wang & Q.M You into *Ulnariarepanda* (Q.X. Wang & Q.M You) Williams. Thus, first the study history of *Synedra* in China is reviewed because possible *Ulnaria* taxa may have been placed in the genus *Synedra* before 2011 and even after 2011.

The history of diatom studies in China from 1848 through 2019 has recently been documented in detail by Kociolek et al. (2020). Sixteen new *Synedra* taxa were described in China from 1906 to 2008 by Mereschkowsky (1906), Skvortzov (1928, 1935, 1938), Yang (1990), Bao and Reimer (1992), Liu (1994), and You et al. (2008) (Table 1, see also Kociolek et al. 2020). All of these taxa need further investigations to assess their statuses. As far as is known, none of the original material for new taxa described by either Mereschkowsky or Skvortzov has been preserved (Williams et al. 2016), so any further determination is impossible. It is difficult to judge what genus Synedraulnavar.anhuiensis Yang and S.mazamaensisvar.changbaiensis Bao and Reimer would currently belong to. Although S.ulnavar.repanda has been transferred into *U.repanda*, its irregular valves (see You et al. 2008, p. 420, fig. 1) may belong to the pre-normal valves rather than the normal valves following the definition of pre-normal valve by Liu and Williams (2020).

**Table 1.** 16 new *Synedra* taxa described in China from 1906 to 2008.

No.	Taxon	Reference
1	Synedraulnavar.intermedia Mereschkowsky	Mereschkowsky 1906
2	Synedraulnaf.curta Skvortzov	Skvortzov 1928
3	Synedraulnavar.mongolica Skvortzov	Skvortzov 1928
4	Synedraaffinisvar.sinica Skvortzov	Skvortzov 1935
5	Synedraamphicephalavar.asiatica Skvortzov	Skvortzov 1935
6	*Synedralicenti* Skvortzov	Skvortzov 1935
7	Synedrarumpensvar.sinica Skvortzov	Skvortzov 1935
8	Synedrateneravar.sinica Skvortzov	Skvortzov 1935
9	Synedraulnaf.constricta Skvortzov	Skvortzov 1938
10	Synedraulnavar.lanceolataf.constricta Skvortzov	Skvortzov 1938
11	Synedraulnavar.tenuirostris Skvortzov	Skvortzov 1938
12	Synedravaucheriaevar.capitata Skvortzov	Skvortzov 1938
13	Synedraulnavar.anhuiensis Yang	Yang 1990
14	Synedramazamaensisvar.changbaiensis Bao and Reimer	Bao and Reimer 1992
15	*Synedracyclophoroides* S.C. Liu	Liu 1994
16	Synedraulnavar.repanda Q.X. Wang & Q.M. You	You et al. 2008

Except for the above new *Synedra* taxa described in China, many known *Synedra* taxa were also reported in China. Fifty-one *Synedra* taxa which were illustrated and had some descriptions are listed in Table 2. The acceptance of the generic name *Ulnaria* is a slow process with Chinese diatom researchers. Qi and Li (2004), in their authoritative monograph, still used “*Synedra*” in two different senses three years after *Ulnaria* was established (Compère 2001). In Qi and Li (2004, see Table 2), *Synedragaillonii* (Bory) Ehrennerg should remain in *Synedra*, but *S.ulnaria* should be placed in *Ulnaria* as *U.ulna*. Most of the 51 taxa have only the illustrations of line drawings and lack the observations of scanning electron microscopy, so they also need further investigations to assess their statuses.

**Table 2.** *Synedra* taxa reported from China.

No.	Taxon	Reference
1	*S.acus* Kützing	Hu et al. 1980
2	S.acusvar.angustissima Grunow	Deng 1983
3	S.acusvar.radians (Kützing) Hustedt	Zhu and Chen 2000
4	*S.affinis* Kützing	Hu et al. 1980
5	*S.amphicephala* Kützing	Hu et al. 1980
6	S.amphicephalavar.austriaca (Grunow) Hustedt	Zhu and Chen 2000
7	S.amphicephalavar.intermedia Cleve-Euler	Zhu and Chen 2000
8	*S.berolinensis* Lemmermann	Qi and Li 2004
9	*S.capitata* Ehrenberg	Deng 1983, Huang et al. 1983
10	*S.dorsiventralis* Otto Müller	Zhu and Chen 2000
11	*S.familica* Kützing	Qi and Li 2004
12	*S.fasciculata* (C. Agardh) Kützing	Li et al. 2005
13	*S.gaillonii* (Bory) Ehrennerg	Qi and Li 2004
14	*S.goulardii* Brebisson	Qi and Li 2004
15	*S.investiens* W. Smith	Qi and Li 2004
16	*S.mazamaensis* Soverergn	Qi and Li 2004
17	*S.minuscula* Grunow	Qi and Li 2004
18	*S.montana* Krasske	Qi and Li 2004
19	*S.nana* Meister	Qi and Li 2004
20	*S.parasitica* (W. Smith) Hustedt	Zhu and Chen 2000
21	S.parasiticavar.subconstricta (Grunow) Hustedt	Zhu and Chen 2000
22	*S.pulchella* (Ralfs ex Kützing) Kützing	Qi and Li 2004
23	*S.robusta* Ralfs	Jin et al. 1982
24	*S.rumpens* Kützing	Zhu and Chen 2000
25	S.rumpensKützingvar.familiaris (Kützing) Hustedt	Huang et al. 1983
26	S.rumpensKützingvar.meneghiniana Grunow	Deng 1983, Zhu and Chen 2000
27	S.rumpensKützingvar.scotica Grunow	Zhu and Chen 2000
28	S.rumpensKützingvar.sinica Skvortzov	Qi and Li 2004
29	*S.socia* Wallace	Qi and Li 2004
30	*S.tabulata* (Agardh) Kützing	Qi and Li 2004
31	S.tabulatavar.fasciculata (C.A. Agardh) Hustedt	Deng 1983
32	S.tabulatavar.parava (Kützing) Hustedt	Qi and Li 2004
33	S.tabulatavar.obtusa (Arnott) Hustedt	Zhu and Chen 2000
34	S.tabulatavar.rostrata (Juhlin-Dannfelt) Cleve-Euler	Zhu and Chen 2000
35	S.tabulatavar.genuina Cleve-Euler	Deng 1983
36	*S.tenera* W. Smith	Zhu and Chen 2000
37	*S.ulna* (Nitzsch) Ehrenberg	Hu et al. 1980
38	S.ulnavar.aequalis (Kützing) Brun	Zhu and Chen 2000
39	S.ulnavar.amphirhynchus (Ehrenberg) Grunow	Zhu and Chen 2000
40	S.ulnavar.biceps (Kützing) Schönfeldt	Deng 1983
41	S.ulnavar.chaseana Thomas	Qi and Li 2004
42	S.ulnavar.constracta Østrup	Zhu and Chen 2000
43	S.ulnavar.danica (Kützing) Grunow	Deng 1983
44	S.ulnavar.danicaf.continua (Kützing) Grunow	Deng 1983
45	S.ulnavar.impressa Hustedt	Qi and Li 2004
46	S.ulnavar.oxyrhynchus (Kützing) Van Heurck	Zhu and Chen 2000
47	S.ulnavar.oxyrhynchusf.constricta Skvortzow	Zhu and Chen 2000
48	S.ulnavar.ramesi (Héribaud) Hustedt	Qi and Li 2004
49	S.ulnavar.spathulifera (Grunow) Van Heurck	Zhu and Chen 2000
50	S.ulnavar.splendcns (Kützing) Grunow	Deng 1983
51	*S.vaucheriae* (Kütz.) Kützing	Hu et al. 1980

From 2017 to 2020, nine new *Ulnaria* species found in China were published (Liu et al. 2017, 2019a, b; Williams and Blanco 2019, 2020; Table 3). These nine new species were well documented through observations with light and scanning electron microscopy, and three of which were found to possess mostly biseriate striae (*U.ulnabiseriata* D.M. Williams & Bing Liu, *U.gaowangjiensis* Bing Liu & D.M. Williams and *U.oxybiseriata* D.M. Williams & Bing Liu). The observation of girdle bands on seven of the nine new *Ulnaria* species greatly expanded our understanding of these for this genus.

**Table 3.** Nine new *Ulnaria* taxa described in China from 2017 to 2020.

No.	Taxon	Reference
1	*U.sinensis* Bing Liu et D.M. Williams	Liu et al. 2017
2	*U.ulnabiseriata* D.M. Williams et Bing Liu	Liu et al. 2017
3	*U.gaowangjiensis* Bing Liu et D.M. Williams	Liu et al. 2017
4	*U.rhombus* D.M. Williams	Liu et al. 2019a
5	*U.wulingensis* Bing Liu	Liu et al. 2019a
6	*U.oxybiseriata* D.M. Williams & Bing Liu	Liu et al. 2019b
7	*U.jinbianensis* S. Blanco & Bing Liu	Liu et al. 2019b
8	*U.dongtingensis* Bing Liu	Liu et al. 2019b
9	*U.hunanensis* Bing Liu	Liu et al. 2019a, Williams and Blanco 2019

Although *Ulnaria* was established in 2001, the use of the name *Ulnaria* is currently a nomenclatural decision (Van de Vijver and Cocquyt 2009) because *Ulnaria* does not have a clear definition due to the type species *Ulnariaulna* being an uncharacterized species (Morales et al. 2014). To get a clearer understanding of *U.ulna*, Lange-Bertalot and Ulrich (2014) investigated *Bacillariaulna* Nitzschia (the basionym of *U.ulna*). But no original material can be found, and the type locality does not exist anymore. They then epitypified *B.ulna* (Lange-Bertalot and Ulrich 2014, p. 66). From their description of the epitype, two important points are worth paying attention to: one is most of the frustules lay in girdle view and the other is girdle bands are closed – which was provided as a defining feature for *Ulnaria* by Williams (2011), who considered the studied data of *Ulnaria* and stated, “In the case of any taxon including *S.ulna*, closed girdle bands are of significance: they are hypothesized as a synapomorphy for that group, one of its defining features”. A lot of research on *Ulnaria* has proved that the presence of closed girdle bands is a useful differentiating character from other similar genera (e.g., Morales et al. 2014; Liu et al. 2017; Cantonati et al. 2018; Liu et al. 2019a, b; Williams 2020). However, closed girdle bands in some published *Ulnaria* species were not confirmed by the authors [e.g., *U.macilenta* E. Morales, C.E. Wetzel & S.F. Rivera (Morales et al. 2014); *U.verhaegeniana* Van de Vijver, M. de Haan, Mertens & Cocquyt (Van de Vijver et al. 2017)]. For these cases, Van de Vijver et al. (2017, p. 223) claimed that “the general appearance, the presence of two rimoportulae, the characteristic ocellulimbus and the internal structure of the striae and areolae” can be diagnostic characters for *Ulnaria*. To date, according to the Algaebase.org website (Guiry and Guiry 2022), there are approximately 50 taxa accepted taxonomically in the genus *Ulnaria*. Reports of the phylogeny of *Ulnaria* are rare. Kulikovskiy et al. (2016) carried out a phylogenetic analysis of two new *Ulnaria* species and their result showed that *Ulnaria* is a closely related but independent branch from *Fragilaria* Lyngbye *sensu stricto*. Similarly, Zakharova et al. (2020) conducted a phylogenetic analysis of two clones from the genus *Ulnaria* and their result supported that *Ulnaria* is an independent clade. Both previous phylogenetic studies were based on only one gene (*rbc*L) fragments of five *Ulnaria* species.

Appendices 1–3 summarized the main morphological characters for 63 *Ulnaria* taxa, 49 of which have been published and 14 of which are first described in this paper. Many of these 63 *Ulnaria* taxa are well-documented so that we can see a wide range of morphological diversity in the genus *Ulnaria*. Firstly, the cell sizes can be very different among the *Ulnaria* taxa due to the valve length varying between 14–512 μm and the valve width between 2.5–12 (15) μm. Secondly, there are seven *Ulnaria* species producing valve marginal spines (Appendix 1). Interestingly, these seven species all possess uniseriate striae, i.e., so far, no *Ulnaria* species bearing biseriate striae are found to produce valve marginal spines (see Appendix 2). Thirdly, 14 *Ulnaria* species possess mostly biseriate striae (Appendix 2). Among these 14 species, only *U.gusliakovii* Genkal, Shcherbak & Semenyuk lacks a central area (Genkal et al. 2022) and the other 13 taxa have different shapes of central areas (Appendix 2). Finally, 42 *Ulnaria* taxa have uniseriate striae but do not produce marginal spines (Appendix 3). *Ulnaria* has been defined by the possession of closed girdle bands. However, the present situation is that there are only 40 *Ulnaria* species which were found to possess closed valvocopula or girdle bands and the nature of girdle bands for the other 23 *Ulnaria* species is still unknown (see Appendices 1–3). Hopefully, similar to this study, researchers will try to document the structures of girdle bands in their future studies of *Ulnaria*.

## ﻿Materials and methods

### ﻿Site description and sampling

The diatom samples studied herein were collected from the Wuling Mountains, Dongting Lake and Qinghai province beginning in 2014. The Wuling Mountains, which stretch across Chongqing, Hunan, Hubei, and Guizhou provinces, are one of the ten biodiversity hotspot ecoregions considered as conservation priorities in China (Tang et al. 2006). It belongs to the mountain climate, which is a transition from the sub-tropical to the warm temperate climate (see Liu et al. 2017 for further information). Dongting Lake is the second largest freshwater lake in China and is located between 28°30'N–30°20'N, 111°40'–113°40'E in the northeast part of Hunan province (see Liu et al. 2018 for further details). Qinghai is an inland province of China, which has an average elevation greater than 3000 m a.s.l. The plateau accounts for 80% of Qinghai landscape so its climate belongs to the plateau continental climate. The diatom samples from Wuling Mountains and Qinghai province were collected from the surfaces of 3–7 submerged stones from each sampling cite; the diatom samples from Dongting Lake were collected from its littoral zone topsoil by scraping the 1–2 mm surface sediment. During sample collection, temperature, pH, and conductivity were measured *in situ* (three repetitions) with a portable multimeter (HQ40D, HACH Company).

The collected diatom samples which were not added 70% alcohol were used to observe the living cells. 100 μl diatom samples were transferred into a round chamber (diameter 14 mm, depth 0.35 mm) located in the middle of a custom-made slide by using a pipette, then examined using a Leica DM3000 light microscopy (LM) equipped with a Leica MC190 HD digital camera. The collected diatom samples which were added 70% alcohol were processed (cleaned) for microscopic examination with 10% HCl and 30% H_2_O_2_. Permanent slides were prepared using Naphrax mountant and examined using the same light microscopy as above. Slides are deposited in the Herbarium of Jishou University, Hunan, People’s Republic of China (JIU) (Herbarium acronyms follow Index Herbarium http://sweetgum.nybg.org/science/ih/). Samples were also examined using scanning electron microscopy (SEM). Several drops of the cleaned diatom material were air-dried onto glass coverslips. The coverslips were attached to aluminium stubs using double-sided conductive carbon strip and sputter-coated with platinum (Cressington Sputter Coater 108auto, Ted Pella, Inc.). Samples were examined and imaged using a field emission scanning electron microscopy (FESEM) Sigma HD (Carl Zeiss Microscopy) available at Huaihua University, China.

### ﻿Morphological terminology

**Figure 1.**
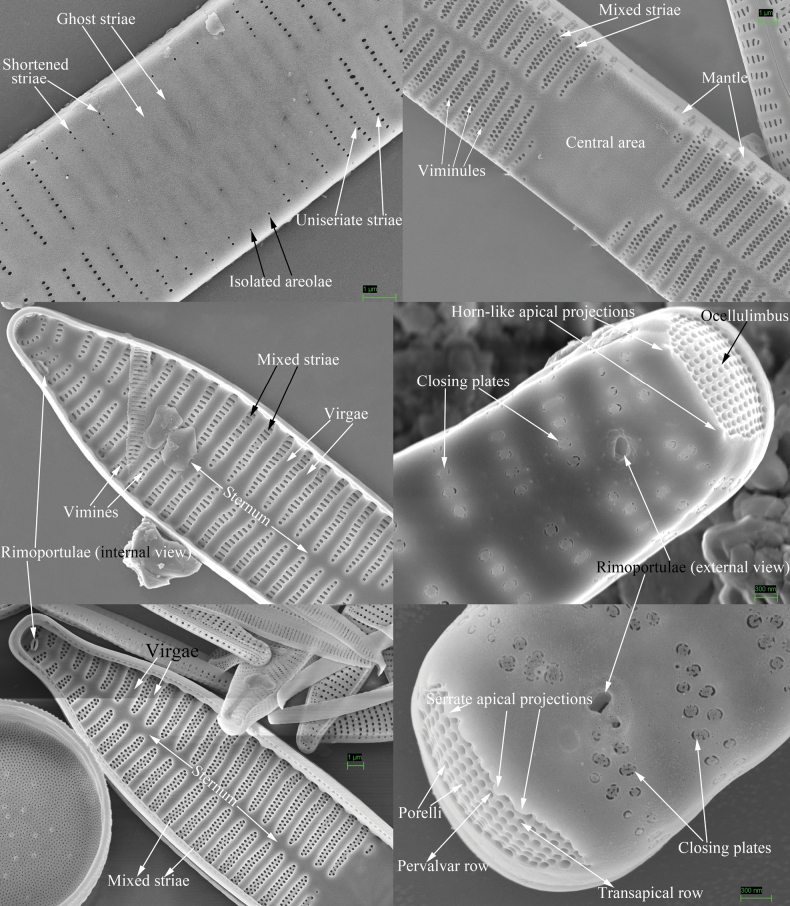
Valve characters used in the descriptions of *Ulnaria* taxa.

**Figure 2.**
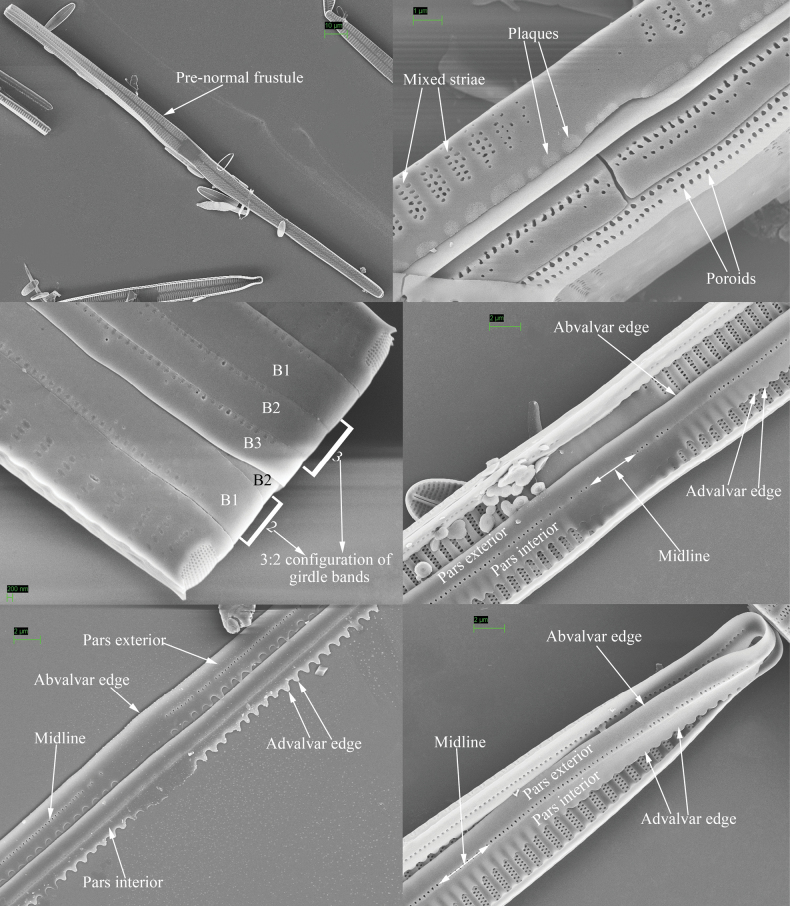
Characters of valve and girdle bands used in the descriptions of *Ulnaria* taxa.

Morphological terminology follows Anonymous (1975), Ross et al. (1979), Mann (1982), Williams (1986), Round et al. (1990), Cox (2012), and Lange-Bertalot and Ulrich (2014). Life cycle terminology mostly follows Kaczmarska et al. (2013) and Liu and Williams (2020). To avoid any ambiguity and misunderstanding, the terms most used in this study are illustrated in Figs 1 and 2, a part of them are defined below, and other common terms are not repeated here.

Valvocopula abvalvar edge: the edge that is farther from the valve face.

Valvocopula advalvar edge: the edge that is closer to the valve face.

Closing plate: a plate that does not completely occlude the outside opening of areola. This plate is usually solid (sometimes with several perforations) and has a few struts attaching it to each areolar wall.

Configuration of girdle bands: the ratio between the number of girdle bands associated with the epivalve and the number associated with the hypovalve in a diatom cell.

Ghost striae: the faint “striae” composed of unperforated grooves in the central area of the valve interior (visible under SEM). These grooves look like “striae” under LM but are not true striae.

Mixed stria: the striae are usually divided into three types: uniseriate, biseriate and multiseriate striae. A mixed stria is a stria composed of at least two of the previous three stria types.

Pre-normal vegetative frustule: the pre-normal vegetative period is the time between immediately after the initial cell’s first division and the presence of the first new normal vegetative cells. The frustule occurring during this period is termed pre-normal vegetative frustule.

Vimines (s. vimen): the cross-connecting tiny ribs between two adjacent virgae defining areolae in uniseriate striae.

Viminules (s. viminule): the interconnecting tiny ribs between two adjacent virgae which define areolae in the biseriate or multiseriate striae.

Virga (pl. virgae): the transverse silica rib between two adjacent striae.

## ﻿Results

### ﻿Life history of *Ulnaria* – a case study from *U.ulnabiseriata*

(Figs 3–13)

**Figure 3.**
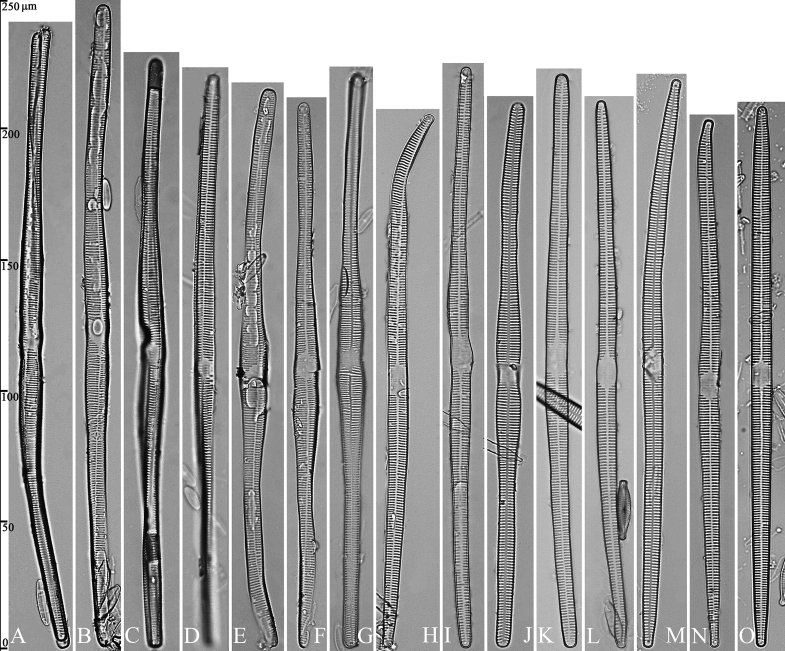
Life history of *Ulnariaulnabiseriata*, from initial cell, via pre-normal cells, finally to normal cell, ×400, LM**A** initial frustule showing its cylindrical and twisted outline, central area flanked by striae on one side **B–N** pre-normal frustules, note twisted frustules with laterally located sterna (**B–E**), one apex twisted and with almost centrally located sterna (**F, G**), almost centrally located sterna and complete central areas (**H–N**). **O** Normal valve.

**Figure 4.**
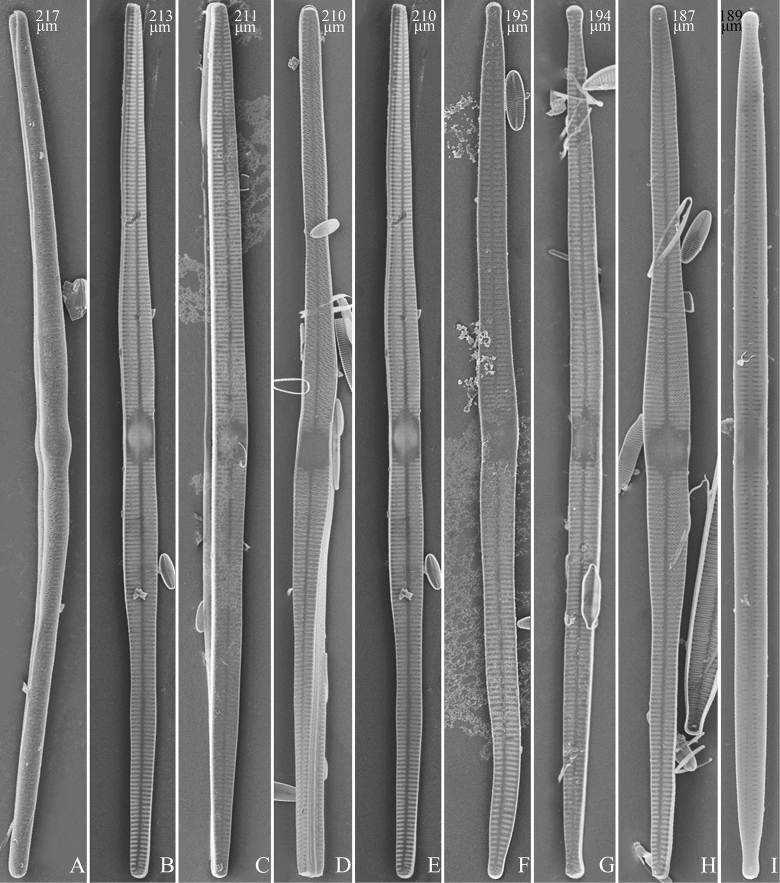
Life history of *Ulnariaulnabiseriata*, from initial cell, via pre-normal cells, finally to normal cell, external view, SEM**A** initial frustule, note its cylindrical and twisted frustule (for details see Fig. 5) **B–H** pre-normal frustules/valves, note twisted frustules with smoothly tapering apices (**B–E, H**), protracted and sub-capitate to rostrate apices (**F, G**) **I** normal valve. Note: the number value at the top of each figure refers to the length of each specimen.

The life history of *Ulnaria* can be divided into the following four series of successive stages: auxospore, initial cell, pre-normal vegetative cell, and normal vegetative cell. Unfortunately, we did not find an auxospore of *Ulnaria*, but the initial cell, pre-normal vegetative cell, and normal vegetative cell of *U.ulnabiseriata* were all documented using LM and SEM. The life history, from the initial cell, via pre-normal vegetative cells, to normal vegetative cell, is a process from chaos to order, i.e., the shapes of initial cell and pre-normal vegetative cells gradually become more and more regular and symmetrical from irregular and asymmetrical (see Figs 3, 4, 9).

**Figure 5.**
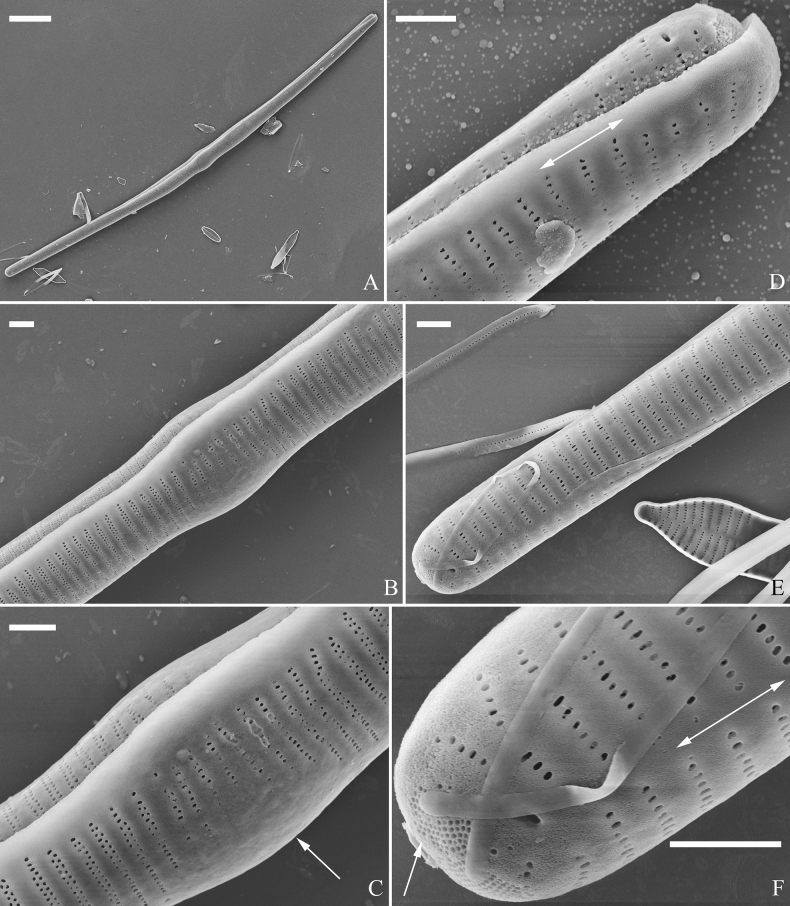
Initial frustule of *Ulnariaulnabiseriata*, external view, SEM**A** Initial frustule, note its arcuate, cylindrical, and twisted outline **B, C** details of middle part from **A**, note small central area flanked by striae on one side (**C**, arrow), mixed striae, and underdeveloped sternum (i.e., striae continue across valve surface) **D** apical detail from **A**, note mantle not well differentiated from valve face (double-headed arrow), and virgae and vimines/viminules occurring nearly on the same plane **E, F** other apical details from **A**, note laterally located sternum present only near one apex (**F**, double-headed arrow), ocellulimbus flush with and extending onto valve surface, and its partial pervalvar rows of porelli not perpendicular to the valve plane (**F**, arrow). Scale bars: 20 μm (**A**); 2 μm (**B–F**).

Initial cells: Two initial cells were found and measured. One is 240 μm long (Fig. 3A), the other is 217 μm long (Fig. 4A). The initial frustule has an arcuate, cylindrical, and twisted outline (Figs 3A, 4A, 5A). It has a small central area flanked by some striae on one side (Fig. 5C, arrow), the mixed striae are composed of partial biseriate and partial uniseriate, the mantle is not well differentiated from the valve face (Fig. 5D, double-headed arrow), the valve lacks a sternum except at one apex where a short laterally located sternum is produced (Fig. 5F, double-headed arrow). The ocellulimbus is not inset yet, extending onto the valve surface, and its partial pervalvar rows of porelli are not vertical (Fig. 5F, arrow). Perizonium and girdle bands are not observed.

**Figure 6.**
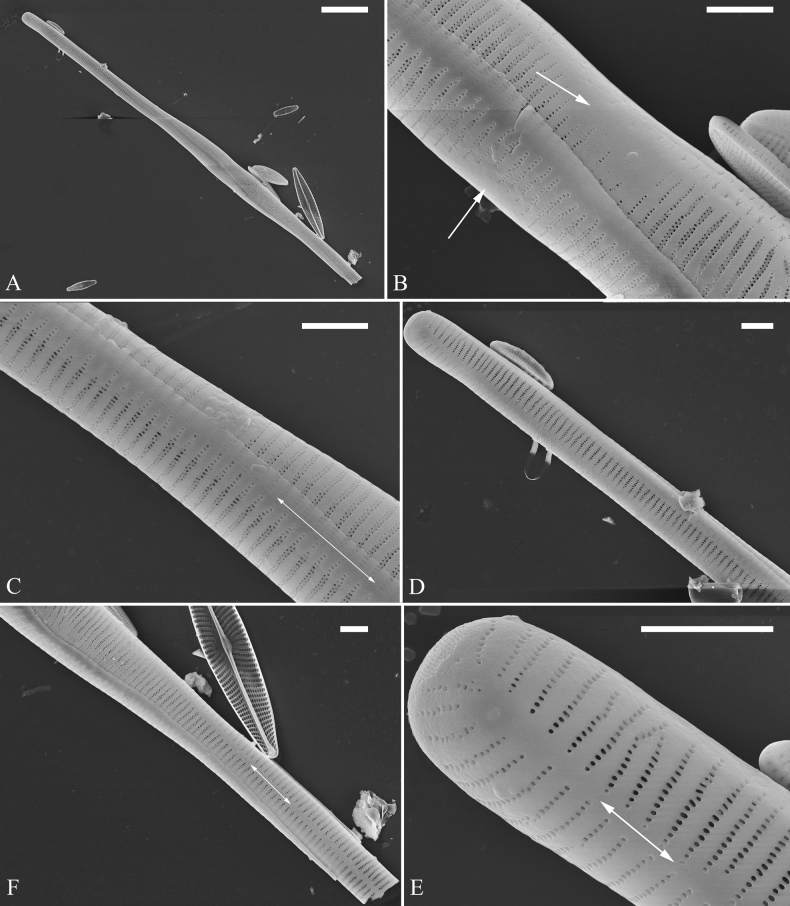
Pre-normal frustule of *Ulnariaulnabiseriata*, external view, SEM**A** pre-normal frustule, note its cylindrical and twisted outline **B, C** middle part details from **A**, note small central area flanked by striae on one side (**B**, two arrows), girdle bands lacking **D, E** apical details from **A**, note laterally located sternum (**E**, double-headed arrow) and flush ocellulimbus **F** other half from **A**, note laterally located sternum (double-headed arrow). Scale bars: 20 μm (**A**); 4 μm (**B–F**).

**Figure 7.**
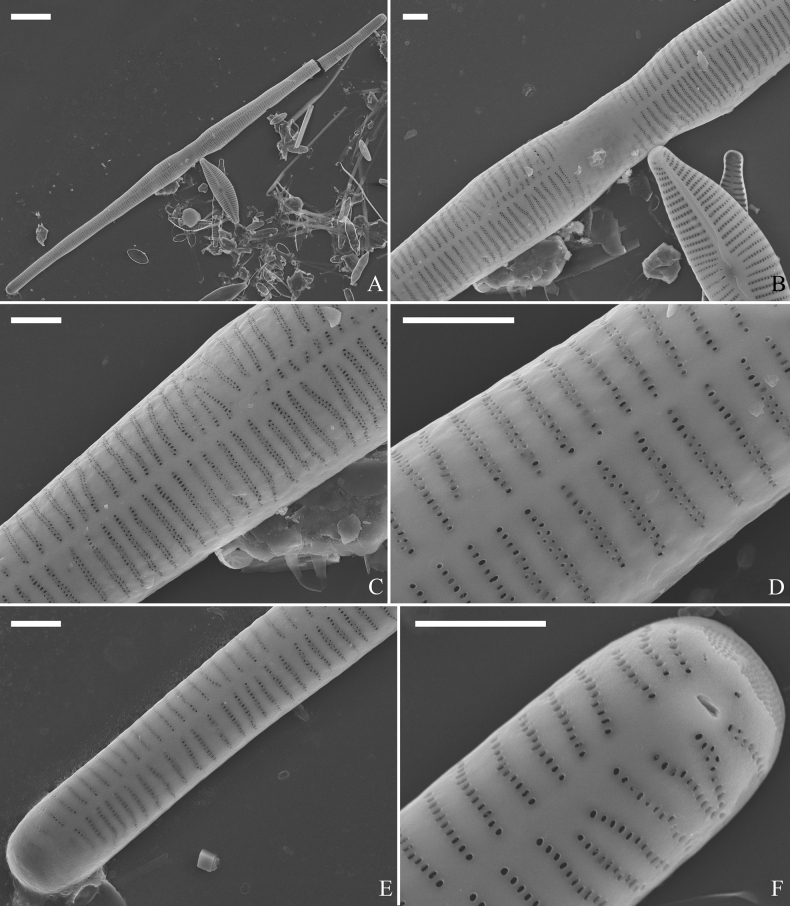
Pre-normal frustule of *Ulnariaulnabiseriata*, external view, SEM**A** pre-normal valve **B** middle part detail from **A**, note its constricted outline in the middle **C, D** proximal parts of valve from **A**, note closing plates underdeveloped **E, F** apical details from **A**, note slightly inset ocellulimbus. Scale bars: 20 μm (**A**); 3 μm (**B–F**).

**Figure 8.**
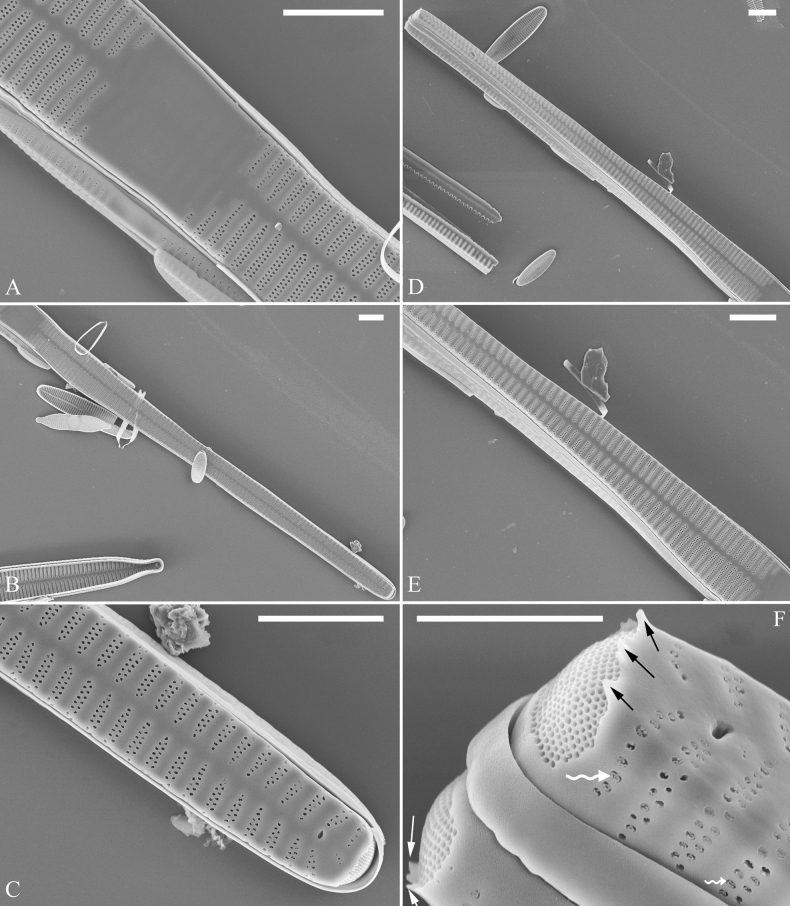
Details of late pre-normal frustule of *Ulnariaulnabiseriata* from Fig. 4D, external view, SEM**A** middle part detail, note rectangular central area **B** half valve, note its arcuate outline **C** one apical detail, note biseriate striae developed at apex **D, E** other half part of valve, note slightly twisted frustule and mantle joining the valve face at a right angle **F** apical detail from **D**, note inset ocellulimbus, developed closing plates (two wavy arrows), and few serrated apical projections protruding over the ocellulimbus (three black arrows and two white arrows). Scale bars: 6 μm (**A–E**); 3 μm (**F**).

**Figure 9.**
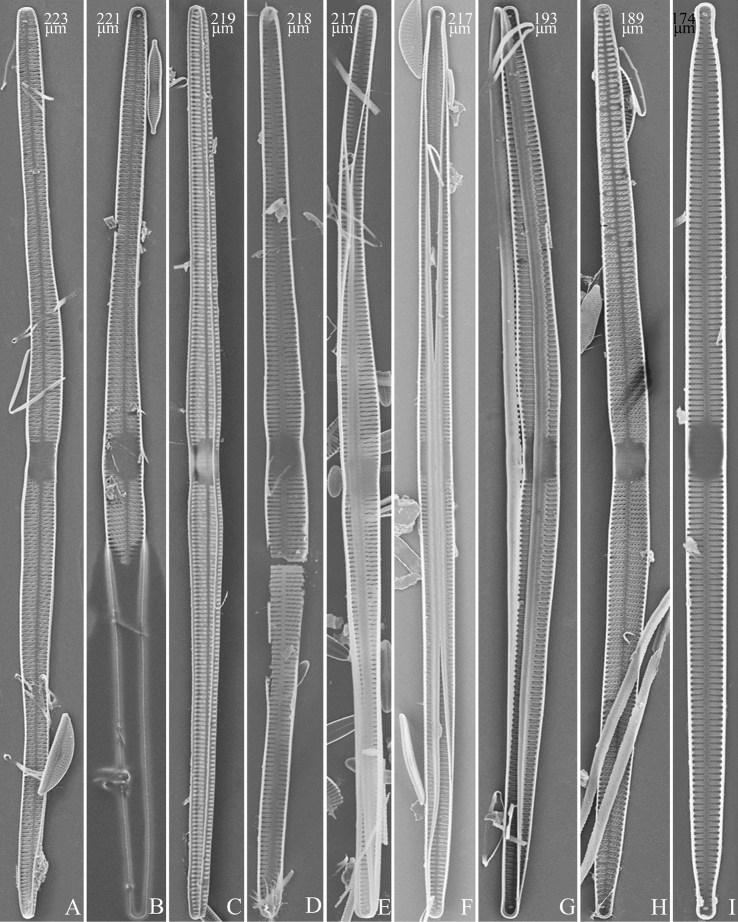
Valves of *Ulnariaulnabiseriata*, internal view, SEM**A–H** eight pre-normal valves, note irregular and asymmetrical valve outlines **I** normal valve, note its linear-lanceolate outline. Note: the number value at the top of each figure refers to the length of each specimen.

**Figure 10.**
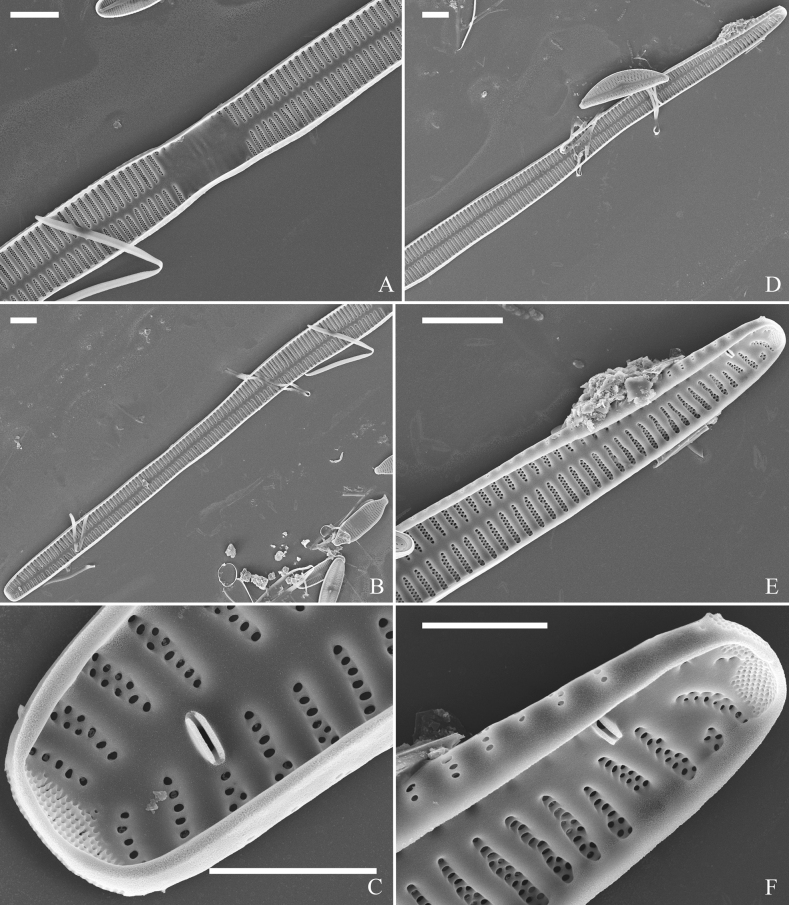
Details of late pre-normal value of *Ulnariaulnabiseriata* from Fig. 9A, internal view, SEM**A** middle constricted part of valve **B, C** details of one apex **D–F** details of other apex, note still slightly twisted valve. Scale bars: 6 μm (**A, B, D, E**); 3 μm (**C, F**).

**Figure 11.**
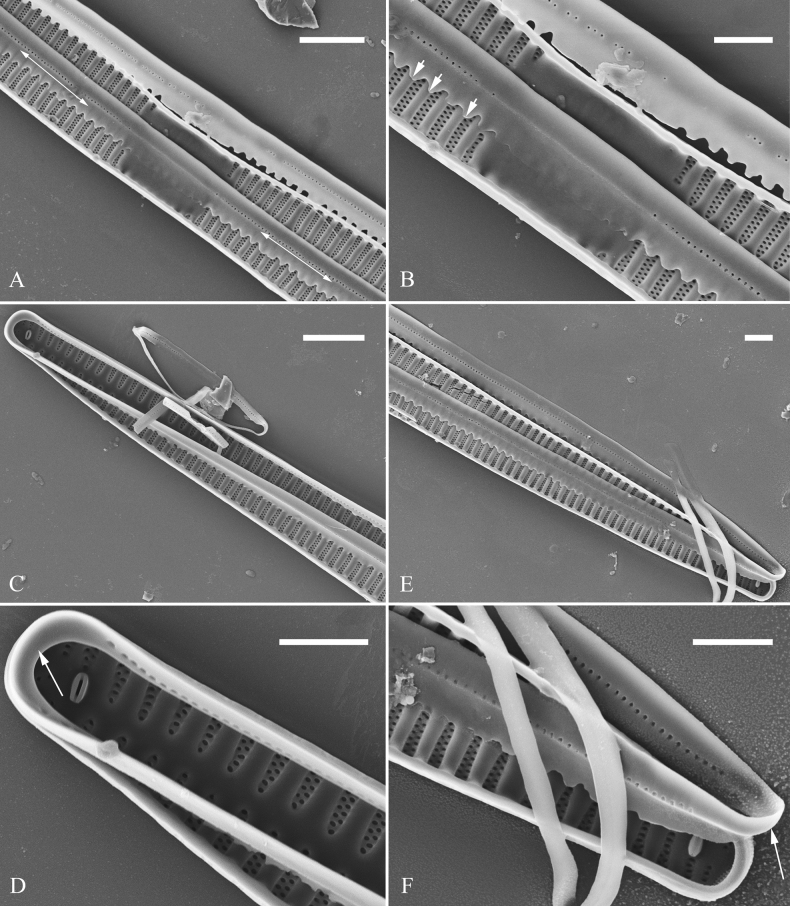
Details of late pre-normal valve of *Ulnariaulnabiseriata* from Fig. 9G, internal view, SEM**A, B** middle parts of valve, note a row of poroids located at midline (**A**, double-headed arrow) and a row of serrated projections aligned with each virga (**B**, three arrows) **C–F** apical details, note the closed two apices of valvocopula (**D, F**, arrow respectively). Scale bars: 6 μm (**A, C**); 3 μm (**B, D–F**).

Pre-normal vegetative cells: The valve length range of pre-normal vegetative cells is 196–250 μm (n = 41). Early pre-normal vegetative frustules may be twisted and have laterally located sterna (Fig. 3B–E), then progress to only one apex twisted with nearly centrally located sterna (Fig. 3F, G); finally, they progress to having centrally located sterna and complete central areas (Fig. 3H–N). Pre-normal vegetative frustules may also initially be twisted with smoothly tapering apices (Fig. 4B–E, H), then their apices become protracted to rostrate or sub-capitate (Fig. 4F, G). Figure 6 demonstrates the details of an early pre-normal frustules. This pre-normal frustule has a cylindrical and twisted outline (Fig. 6A), small central area flanked by striae on only one side (Fig. 6B), laterally located sternum and ocellulimbus which is flush with the surface of the valve (Fig. 6C–F). Figure 7 demonstrates the details of a late pre-normal frustule. This pre-normal frustule has a constricted outline in its middle part (Fig. 7A, B), almost centrally located sternum, underdeveloped closing plates and undifferentiated mantle (Fig. 7C, D), and uniseriate striae present at apices and slightly inset ocellulimbus (Fig. 7E, F). Figure 8 demonstrates the details of a likely late pre-normal frustule. This pre-normal frustule has slightly twisted outline and almost centrally located sternum, complete central area (Fig. 8A), well differentiated mantle (mantle met the valve surface at a right angle, Fig. 8B–E), biseriate striae present at apices (Fig. 8C), and developed closing plates (Fig. 8F, two wavy arrows), inset ocellulimbus with pervalvar rows perpendicular to the valve plane and few serrated apical projections protruding over it (Fig. 8F, three black arrows and two white arrows). Figure 9A–H shows the internal views of eight pre-normal valves. These eight pre-normal valves generally have irregular and asymmetrical outlines. Figure 10 demonstrates the internal details of a late pre-normal valve. This pre-normal valve has undulating valve margins (Fig. 10A, B, D), one apex slightly twisted (Fig. 10E), biseriate striae present at apices (Fig. 10C, F). Figure 11 demonstrates the details of a likely latest pre-normal valve with a valvocopula. This valvocopula has the same structure as that observed in the normal vegetative valve, i.e., the valvocopula is a closed hoop, surrounding whole valve margin, bearing a mostly continuous row of poroids dividing pars interior from pars exterior, located at midline (Fig. 11A–E), lacking ornamentation at both apices (Fig. 11D, F). Its advalvar edge has a row of serrated projections which are aligned with the virgae (Fig. 11B, three arrows).

**Figure 12.**
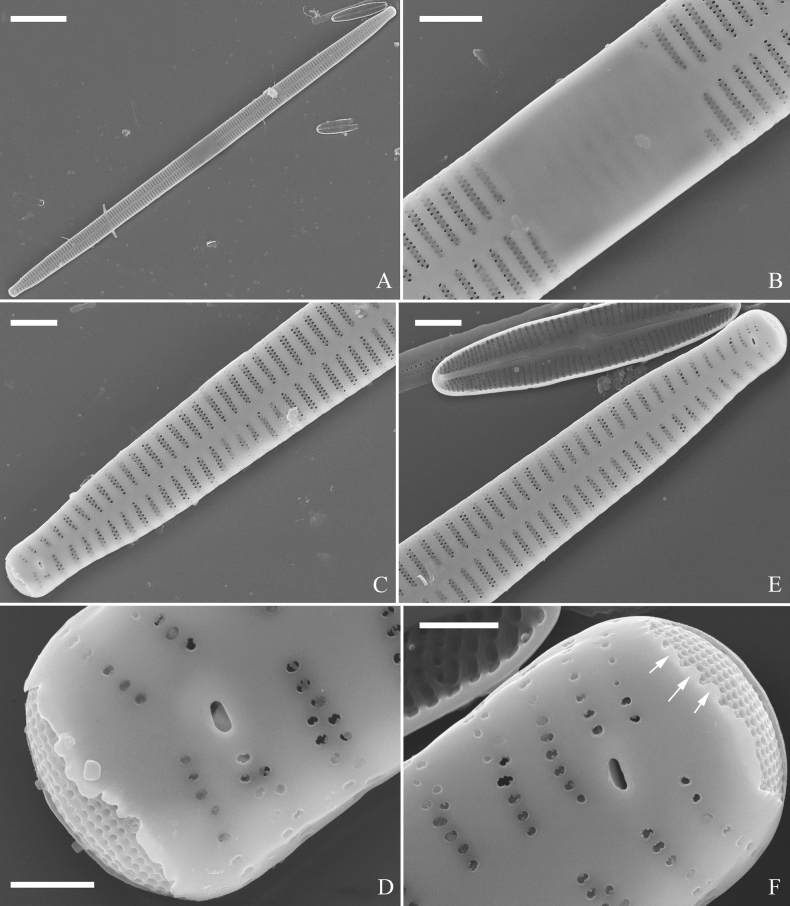
Normal valve of *Ulnariaulnabiseriata*, external view, SEM**A** complete valve **B** middle detail, note the rectangular central area **C, D** two apices, note centrally located sternum and rostrate apices **E, F** apical details, note inset ocellulimbus and some outgrowths protruding over it (**F**, three arrows). Scale bars: 20 μm (**A**); 3 μm (**B, C, E**); 1 μm (**D, F**).

**Figure 13.**
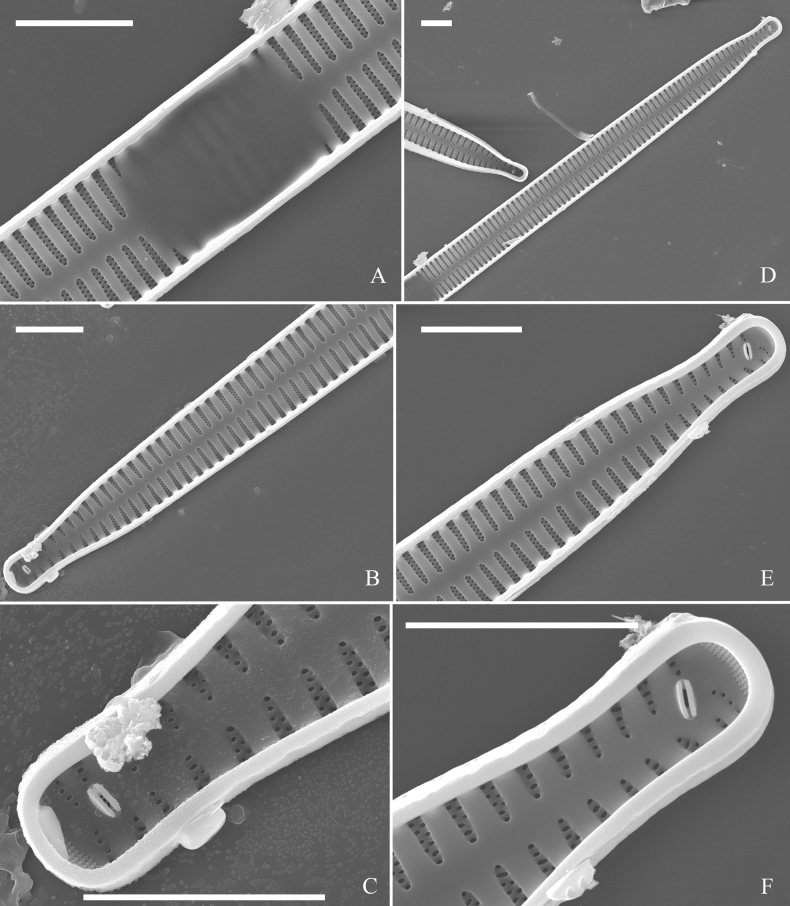
Details of normal valve of *Ulnariaulnabiseriata* from Fig. 9I, internal view, SEM**A** middle part, note parallel valve margins, rectangular central area **B–F** apical details, note centrally located sternum and virgae raised above from viminules. Scale bars: 6 μm (**A–F**).

Normal vegetative cells: The valves of *U.ulnabiseriata* (Figs 12 and 13) are characterized by their lanceolate outline, rectangular central area (Figs 12B, 13A), mostly biseriate striae (Figs 12C, E, 13B–F), well developed closing plates and ocellulimbus (Fig. 12D, F), and rostrate to sub-capitate apices (Figs 3O, 4I, 9I, 12A).

The morphological features that change during the life circle of *U.ulnabiseriata* are summarized in Table 4. From initial frustule/valve, via pre-normal vegetative frustule/valve, to normal vegetative frustule/valve, the girdle band numbers, valve outline, valve apices, sternum, central area, virgae and viminules, closing plates, and ocellulimbus, all gradually become normal (Table 4).

**Table 4.** Features of initial cell, pre-normal and normal vegetative frustule/valve in *U.ulnabiseriata*.

Feature	Initial frustule/valve	Pre-normal vegetative frustule/valve	Normal vegetative frustule/valve
Girdle bands	Not found	Not found to a few present	4 copulae associated with epivalve
Valve outline	Cylinder-like, twisted	Irregular and asymmetrical	Linear-lanceolate
Valve apex	Rounded	Rounded, rostrate, or sub-capitate	Rostrate to sub-capitate
Sternum	Non-existent except lateral sternum present only at apex	Lateral to nearly central sternum	Central sternum, i.e., normal, situated on the midline of valve
Central area	Present, small, with short striae at one side	From small to rectangular	Rectangular
Virga/viminule	Virgae and vimines/viminules almost flush with each other	Vimines/viminules slightly lower than virgae	Virgae raised, viminules sunken
Stria	Mixted striae in the middle, uniseriate striae near apex	Gradually become mostly biseriate striae (striae near apex become biseriate too)	Mostly biseriate striae
Closing plate	Not found	Not found to present	Well developed
Rimoportula number per valve	Two	Two	Two
Ocellulimbus	Extending on valve face, pervalvar row of porelli not perpendicular to the valve plane	Pervalvar rows of porelli gradually becoming perpendicular to the valve plane	Pervalvar rows of porelli all perpendicular to the valve plane

### ﻿Taxonomic treatment

#### ﻿Artificial key to 24 *Ulnaria* species described from China

**Table:** 

1	Biseriate striae	**2**
–	Uniseriate striae	**9**
2	Panduriform valve outline	**1. *U.pandurata-biseriata***
–	Not panduriform valve outline	**3**
3	Apiculate apices	**2. *U.oxybiseriata***
–	Not apiculate apices	**4**
4	Linear valve outline	**3. *U.wuling-biseriata***
–	Linear-lanceolate or lanceolate valve outline	**5**
5	Linear-lanceolate valve outline	**6**
–	Lanceolate valve outline	**7**
6	Constricted central valve margins with rostrate apex	**4. *U.constricta-biseriata***
–	Parallel central valve margins with rostrate apices	**5. *U.gaowangjiensis***
7	Slightly constricted central valve margins with capitate to sub-capitate apices	**6. *U.sangzhi-biseriata***
–	Parallel central valve margins	**8**
8	Central area apically rectangular	**7. *U.ulnabiseriata***
–	Central area very variable	**8. *U.jishou-biseriata***
9	Interlocking spines produced on valve margins	**9. *U.sinensis***
–	Interlocking spines lacking	**10**
10	Undulate valve outline	**10. *U.repanda***
–	Not undulate valve outline	**11**
11	Panduriform valve outline	**11. *U.pandurata-uniseriata***
–	Not panduriform valve outline	**12**
12	Parallel distal regions of valve present	**13**
–	Parallel distal regions of valve lacking	**15**
13	Rhombic valve outline	**12. *U.rhombus***
–	Not rhombic valve outline	**14**
14	Parallel distal region of valve more than 20 μm long	**13. *U.wulingensis***
–	Parallel distal region of valve less than 20 μm long	**14. *U.hunanensis***
15	Central area completely lacking	**16**
–	Central area present	**18**
16	Valve width less than 5 μm	**15. *U.qinghainensis***
–	Valve width more than 5 μm	**17**
17	Lanceolate valve outline	**16. *U.hupingensis***
–	Linear-lanceolate valve outline	**17. *U.xieriverensis***
18	Central area complete	**19**
–	Central area very variable	**21**
19	Linear-lanceolate valve outline	**18. *U.jinbianensis***
–	Lanceolate valve outline	**20**
20	Length of parallel central margins equal to the length of the central area	**19. *U.dongtingensis***
–	Length of parallel central margins much larger than the length of the central area	**20. *U.fanjingensis***
21	Capitate apices	**21. *U.neobiceps***
–	Not capitate apices	**22**
22	Linear-lanceolate valve outline	**22. *U.chengduoensis***
–	Lanceolate valve outline	**23**
23	Rostrate apices	**23. *U.menyuanensis***
–	Sub-capitate apices	**24. *U.blancoi***

### ﻿New species descriptions

#### ﻿ Ulnaria constricta-biseriata

Taxon classificationPlantaeLicmophoralesUlnariaceae

Bing Liu sp. nov.

67A4AC44-FDA2-54DD-AFE1-BC094E02D77A

**Figure 14.**
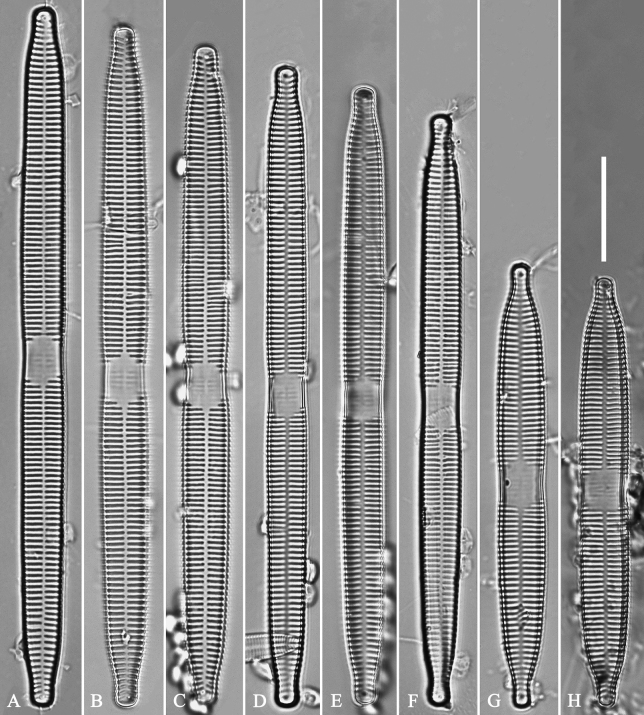
*Ulnariaconstricta-biseriata* sp. nov., ×1000, LM**A–H** eight valves exhibiting a size diminution series, note the constricted middle margins **A** micrograph of holotype specimen. Scale bar: 20 μm.

**Figure 15.**
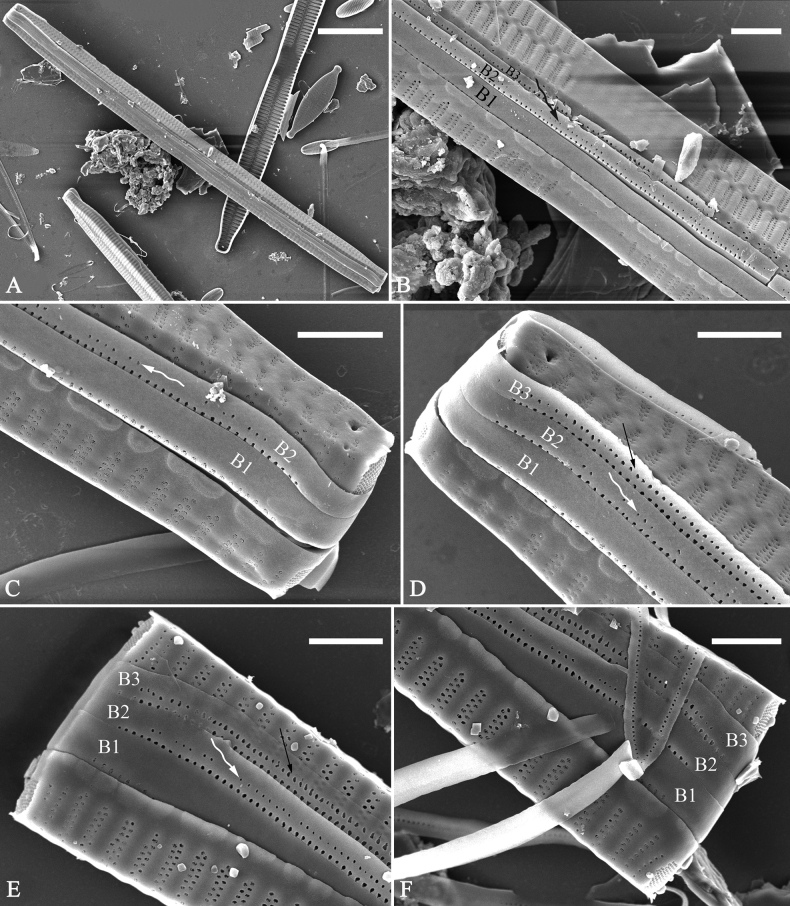
*Ulnariaconstricta-biseriata* sp. nov., girdle view, SEM**A** a frustule with a collapsed hypovalve **B–D** details from **A**, note three copulae (labeled B1 to B3) associated with the epivalves and two rows of poroids present on B2 (wavy arrows) and B3 (arrows) **E, F** the apices with three copulae visible (labeled B1 to B3) associated with the epivalves and two rows of poroids produced on B2 (wavy arrow) and B3 (arrow). Scale bars: 20 μm (**A**); 3 μm (**B–F**).

**Figure 16.**
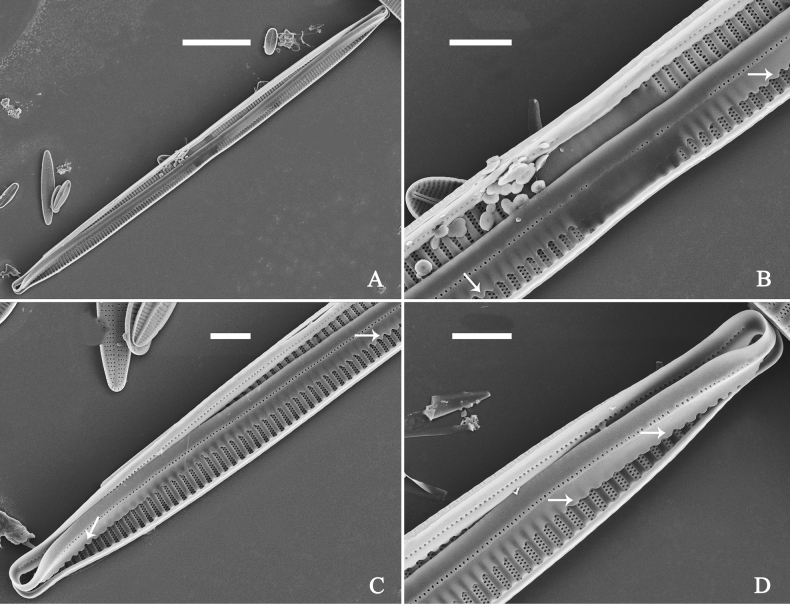
*Ulnariaconstricta-biseriata* sp. nov., internal view, SEM**A** a complete valve with a valvocopula **B** middle detail from **A**, note serrated projections at the advalvar edge (two arrows) **C, D** two apical details from **A**, note the closed nature of valvocopula and serrated projections at the advalvar edge (two arrows respectively). Scale bars: 20 μm (**A**); 4 μm (**B–D**).

**Figure 17.**
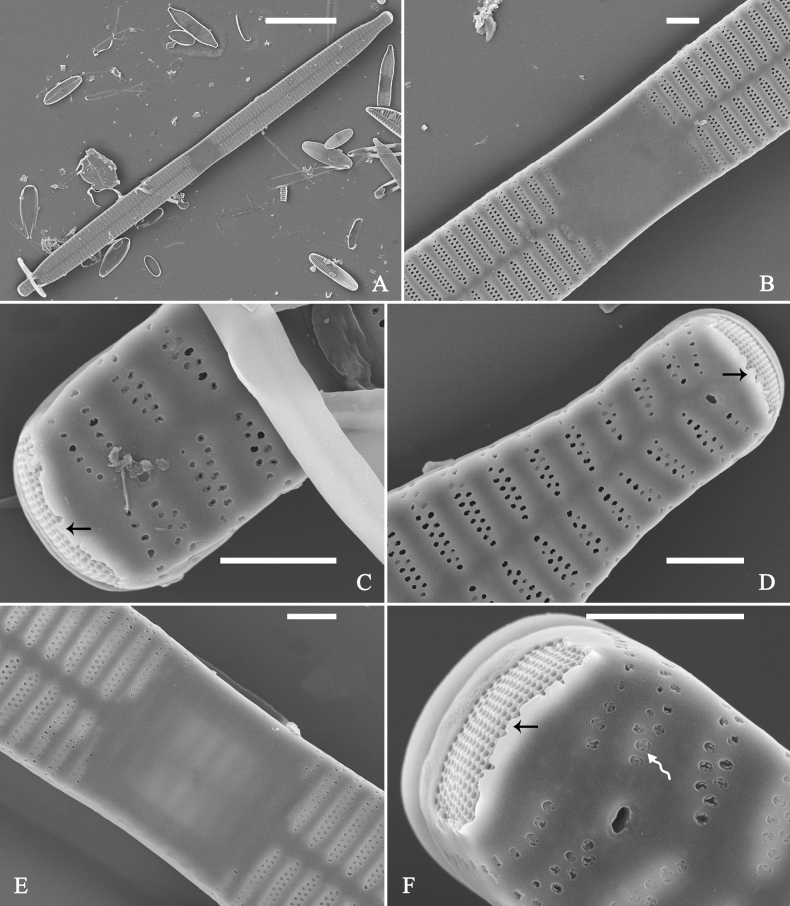
*Ulnariaconstricta-biseriata* sp. nov., external view, SEM**A** a complete valve **B** middle part from **A** showing the constricted middle margins and central area forming a rectangular hyaline region **C, D** two apical details from **A**, note a few serrated apical projections protruding over the ocellulimbus (two black arrows) **E** another middle part, note two constricted middle margins **F** another apex, note a few serrated apical projections protruding over the ocellulimbus (arrow) and the closing plates (wavy arrow). Scale bars: 20 μm (**A**); 2 μm (**B–F**).

**Figure 18.**
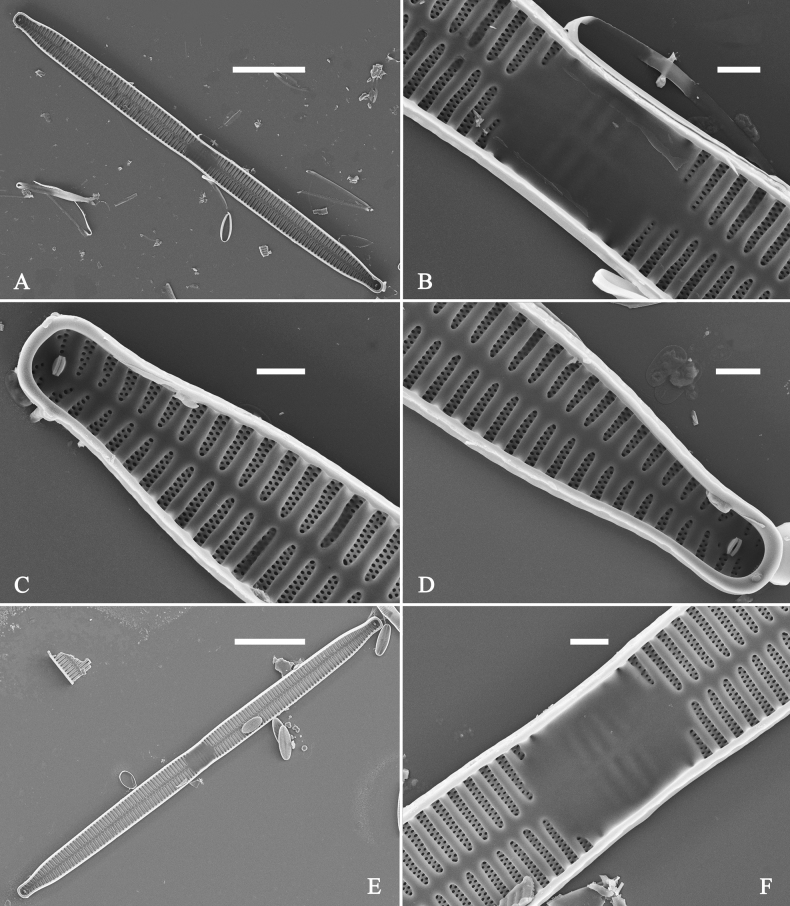
*Ulnariaconstricta-biseriata* sp. nov., internal view, SEM**A** a complete valve **B** middle part from **A**, note two constricted middle margins and some ghost striae **C, D** two apical details from **A**, note the striae mostly biseriate and two helictoglossae **E** another complete valve **F** middle detail from **E**, note two constricted middle margins and some ghost striae. Scale bars: 20 μm (**A, E**); 2 μm (**B–D, F**).

Figs 14 , 15 , 16 , 17 , 18

##### Holotype.

Slide JIUDIA202301, specimen circled on slide, illustrated as Fig. 14A.

##### Registration.

PhycoBank http://phycobank.org/103807

##### Type locality.

China. Guizhou province: Fanjing Mountain, Temple Longquan, Heiwan River (27°51'36"N, 108°45'51"E, 532 m a.s.l.), collected by Bing Liu, December 31, 2015.

##### Description.

***LM*** (Fig. 14). Valves linear-lanceolate, constricted at centre, apex rostrate to sub-capitate (Fig. 14A–H, see also Figs 16A, 17A, 18A, E). Valve dimensions (n = 35): length 66–166 μm, width 5.5–8 μm at constricted centre, narrower by 0.2–1.1 µm than at its widest. Valve margins parallel with rostrate to sub-capitate apices (Fig. 14A–H). Sternum central, distinct, extending whole length of valve. Rectangular or square central area. Ghost striae often present in central area (e.g., Fig. 14A–E). Striae parallel, radiate only at the poles. Striae mostly opposite one another across sternum. Stria density 10–12 (often 11) in 10 μm.

***SEM*** (Figs 15–18). Frustule in girdle view rectangular (Fig. 15A). Epivalve with up to three closed copulae (Fig. 16B–F), 2^nd^ and 3^rd^ copulae bearing two rows of poroids (2^nd^ = B2, Fig. 15B–E, white wavy arrow; 3^rd^ = B3, Fig. 15D, E, black arrow). Valvocopula closed, surrounding whole valve margin (Fig. 16A), bearing a mostly continuous row of poroids at the midline, dividing pars interior from pars exterior (Fig. 16B–D), lacking ornamentation at both poles (Fig. 16C, D); its advalvar edge having a row of serrated projections, each corresponding to a virga internally (Fig. 16B–D, two arrows respectively). Valve central area rectangular (Figs 17B, E, 18B, F). Striae constructed from series of relatively wide virgae interconnected with thin viminules, closing plates present with a few struts, affixed to the areolar wall (Figs 17B–F, 18B–D, F; closing plate see Fig. 17F, wavy arrow). Valves with mixed striae, mostly biseriate, formed by viminules; each stria situated opposite each other across sternum, equidistant until radiate at poles, and becoming uniseriate near sternum (Figs 17B–F, 18B–D, F). One rimoportula present at each pole, externally expressed as a simple hole (Fig. 17D, F), internally bilabiate, situated close to sternum (Fig. 18C, D). Apical pore fields ocellulimbus, consisting of ca. 23 pervalvar and 11 transverse rows of porelli (both pervalvar and transverse rows unequal in length). A few serrated apical outgrowths protruding over each ocellulimbus (Fig. 17C, D, F, one arrow respectively).

##### Etymology.

The specific epithet is formed from two terms: *constrict* and *biseriate*, reflecting the constricted valve central margins and the mostly biseriate striae of the valve.

##### Ecology and distribution.

The sampling site is close to the headwaters of the Heiwan River, which originates in the Fanjing Mountain National Nature Reserve. The diatom samples were scraped off of the stone surfaces. The following environmental parameters were measured in the field. Conductivity was 49.7 ± 0.2 μS∙cm^-1^, pH was 7.7 ± 0.1 and water temperature was 9.4 ± 0.1 °C. So far, its distribution is known only from the type locality. To sum up, *U.constricta-biseriata* lives on the stone surfaces of the headwaters of a mountainous river.

##### Discussion.

*Ulnariaconstricta-biseriata* is characterized by its linear-lanceolate valve outline, constricted valve central margins, and mostly biseriate striae. *Ulnariacontracta* (Østrup) E.A. Morales & M.L. Vis has also constricted valve central margins, but it differs from *U.constricta-biseriata* by its lanceolate valve outlines and mostly uniseriate striae (see Morales et al. 2007, p. 61, figs 48–55, p. 63, figs 56–61, as Synedraulnavar.contracta Østrup).

#### ﻿ Ulnaria jishou-biseriata

Taxon classificationPlantaeLicmophoralesUlnariaceae

Bing Liu sp. nov.

A7A935AD-ABDF-5126-AC85-0B9FFB073B15

**Figure 19.**
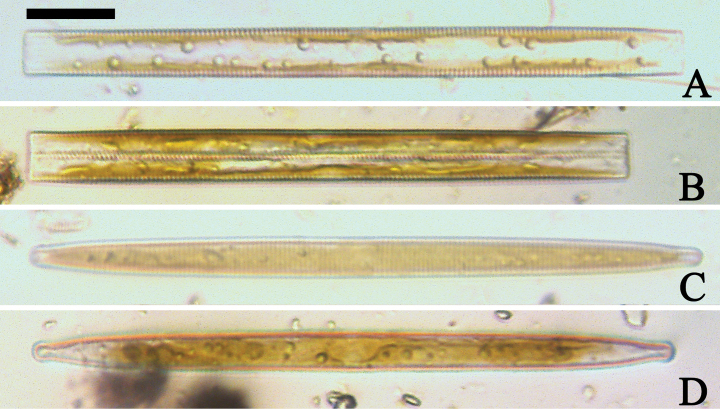
*Ulnariajishou-biseriata* sp. nov., ×400, LM**A** A cell in girdle view, note two long valve-appressed chloroplasts per cell **B** a dividing cell in girdle view, note the chloroplasts are distributed along the pervalvar axis **C, D** two cells in valve view, note the long plate of chloroplast. Scale bar: 20 μm.

**Figure 20.**
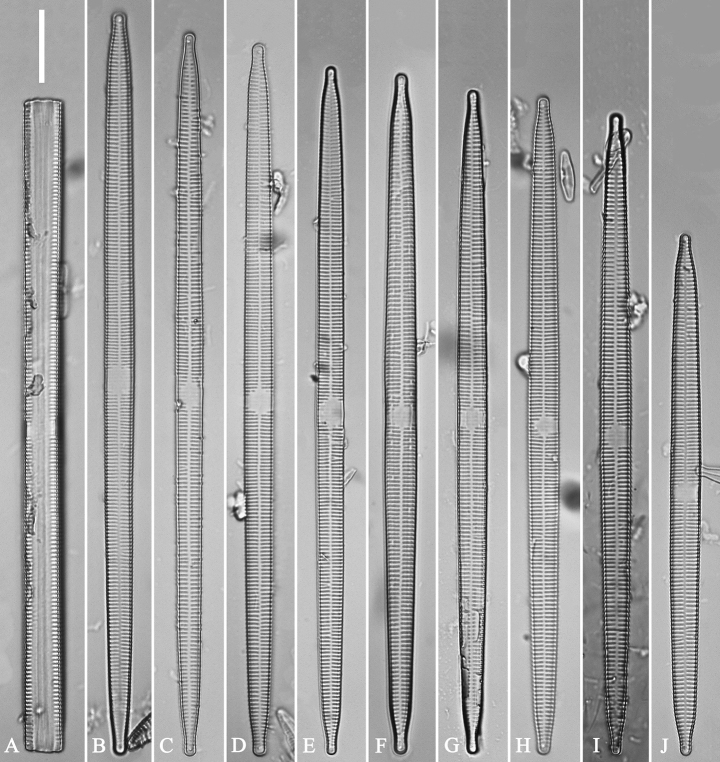
*Ulnariajishou-biseriata* sp. nov., ×630, LM**A** a frustule in girdle view, note the rectangle outline **B–J** nine valves exhibiting a size diminution series, note the lanceolate valve outline and the variable central areas **D** micrograph of holotype specimen. Scale bar: 20 μm.

**Figure 21.**
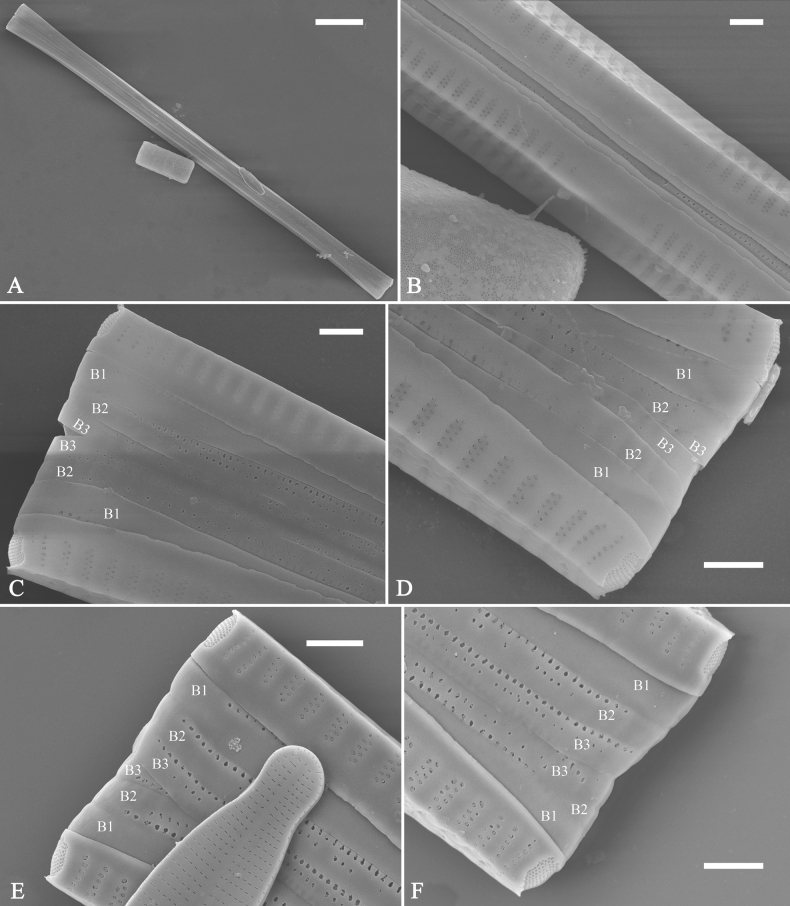
*Ulnariajishou-biseriata* sp. nov., girdle view, SEM**A** a dividing cell in girdle view **B** middle detail from **A**, note the mantles are hyaline in the central area **C, D** two apical details from **A**, showing both epivalve and hypovalve with associated three girdle bands (labelled B1 to B3) **E, F** two other apical details also show both epivalve and hypovalve with associated three girdle bands in a dividing cell (labelled B1 to B3). Scale bars: 20 μm (**A**); 2 μm (**B–F**).

**Figure 22.**
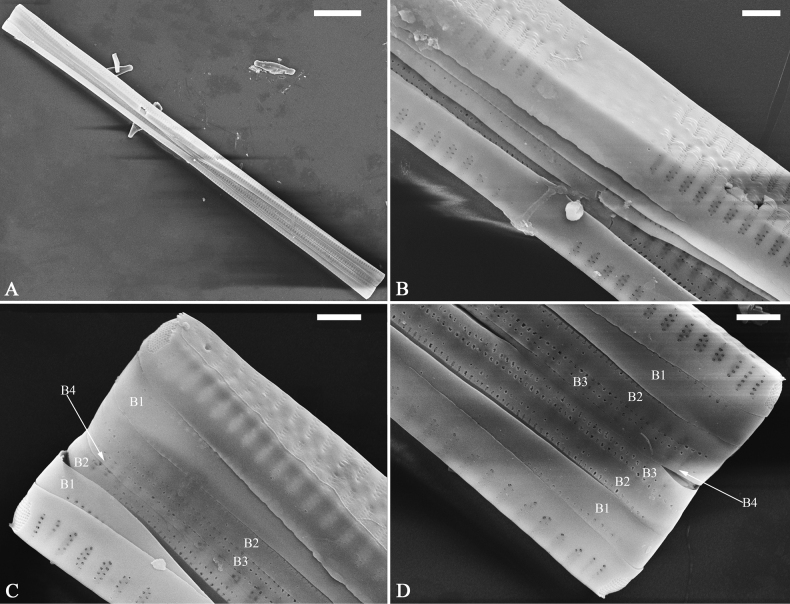
*Ulnariajishou-biseriata* sp. nov., girdle view, SEM**A** a frustule **B–D** details from **A**, showing a 4:3 configuration of girdle bands in a dividing cell. Scale bars: 20 μm (**A**); 2 μm (**B–D**).

**Figure 23.**
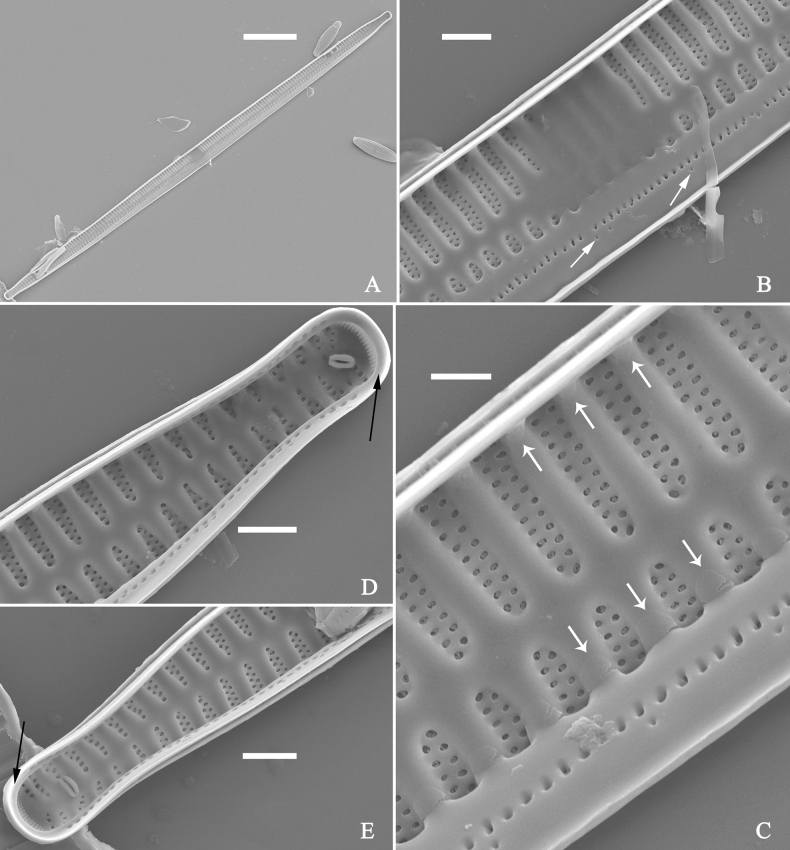
*Ulnariajishou-biseriata* sp. nov., internal view, SEM**A** a valve with valvocopula **B, C** middle details from **A**, note serrated projections over each virga (**C**, arrows) **D, E** two apical details from **A**, note unornamented valvocopula at both apices (arrow respectively). Scale bars: 20 μm (**A**); 2 μm (**B, D, E**); 1 μm (**C**).

**Figure 24.**
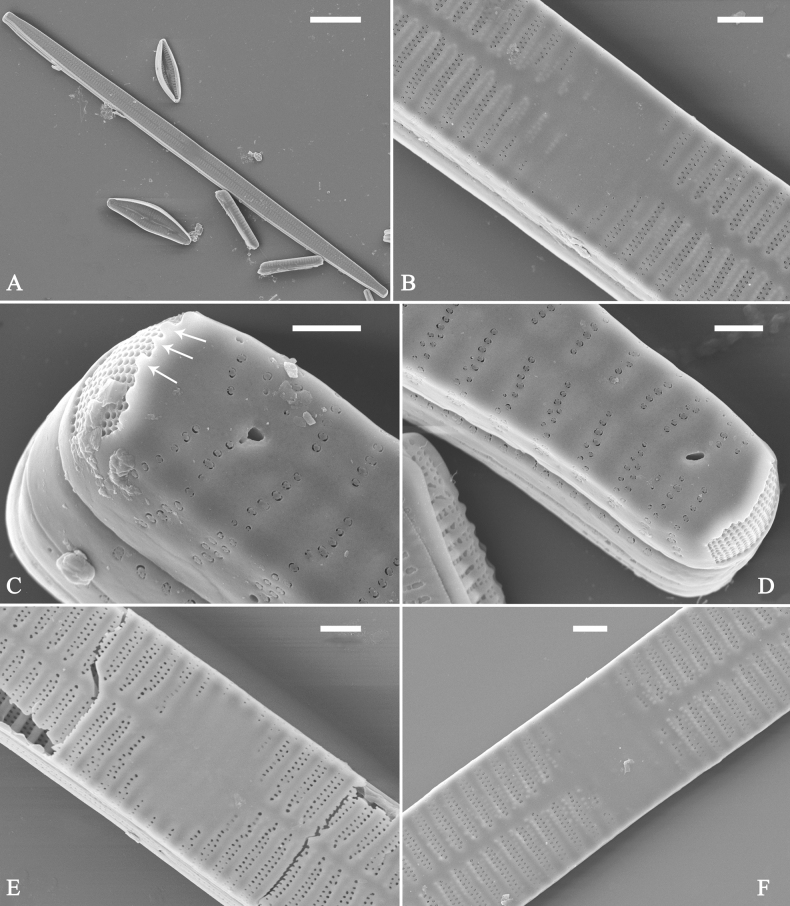
*Ulnariajishou-biseriata* sp. nov., external view, SEM**A** a frustule in valve view **B** middle detail from **A**, showing central area flanked by a few marginal striae only on one side **C, D** two apical details from **A**, note a few serrated projections protruding over the ocellulimbus (**C**, three arrows) **E** a middle part showing the central area flanked by shortened striae on both sides **F** a middle part showing a rectangular central area. Scale bars: 20 μm (**A**); 2 μm (**B, E, F**); 1 μm (**C, D**).

**Figure 25.**
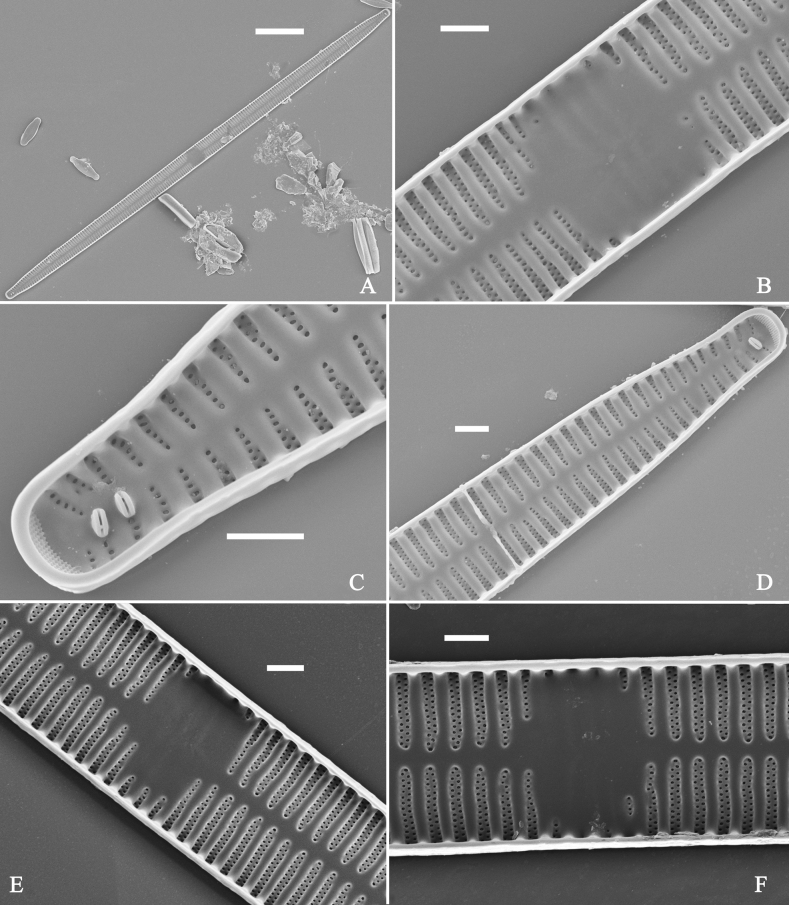
*Ulnariajishou-biseriata* sp. nov., internal view, SEM**A** a complete valve **B** middle detail from **A**, note the rectangular central area **C** apical detail from **A**, note two rimoportulae present **D** other apical detail from **A**, note only one rimoportula present **E, F** two middle parts, note the variable central areas. Scale bars: 20 μm (**A**); 2 μm (**B–F**).

Figs 19 , 20 , 21 , 22 , 23 , 24 , 25

##### Holotype.

Slide JIUDIA202302, specimen circled on slide, illustrated as Fig. 20D.

##### Registration.

PhycoBank http://phycobank.org/103808.

##### Type locality.

China. Hunan province: Jishou City, Lianaiqiao, Donghe River (28°18'51.3"N, 109°43'41.6"E, 200 m a.s.l.), collected by Bing Liu, December 9, 2016.

##### Description.

***LM*** (Figs 19, 20). Frustules rectangular in girdle view (Figs 19A, B, 20A), lanceolate in valve view (Fig. 19C, D). Chloroplasts two long plates per cell, valve-appressed (Fig. 19A), covering large portion of valve face in valve view (Fig. 19C, D). Valves lanceolate with prolonged, rostrate poles (Fig. 20B–J, see also Figs 23A, 24A, 25A), dimensions (n = 38): length 139–200 μm, width 6–8 μm at centre. Valve margins parallel near central area, gradually tapering towards rostrate poles (Fig. 20B–J). Sternum central, distinct, narrow, regular. Central area having three arrangements: extending towards both margins forming square to rectangular (Fig. 20B, J) or round hyaline space (Fig. 20C–E); extending to one margin with other margin flanked by few short striae (Fig. 20G–I); both margins bordered with few short striae (Fig. 20F). Ghost striae sometimes present (e.g., Fig. 20C, I) in central area. Striae parallel, radiate only at poles, and mostly opposite one another across sternum. Stria density 10–12 (often 11) in 10 μm.

***SEM*** (Figs 21–25). In some dividing cells, both epivalve and hypovalve associated with valvocopula and two copulae, forming 3:3 configuration of girdle bands (Fig. 21A–F, bands labelled B1–B3, B1 = valvocopula); sometimes epivalve associated with valvocopula and three copulae, whereas hypovalve associated with valvocopula and two copulae, forming 4:3 configuration (Fig. 22A–D). Copulae sometimes bearing two rows of poroids near poles (Fig. 21C–F). Valvocopula a closed hoop, same shape as valve outline, closely attached to mantle interior, surrounding the valve internal margin (Fig. 23A); bearing a mostly continuous row of poroids dividing pars interior from pars exterior located at midline (Fig. 23B–E), some isolated poroids visible in pars exterior (Fig. 23B, two arrows). At the advalvar edge, valvocopula produces a row of serrated projections, each corresponding to a virga internally (Fig. 23C, arrows). Valvocopula lacking ornamentation at both poles (Fig. 23D, E, black arrow respectively). Striae continuing onto mantle, absent from centre (Fig. 24B). Valve face and mantle intersecting almost at a right angle (Fig. 24B–F). Valve characterized by relatively wide virgae, interconnected with thin viminules, and closing plates with few struts fixing them onto each areolar wall (Fig. 24C, D). Valve has two types of mixed striae: one composed of a biseriate main part and a uniseriate minor part near sternum (usually 1 or 2 areolae), which are distributed on most of the valve face except each apex, and the other composed of a uniseriate main part and a biseriate minor part, which are only present near each apex (Figs 24C, D, 25B–F). Ocellulimbus composed of ca. 18 pervalvar and 10 transverse rows of porelli (both pervalvar and transverse rows unequal in length). A few serrated apical outgrowths protruding over each ocellulimbus (Fig. 24C, three arrows). Internally, virgae transversely extending towards mantle from sternum. Striae situated almost opposite each other across sternum and becoming uniseriate near sternum (Fig. 25B–F). Central area with three arrangements: extending to one margin, bordering with several short striae (Figs 24B, external, 25E, internal); bordering both margins with several short striae (Figs 24E, external, 25F, internal); or extending to both margins forming transapically rectangular fascia (Figs 24F, external, 25B, internal). One rimoportula at each pole (Figs 23D, E, 24C, D, 25D), occasionally two produced at an apex (Fig. 25C). External opening of rimoportula expressed as a simple hole with different shapes (Fig. 24C, D); internally bilabiate, situated close to sternum, aligned with striae (Fig. 25C, D).

##### Etymology.

The epithet *jishou-biseriata* is formed from the city name Jishou and the term biseriate to reflect its type locality (Jishou) and its mostly biseriate striae.

##### Ecology and distribution.

The sampling site is close to Jishou City and many anthropogenic influences affect the environment and hence the diatoms. The diatom samples were scraped off of the stone surfaces. The following environmental parameters were measured in the field: Conductivity was 202.3 ± 1.2 μS∙cm^-1^, pH was 8.5 ± 0.1, and water temperature was 13.2 ± 0.3 °C. So far, its distribution is known only from the type locality. To sum up, *U.jishou-biseriata* lives on the stone surfaces of a mountainous river running through a small city.

##### Discussion.

*Ulnariajishou-biseriata* is characterized by its lanceolate valve outline, mostly biseriate striae, and very variable central areas. With respect to the valve outline, it is similar in some ways to *U.ulnabiseriata*, but the former has smaller and very variable central areas whereas the latter’s central areas are always a rectangular fascia (see Liu et al. 2017, p 249, figs 30–35).

#### ﻿ Ulnaria pandurata-biseriata

Taxon classificationPlantaeLicmophoralesUlnariaceae

Bing Liu sp. nov.

DC66DB16-3C10-5B26-8661-C5390F0D8CA0

**Figure 26.**
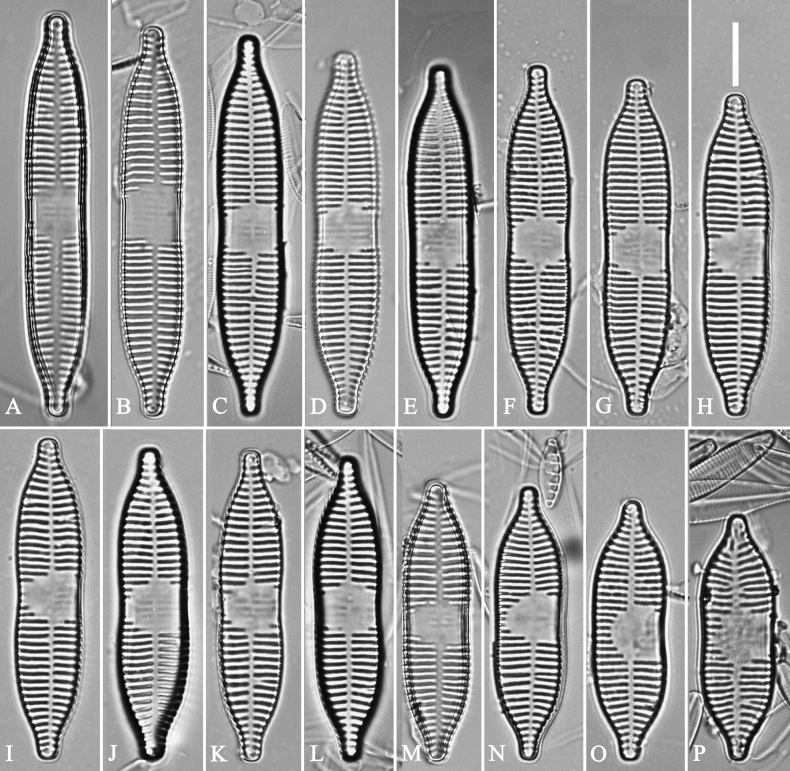
*Ulnariapandurata-biseriata* sp. nov., ×1000, LM**A–P** 16 valves exhibiting a size diminution series, note the panduriform valve outline and variable central areas **C** micrograph of holotype specimen. Scale bar: 10 μm.

**Figure 27.**
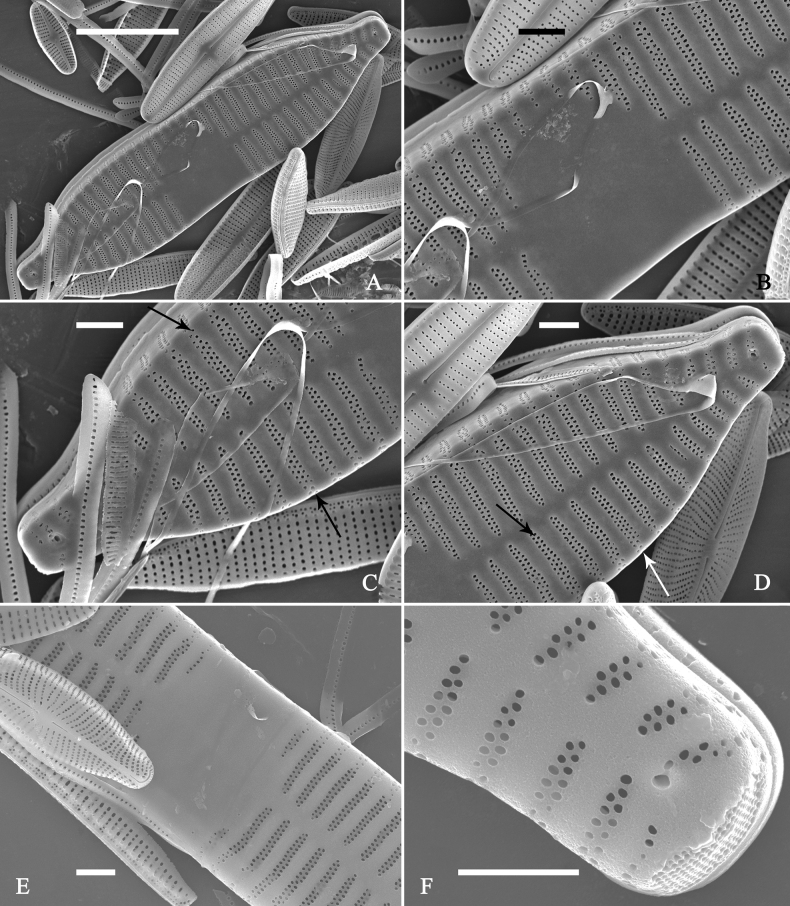
*Ulnariapandurata-biseriata* sp. nov., external view, SEM**A** a frustule in valve view **B** middle part detail from **A**, showing the central area with a few shortened marginal striae on one side **C, D** two apical details from **A**, note some partially triseriate striae (arrows) **E** another middle part showing the fascia-shaped central area **F** another apex, note a few serrated projections protruding over ocellulimbus. Scale bars: 10 μm (**A**); 2 μm (**B–F**).

**Figure 28.**
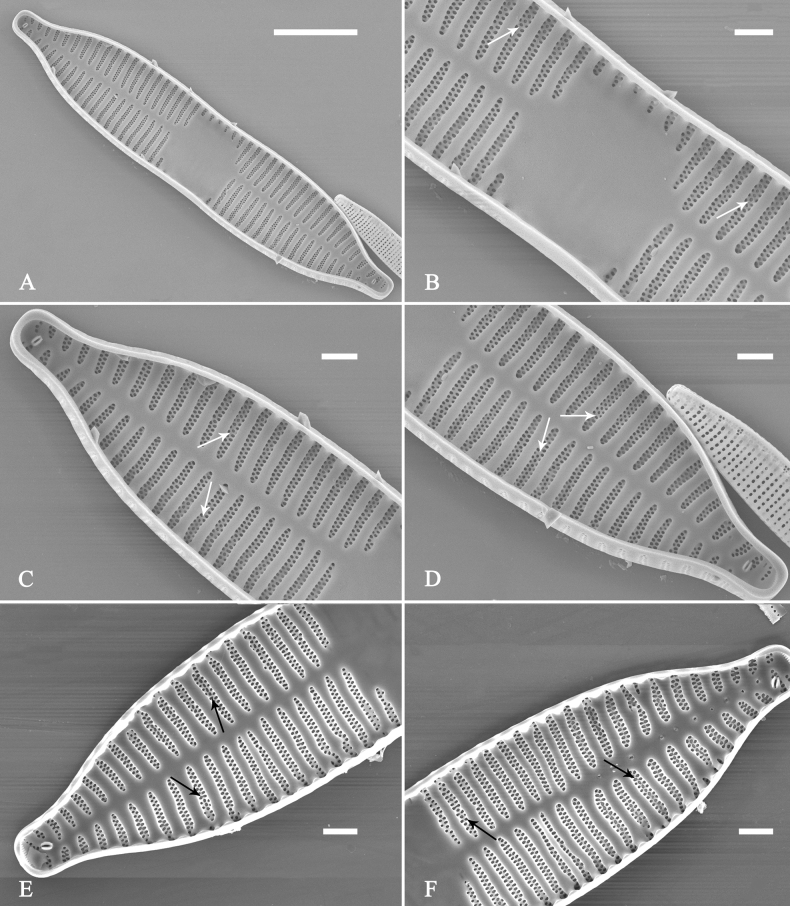
*Ulnariapandurata-biseriata* sp. nov., internal view, SEM**A** a complete valve **B–D** details from **A**, note some partially triseriate striae (arrows) **E, F** other apical details, note partially triseriate striae (arrows). Scale bars: 10 μm (**A**); 2 μm (**B–F**).

**Figure 29.**
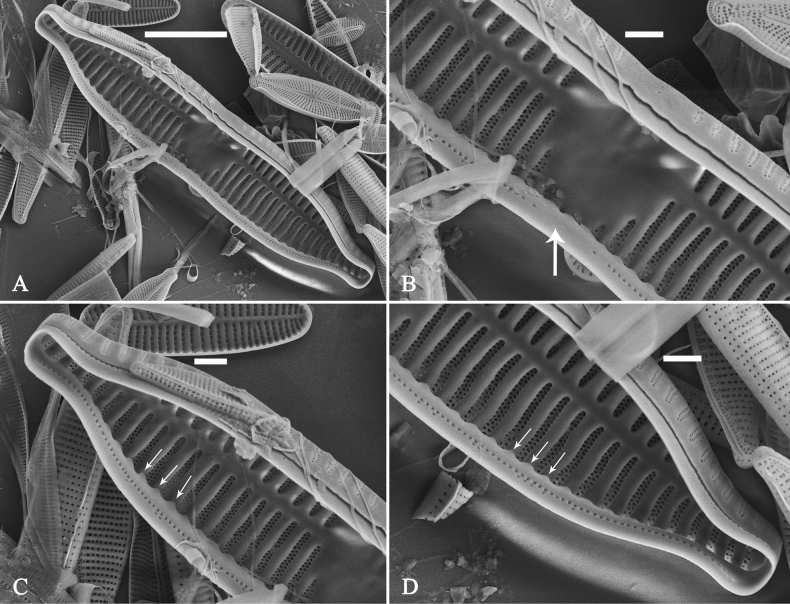
*Ulnariapandurata-biseriata* sp. nov., internal view, SEM**A** a valve with a valvocopula **B** middle detail from **A**, note the unornamented middle portion of valvocopula (arrow) **C, D** apical details from **A**, note serrated projections over each virga (arrows) and valvocopula without ornamentation at each apex. Scale bars: 10 μm (**A**); 2 μm (**B–D**).

Figs 26 , 27 , 28 , 29

##### Holotype.

Slide JIUDIA202303, specimen circled on slide, illustrated as Fig. 26C.

##### Registration.

PhycoBank http://phycobank.org/103809

##### Type locality.

China. Hunan province: Zhangjiajie National Forestry Park, Jinbian stream, at Shuirao Simen (29°20'36"N, 110°28'13"E, 467 m a.s.l.), collected by Bing Liu, December 29, 2015.

##### Description.

***LM*** (Fig. 26). Valves panduriform with slightly constricted middle part and rostrate poles (Fig. 26A–P, see also Figs 27A, 28A, 29A). Valve dimensions (n = 36): length 37–60 μm, width 7–9.5 μm at centre, 8–10 μm at widest part. Sternum distinct, extending length of valve. Central area has two arrangements: an asymmetric hyaline region extending to both margins (Fig. 26A–M) or a hyaline area which extends to one margin with the other side bordered with a few short striae (Fig. 26N–P). Ghost striae are sometimes present (e.g., Fig. 26D, K) in the central area. Striae parallel, radiate only approaching each apex, and mostly opposite one another across sternum. Striae density 9–11 (often 10) in 10 μm.

***SEM*** (Figs 27–29). Valve characterized by relatively wide virgae, interconnected with thin viminules, areolar closing plates having a few struts fixing them onto the areolar wall (Figs 27A–F, 28A–F, 29A–D). Valve with two types of mixed striae: one composed of a biseriate main part and a uniseriate minor part near sternum (usually 1 or 2 areolae), which describe most striae of valve (Figs 27A–F, 28A–F), and the other composed of a biseriate part, a triseriate part, and a uniseriate part near sternum (usually 1 or 2 areolae) (Figs 27C, D, 28B–F, two arrows, respectively). One rimoportula present at each pole, externally expressed as a simple hole (Fig. 27C, D, F), internally bilabiate, situated close to sternum (Fig. 28C–F). Ocellulimbus composed of ca.19 pervalvar and 9 transverse rows of porelli. A few serrated apical outgrowths protruding over the ocellulimbus (Fig. 27F). Valvocopula is a closed hoop, attached to the mantle interior, surrounding internal valve margin (Fig. 29A). Each valvocopula bears a mostly continuous row of poroids dividing the pars interior from pars exterior, located at the midline (Fig. 29A–D); lacking ornamentation at either apex (Fig. 29C, D). On its advalvar edge, valvocopula bears a row of serrated projections, each corresponding internally to a virga (Fig. 29C, D, three arrows respectively).

##### Etymology.

The epithet *pandurata-biseriata* is a combination of the terms pandurate and biseriate to reflect the valvar panduriform outline of the valve and its mostly biseriate striae.

##### Ecology and distribution.

The sampling site is situated close to the headwaters of Jinbian stream, which originates in the Zhangjiajie National Forestry Park. The diatom samples were scraped off of stone surfaces. The following environmental parameters were measured in the field: Conductivity was 102.7 ± 0.8 μS∙cm^-1^, pH was 8.5 ± 0.7 and water temperature was 8.7 ± 0.2 °C. So far, its distribution is known only from the type locality. To sum up, *U.pandurata-biseriata* lives on the stone surfaces of the headwaters of a mountainous stream.

##### Discussion.

*Ulnariapandurata-biseriata* is characterized by its panduriform valve outline, mostly biseriate striae, variable central areas, and smaller valves. Similar taxa include *U.goulardii* D.M. Williams, Potapova & C.E. Wetzel (see Williams 1986, p. 141, figs 27–36) and *U.sangzhi-biseriata* sp. nov (see below). All possess mostly biseriate striae, but they can be distinguished by the panduriform valve outline of *U.pandurata-biseriata* from the two latter’s lanceolate valve outline (for *U.goulardii*, see Williams 1986, p. 141, figs 27–36, as *Synedragoulardii*).

#### ﻿ Ulnaria sangzhi-biseriata

Taxon classificationPlantaeLicmophoralesUlnariaceae

Bing Liu sp. nov.

9F2CEEEE-746C-5AAD-8A24-70264C149BBA

**Figure 30.**
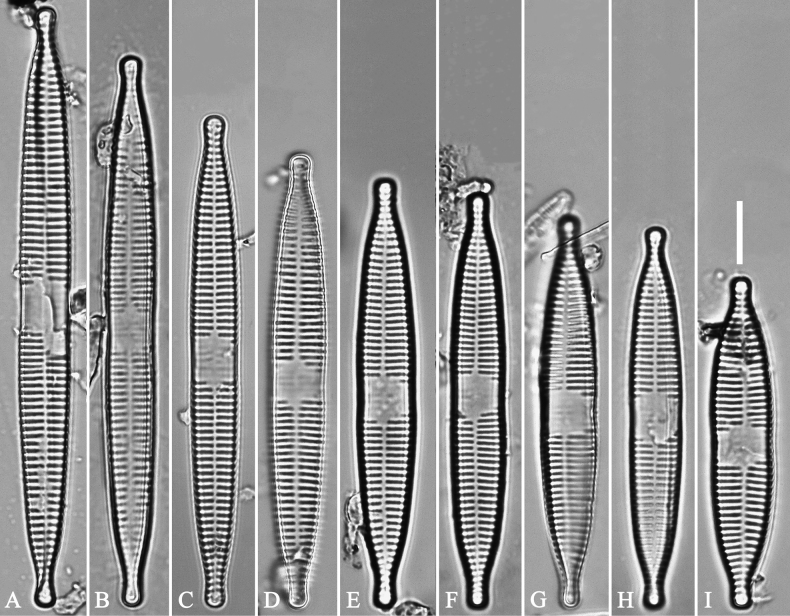
*Ulnariasangzhi-biseriata* sp. nov., ×1000, LM**A–I** nine valves exhibiting a size diminution series, note linear-lanceolate valve outline, rectangular central area and capitate to sub-capitate apices **C** micrograph of holotype specimen. Scale bar: 10 μm.

**Figure 31.**
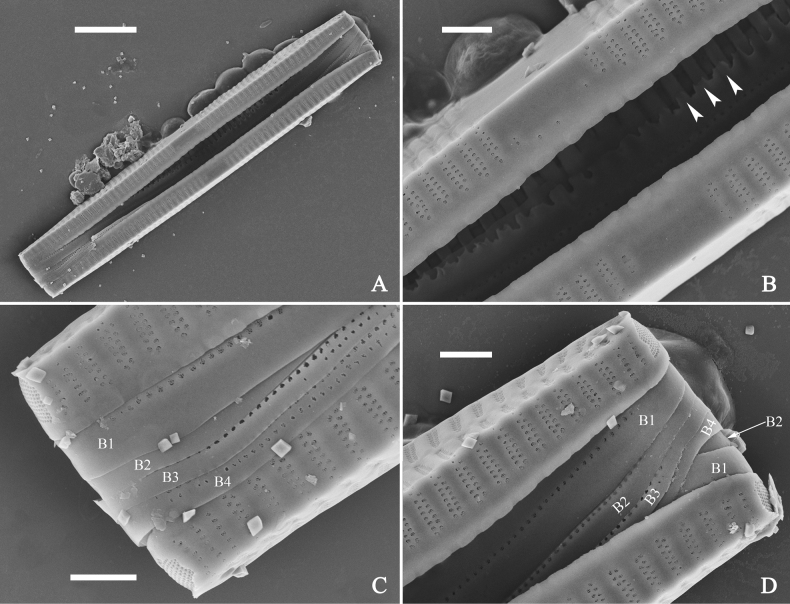
*Ulnariasangzhi-biseriata* sp. nov., girdle view, SEM**A** a partly collapsed frustule **B** middle part from **A**, note variable central mantles and serrated projections of valvocopula (three arrowheads) **C, D** two apical details from **A**, showing four girdle bands associating the epivalve (labelled B1–B4). Scale bars: 10 μm (**A**); 2 μm (**B–D**).

**Figure 32.**
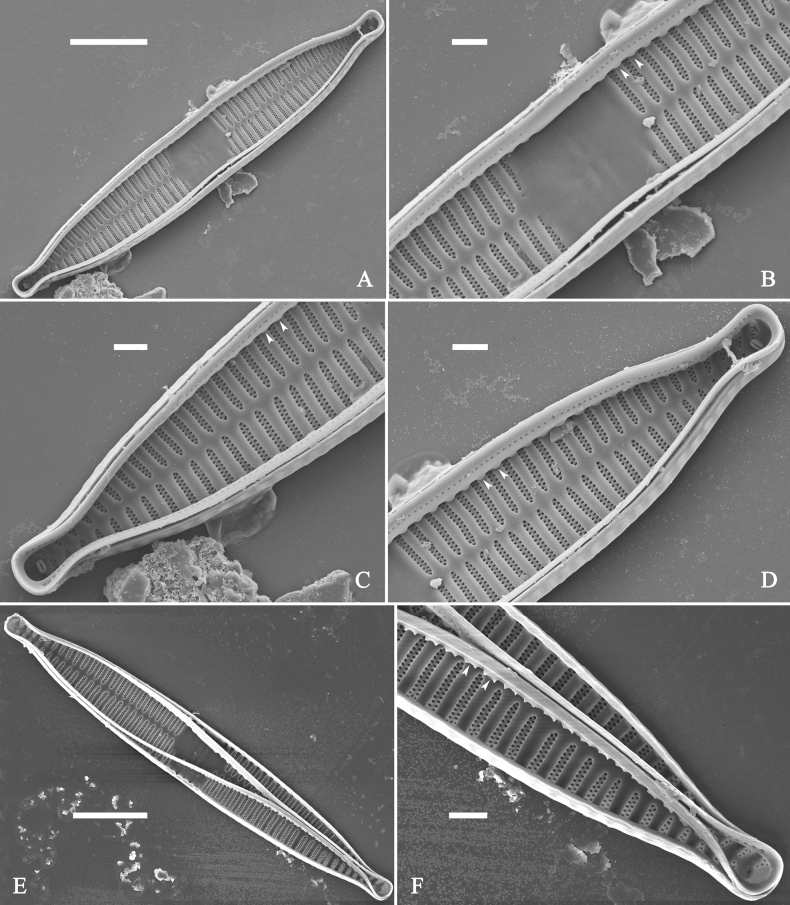
*Ulnariasangzhi-biseriata* sp. nov., internal view, SEM**A** a valve with a valvocopula **B** middle part from **A**, note the serrated projections (two arrowheads) **C, D** two apical details from **A**, note the closed nature of valvocopula and serrated projections (arrowheads) **E** another valve with a valvocopula **F** apical detail from **E**, note the serrated projections (two arrowheads). Scale bars: 10 μm (**A, E**); 2 μm (**B–D, F**).

**Figure 33.**
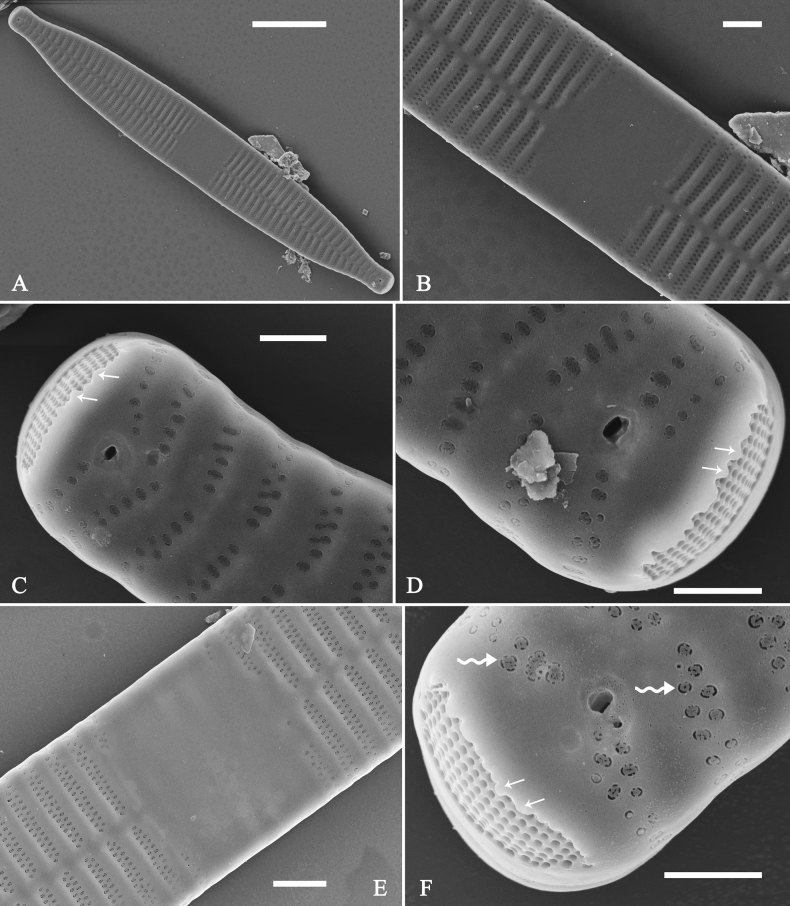
*Ulnariasangzhi-biseriata* sp. nov., external view, SEM**A** a complete valve **B** middle detail from **A**, note rectangular central area **C, D** two apical details from **A**, note a few serrated projections protruding over the ocellulimbus (arrows) **E** another middle part showing rectangular central area with ghost striae **F** another apical detail, note a few serrated projections (two arrows) and the closing plates (two curved arrows). Scale bars: 10 μm (**A**); 2 μm (**B, E**); 1 μm (**C, D, F**).

**Figure 34.**
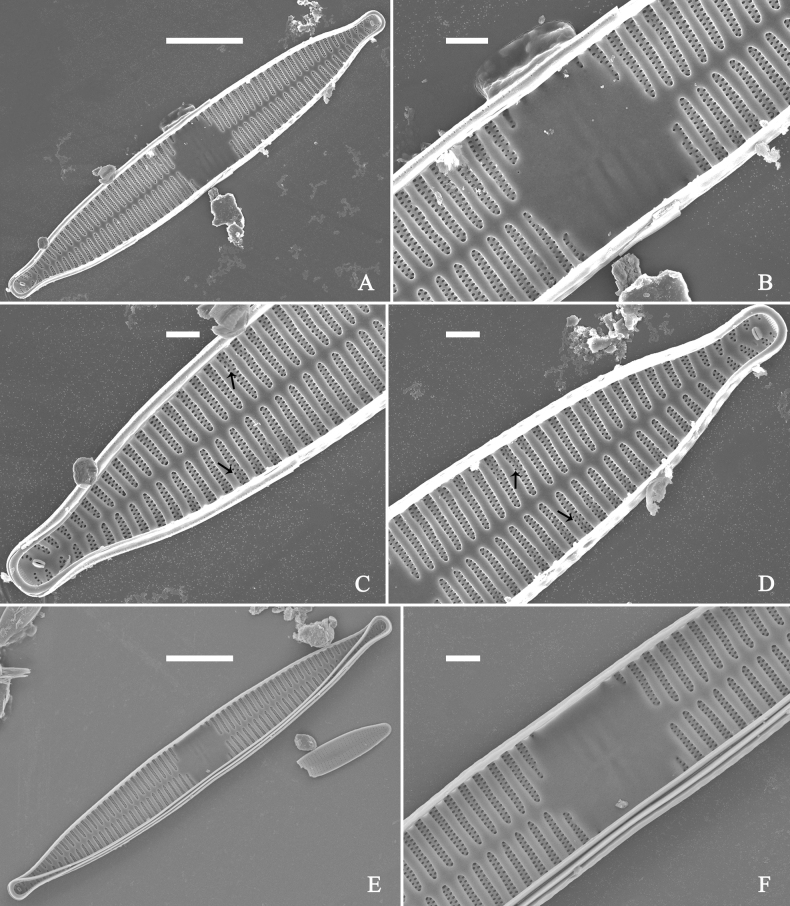
*Ulnariasangzhi-biseriata* sp. nov., internal view, SEM**A** a complete valve **B** middle detail from **A**, showing the central area **C, D** two apical details from **A**, note part triseriate striae (arrows) **E** another complete valve **F** middle detail from **E**. Scale bars: 10 μm (**A, E**); 2 μm (**B–D, F**).

Figs 30 , 31 , 32 , 33 , 34

##### Holotype.

Slide JIUDIA202304, specimen circled on slide, illustrated as Fig. 30C.

##### Registration.

PhycoBank http://phycobank.org/103810.

##### Type locality.

China. Hunan province: Sangzhi County, Wudaoshui Town, Jinlong power station, Li River (29°43′7.1″N, 109°54′50.9″E, 398 m a.s.l.), collected by Bing Liu, September 30, 2015.

##### Description.

***LM*** (Fig. 30). Valves linear-lanceolate with central margins sometimes very slightly constricted and capitate to sub-capitate apices (Fig. 30A–I, see also Figs 32A, E, 33A, 34A, E). Valve dimensions (n = 22): length 49–91 μm, width 6.5–8.2 μm at centre. Sternum discernible, extending length of valve. Central area rectangular or square. Ghost striae present in central area (e.g., Fig. 30B–D). Striae parallel, radiate only at poles, and mostly opposite each other across sternum. Stria density 10–12 (often 11) in 10 μm.

***SEM*** (Figs 31–34). Frustule rectangular in girdle view (Fig. 31A). Epivalve associated with valvocopula and three copulae (Fig. 31C, D, labelled B1–B4, B1 = valvocopula). Valvocopula is a closed hoop, attached to the mantle interior, surrounding the valve internal margin (Fig. 32A, E). The valvocopula has a mostly continuous row of poroids dividing pars interior from pars exterior, located at the midline (Fig. 26B–D). On its advalvar edge there is a row of serrated projections, each corresponding internally to a virga (Figs 31B, 32B–D, F, two arrowheads respectively), ornamentation is lacking at either pole (Fig. 32C, D, F). Valve face and mantle intersect almost at right angle (Fig. 33A–F). Central hyaline area rectangular or square (Figs 33B, E, 34B, F). Valve characterized by a series of relatively wide virgae, interconnected with thin viminules and closing plates affixed with a few struts to the areolar wall (Figs 32A–F, 33B–F, closing plate see Fig. 33F, two wavy arrows). Valve has two types of mixed striae: most striae of the valve are composed of a biseriate main part and a uniseriate minor part near the sternum (usually 1 or 2 areolae, Figs 33B–F, 34B–D, F), and the rest are composed of a biseriate part, a triseriate part, and a uniseriate part near sternum (usually 1 or 2 areolae) (Fig. 34C, D, two arrows respectively). Ocellulimbus composed of ca. 18 pervalvar and 7 transverse rows of porelli. A few serrated apical outgrowths protruding over each ocellulimbus (Fig. 33C, D, F, two arrows respectively). Striae continue onto mantle, absent in the centre (Fig. 31B). One rimoportula at each pole (Figs 33C, D, F, 34C, D), externally expressed as a simple hole in different shapes (Fig. 33C, D, F), internally bilabiate, situated close to sternum (Fig. 34C, D).

##### Etymology.

The epithet *sangzhi-biseriata* is a combination of Sangzi and the term biseriate to reflect its type locality (Sangzi) and its mostly biseriate striae.

##### Ecology and distribution.

The sampling site is near Wudaoshui Town, and some human activities may have affected the environment and hence the diatoms. The diatom samples were scraped off of the stone surfaces. The following environmental parameters were measured in the field: Conductivity was 219.3 ± 1.2 μS∙cm^-1^, pH was 8.5 ± 0.2 and water temperature was 17.9 ± 0.3 °C. To sum up, *U.sangzhi-biseriata* lives on the stone surfaces of a mountainous river flowing away from the Town. So far, its distribution is known only from the type locality.

##### Discussion.

*Ulnariasangzhi-biseriata* is characterized by its linear-lanceolate valve outline, mostly biseriate striae, rectangular or square central area, and capitate to sub-capitate apices. It is similar to *U.goulardii* which has a more constricted valve central margin. Moreover, the former has capitate to sub-capitate apices whereas *U.goulardii* has rostrate apices (see Williams 1986, p. 141, figs 27–36, as *Synedragoulardii*).

#### ﻿ Ulnaria wuling-biseriata

Taxon classificationPlantaeLicmophoralesUlnariaceae

Bing Liu sp. nov.

96A4A27C-9D80-52D2-B6EB-4C0B0A645655

**Figure 35.**
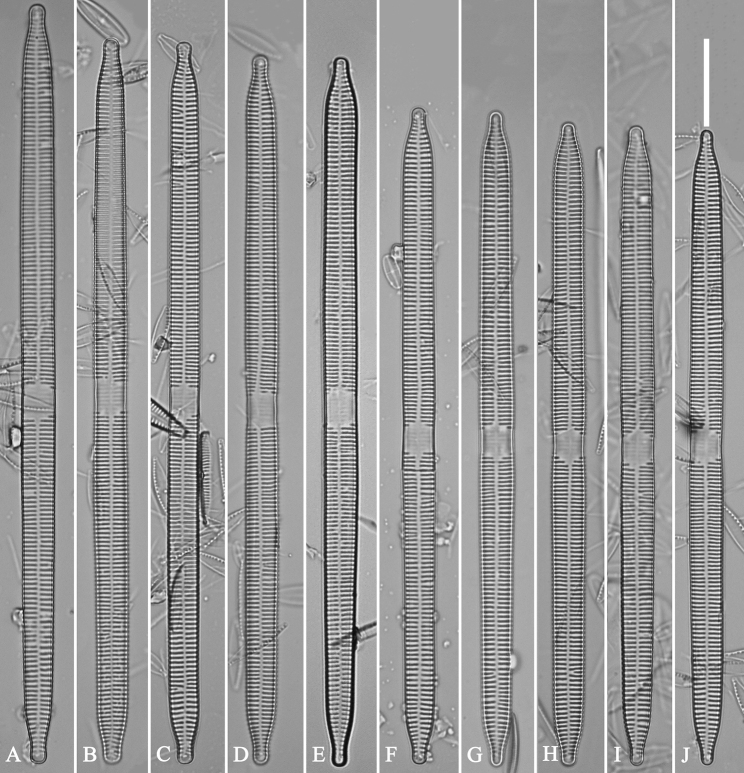
*Ulnariawuling-biseriata*, sp. nov., ×630, LM**A–J** ten valves exhibiting a size diminution series, note linear valve outlines, rostrate apices, and rectangular hyaline central area **A** micrograph of holotype specimen. Scale bar: 20 μm.

**Figure 36.**
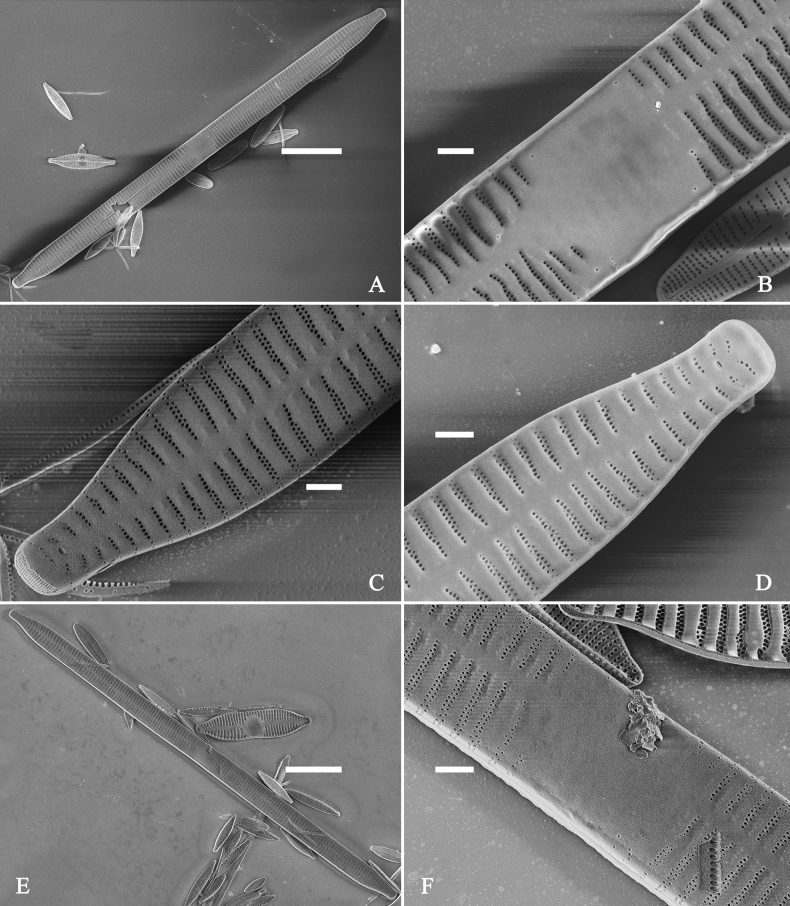
*Ulnariawuling-biseriata*, sp. nov., external view, SEM**A** a complete valve **B** middle detail from **A**, showing the rectangular central area **C, D** two apical details of **A**, note some uniseriate striae at the apices **E** another complete valve **F** middle detail from **E**, showing a trapezoidal central area. Scale bars: 20 μm (**A, E**); 2 μm (**B–D, F**).

**Figure 37.**
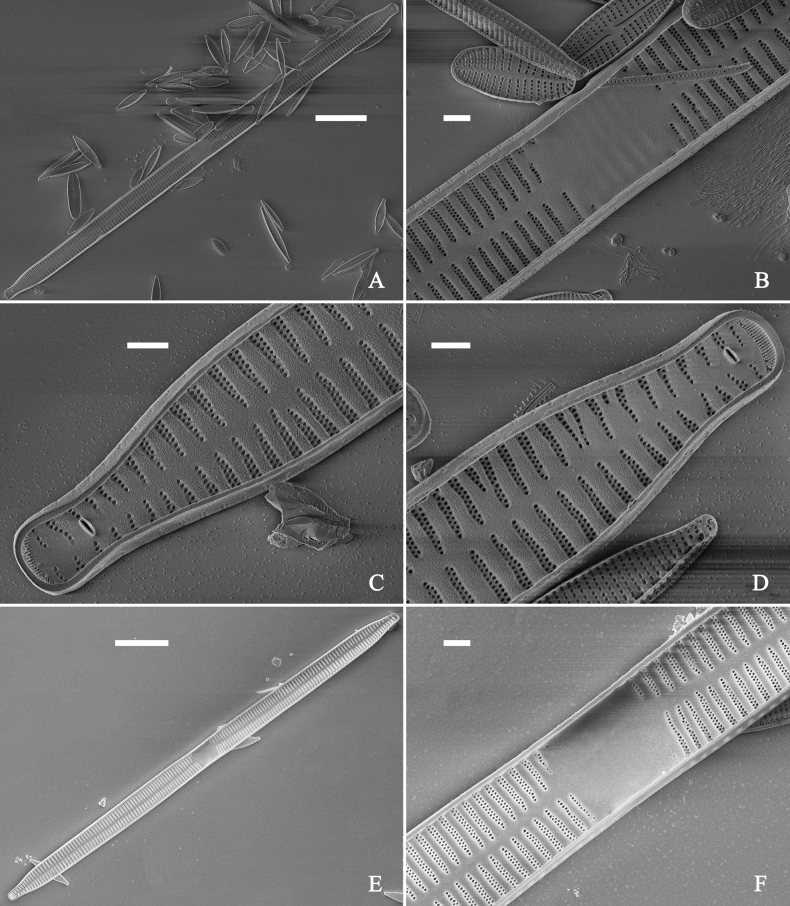
*Ulnariawuling-biseriata*, sp. nov., internal view, SEM**A** a complete valve **B** middle detail from **A**, showing the rectangular central area **C, D** two apical details from **A**. **E** Another complete valve **F** middle detail from **E** showing the trapezoidal central area. Scale bars: 20 μm (**A, E**); 2 μm (**B–D, F**).

**Figure 38.**
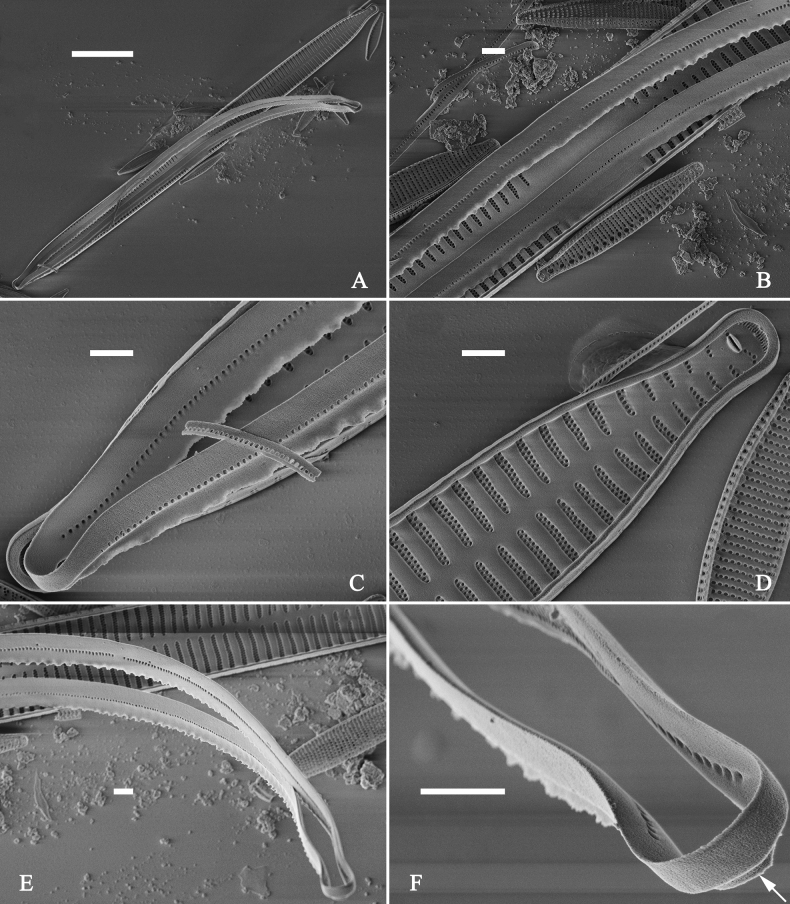
*Ulnariawuling-biseriata*, sp. nov., internal view, SEM**A** a valve with a valvocopula **B** middle detail from **A**. **C, D** two apical details of **A**, note the valvocopula unornamented at each apex **E, F** details of the valvocopula, note its closed nature and the shelf-like projection (**F**, arrow). Scale bars: 20 μm (**A**); 2 μm (**B–F**).

Figs 35 , 36 , 37 , 38

##### Holotype.

Slide JIUDIA202305, specimen circled on slide, illustrated as Fig. 35A.

##### Registration.

PhycoBank http://phycobank.org/103811

##### Type locality.

China. Hunan province: Zhangjiajie National Forestry Park, Jinbian stream, at Shuirao Simen (29°20'36"N, 110°28'13"E, 467 m a.s.l.), collected by Bing Liu, December 29, 2015.

##### Description.

***LM*** (Fig. 35). Valves linear with abruptly tapering rostrate to sub-capitate apices (Fig. 35A–J, see also Figs 36A, E, 37A, E). Valve dimensions (n = 15): length 160–200 μm, width 6.5–8 μm at centre. Sternum discernible, mostly regular, occasionally irregular (e.g., Fig. 35D), extending length of valve. Central area apically rectangular or trapezoid. Ghost striae sometimes present in central area (e.g., Fig. 35C, I). Striae parallel, radiate only at each pole, and mostly opposite across sternum. Stria density 10–11 in 10 μm.

***SEM*** (Figs 36–38). Valves mostly with mixed striae, each composed of a biseriate main part and a uniseriate minor part (including several areolae) near sternum (Figs 36B–D, F, 37B–D, F). But some uniseriate striae occurring near each apex (Fig. 36C, D). One rimoportula at each pole, externally expressed as a simple hole (Fig. 36C, D), internally bilabiate, situated close to sternum (Figs 37C, D, 38D). Ocellulimbus composed of ca. 24 pervalvar and 8 transverse rows of porelli. Valvocopula a closed hoop (Fig. 38A), with a mostly continuous row of poroids dividing pars interior from pars exterior, located at the midline (Fig. 38B, C, E). A row of serrated projections is located on its advalvar edge, each corresponding internally to a virga (Fig. 38B, C), unornamented at both poles (Fig. 38C, F).

##### Etymology.

The epithet "*wuling-biseriata*" is a combination of Wuling and the term biseriate to reflect its type locality (Wuling Mountains) and its mostly biseriate striae.

##### Ecology and distribution.

*Ulnariawuling-biseriata* was commonly found in the surface sediment collected in Jinbian stream with *U.pandurata-biseriata* and *U.ulnabiseriata*. Thus, *U.wuling-biseriata* lives on the stone surfaces of the headwaters of a mountainous stream. So far, its distribution is known only from the type locality.

##### Discussion.

*Ulnariawuling-biseriata* is characterized by its linear valve outline, mostly biseriate striae, rectangular or trapezoid central area, and rostrate to sub-capitate apices. The apices of Ulnariaulnavar.spathulifera Aboal sometimes have an inflation before tapering to form subrostrate to rostrate ends whereas *U.wuling-biseriata* does not have this inflation, i.e., its apex is not spatulate. Moreover, the valves of U.ulnavar.spathulifera are wider than the ones of *U.wuling-biseriata* (8–9 μm vs 6.5–8 μm) and the former has lower stria density than the latter (9–10 in 10 μm vs 10–11 in 10 μm) (see Morales et al. 2007, p. 34, as Synedraulnavar.spathulifera).

#### ﻿ Ulnaria blancoi

Taxon classificationPlantaeLicmophoralesUlnariaceae

Bing Liu sp. nov.

A3CC9D81-1635-55D8-8EF7-BCA7DDF68E4B

**Figure 39.**
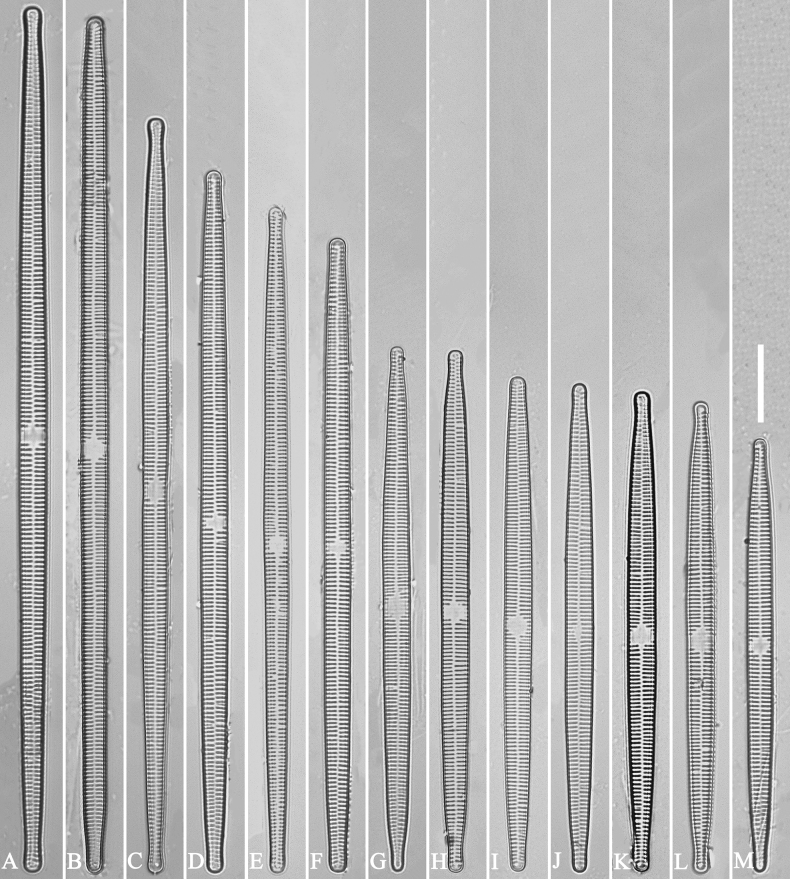
*Ulnariablancoi* sp. nov., ×630, LM**A–M** thirteen valves exhibiting a size diminution series, note the lanceolate valve outline and small variable central areas **A** micrograph of holotype specimen. Scale bar: 20 μm.

**Figure 40.**
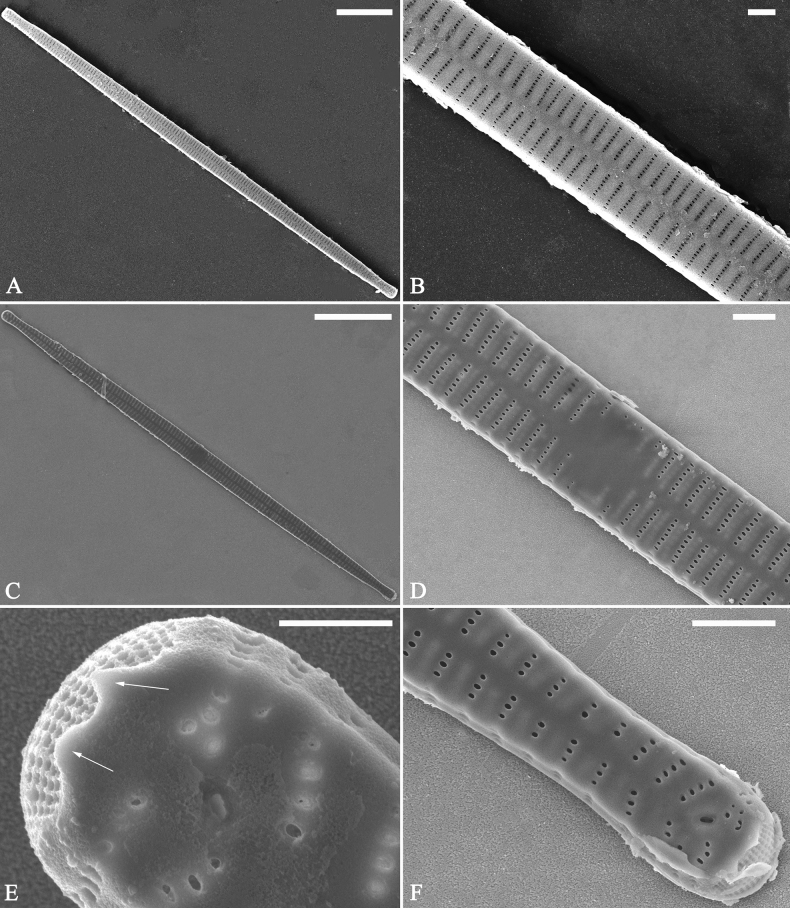
*Ulnariablancoi* sp. nov., external view, SEM**A, C** two complete valves, note central area present or absent **B** middle part from **A**, note central area completely absent **D** middle part from **C**, note small central area flanked by marginal short striae **E, F** two apical details, note two horn-like projections protruding over the ocellulimbus (**E**, two arrows). Scale bars: 20 μm (**A, C**); 2 μm (**B, D, F**); 1 μm (**E**).

**Figure 41.**
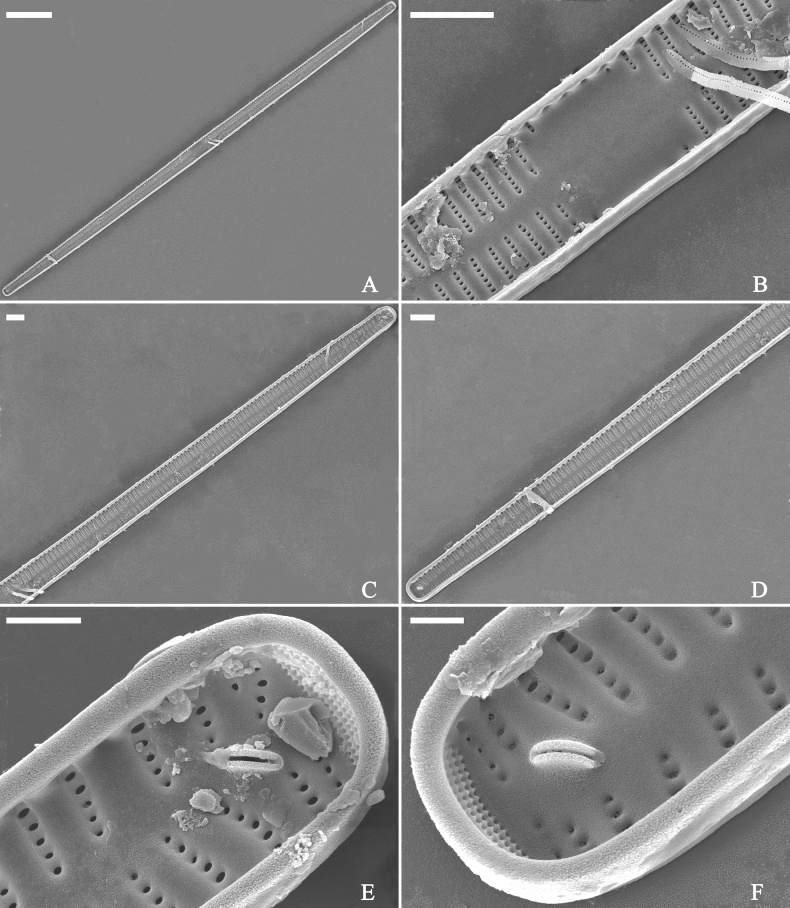
*Ulnariablancoi* sp. nov., internal view, SEM**A** a complete valve **B** middle part detail from **A**, note rectangular central area **C, D** two half parts from **A**. **E, F** Two apical details from **A**. Scale bars: 20 μm (**A**); 4 μm (**B–D**); 2 μm (**E, F**).

**Figure 42.**
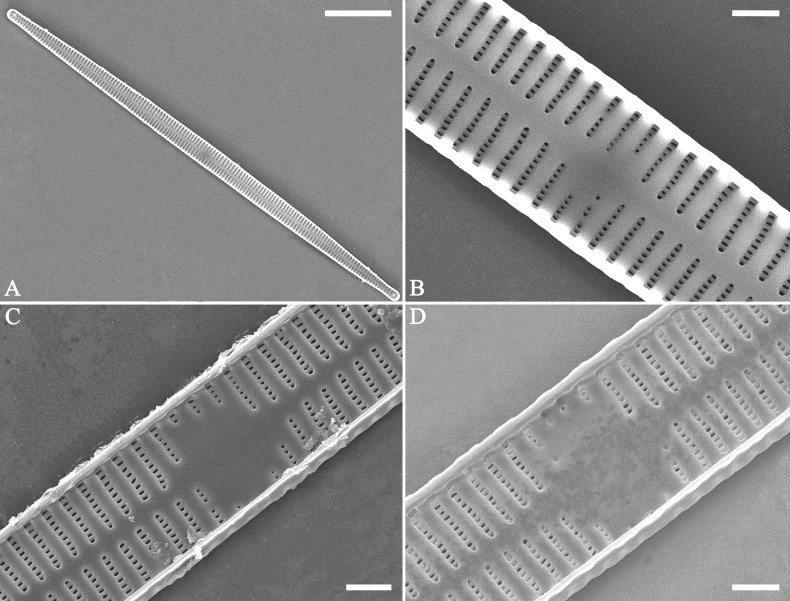
*Ulnariablancoi* sp. nov., internal view, SEM**A** a complete valve **B** middle part from **A**, note the very small central area **C, D** other valve middle part details, note variable central areas. Scale bars: 20 μm (**A**); 2 μm (**B–D**).

**Figure 43.**
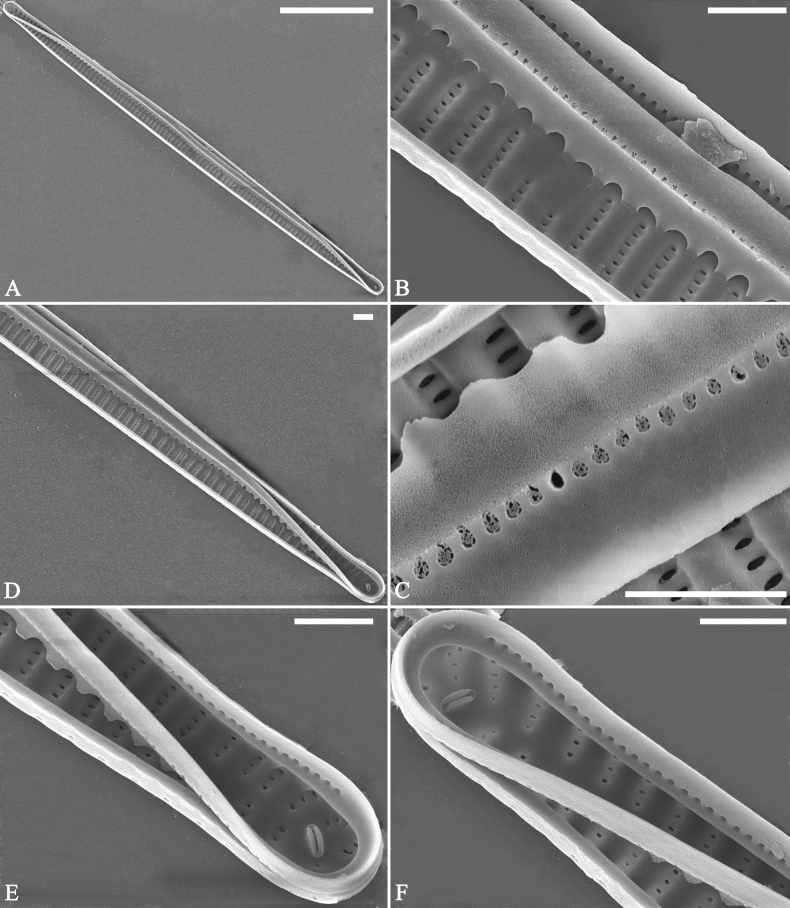
*Ulnariablancoi* sp. nov., internal view, SEM**A** a valve with valvocopula **B** middle part detail from **A**. **C** Detail of valvocopula, note closing plates **D–F** apical details from **A**. Scale bars: 20 μm (**A**); 2 μm (**B–F**).

Figs 39 , 40 , 41 , 42 , 43

##### Holotype.

Slide JIUDIA202306, specimen circled on slide, illustrated as Fig. 39A.

##### Registration.

PhycoBank http://phycobank.org/103812.

##### Type locality.

China. Qinghai province: Menyuan County, an unnamed river, at a sampling location named Kengtan (37°27'28"N, 101°23'15"E, 2940 m a.s.l.), collected by Bing Liu, July 18, 2019.

##### Description.

***LM*** (Fig. 39). Valves lanceolate with rostrate to sub-capitate apices (Fig. 39A–M, see also Figs 40A, C, 41A, 42A). Valve dimensions (n = 24): length 104–236 μm, width 4.6–6.8 μm at centre. Sternum distinct, extending length of valve. Central area very variable: hyaline region extending to both margins forming rectangular fascia (e.g., Fig. 39A, see also Fig. 41B). It can be circumscribed by both short marginal striae and isolated areolae (e.g., Fig. 39D–F, see also Fig. 40D), nearly absent (Fig. 39J, see also Fig. 42B) or completely lacking (see Fig. 40A, B). Striae parallel, radiate only approaching each apex, some opposite each other, and others alternate across the sternum. Stria density 10–13 in 10 μm, increasing near each apex.

***SEM*** (Figs 40–43). Valve characterized by a series of relatively wide virgae, interconnected with vimines and closing plates attached with a few struts to each areolar wall (Figs 40A–F, 41A–F, 42A–D, closing plates see Fig. 43C). Striae uniseriate. Each stria on both sides opposite or alternate, areolae gradually apically elongated from sternum to mantle (Figs 40B, D, 41B, 42B–D). Central area completely lacking (Fig. 40A, B), hyaline region circumscribed by both short marginal striae and isolated areolae (Figs 40C, 42B–D), or hyaline area extending to both margins (Fig. 41B). Ocellulimbus composed of ca. 16 pervalvar and 7 transverse rows of porelli. Two horn-like projections protruding over the ocellulimbus (Fig. 40E, two arrows). One rimoportula located at each pole, externally expressed as a simple hole (Fig. 40E, F), internally bilabiate, situated close to sternum at an angle (Fig. 41E, F). Valvocopula a closed hoop, surrounding the valve internal margin (Fig. 43A). Valvocopula bearing a mostly continuous row of poroids dividing pars interior from pars exterior, located at the midline (Fig. 43B–F), lacking ornamentation at both poles (Fig. 43E, F). On its advalvar edge, valvocopula has a row of serrated projections, each corresponding internally to a virga (Fig. 43B–E).

##### Etymology.

Named after the Spanish diatomist, Dr. Saúl Blanco.

##### Ecology and distribution.

The sampling site is located in the plateau which belongs to the highland continental climate zone. The diatom samples were scraped off of the stone surfaces. The following environmental parameters were measured in the field: Conductivity was 448.3 ± 0.5 μS∙cm^-1^, pH was 8.3 ± 0.1 and water temperature was 11.9 ± 0.5 °C. To sum up, *U.blancoi* lives on the stone surfaces of a plateau river. So far, its distribution is known only from the type locality.

##### Discussion.

*Ulnariablancoi* is characterized by its lanceolate valve outline, uniseriate striae, very variable central areas, and rostrate to sub-capitate apices. It differs from *U.vitrea* (Kützing) E. Reichardt by its much longer valves (104–236 μm vs 90–120 μm) and its often-present central area whereas *U.vitrea* often lacks central area (see Williams 2020, p. 4). *Ulnariasplendens* (Kützing) D.M. Williams et Van de Vijver is wider (5–10 μm vs 4.6–6.8 μm) and its stria density is lower than that of *U.blancoi* (6–10 striae in 10 μm vs 10–13 striae in 10 μm). Moreover, *U.blancoi* often has sub-capitate apices whereas *U.splendens* has rostrate apices (see Williams and Van de Vijver 2021, p. 167, figs 1–10).

#### ﻿ Ulnaria menyuanensis

Taxon classificationPlantaeLicmophoralesUlnariaceae

Bing Liu sp. nov.

006A480B-45CD-5476-A234-68D0D837E060

**Figure 44.**
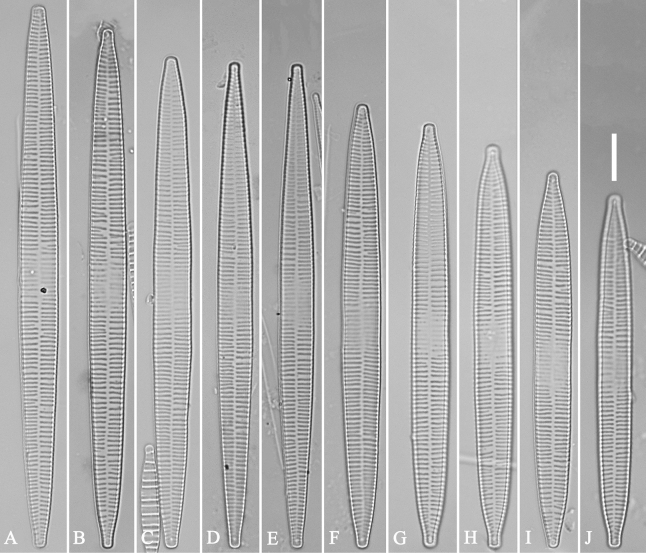
*Ulnariamenyuanensis* sp. nov., ×1000, LM**A–J** ten valves exhibiting a size diminution series, note the lanceolate valve outline and distinctive ghost striae in the middle regions of the valves **D** micrograph of holotype specimen. Scale bar: 10 μm.

**Figure 45.**
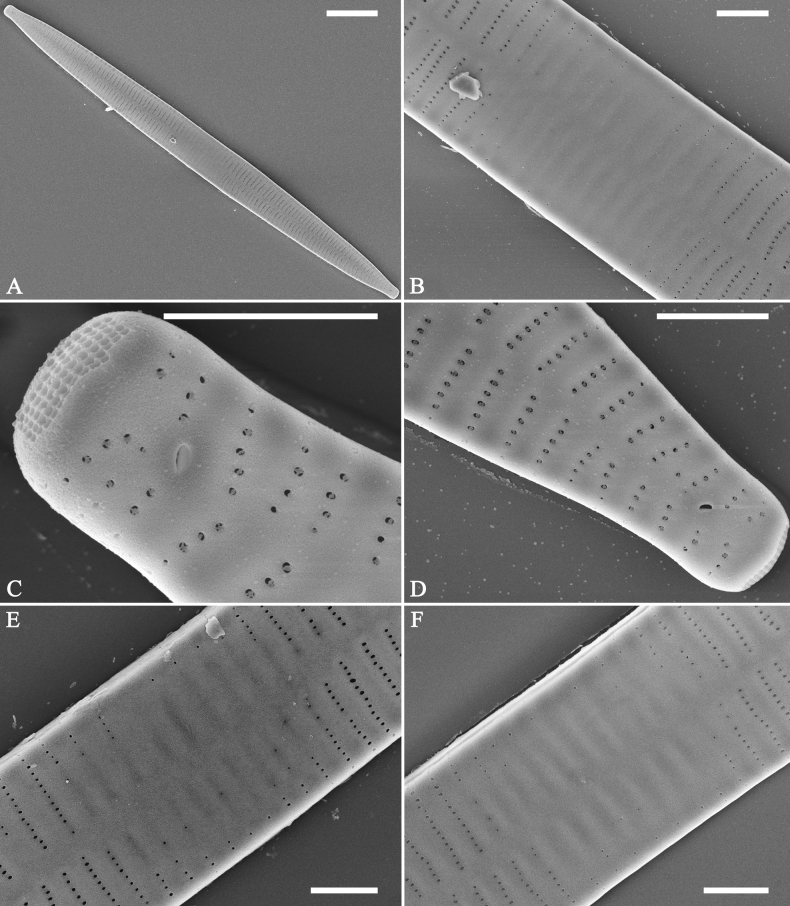
*Ulnariamenyuanensis* sp. nov., external view, SEM**A** a complete valve **B** middle part detail from **A**, note the shallow grooves and short striae at the central region **C, D** two apical details from **A**. **E, F** Other middle parts, note the shallow grooves and short striae. Scale bars: 10 μm (**A**); 2 μm (**B–F**).

**Figure 46.**
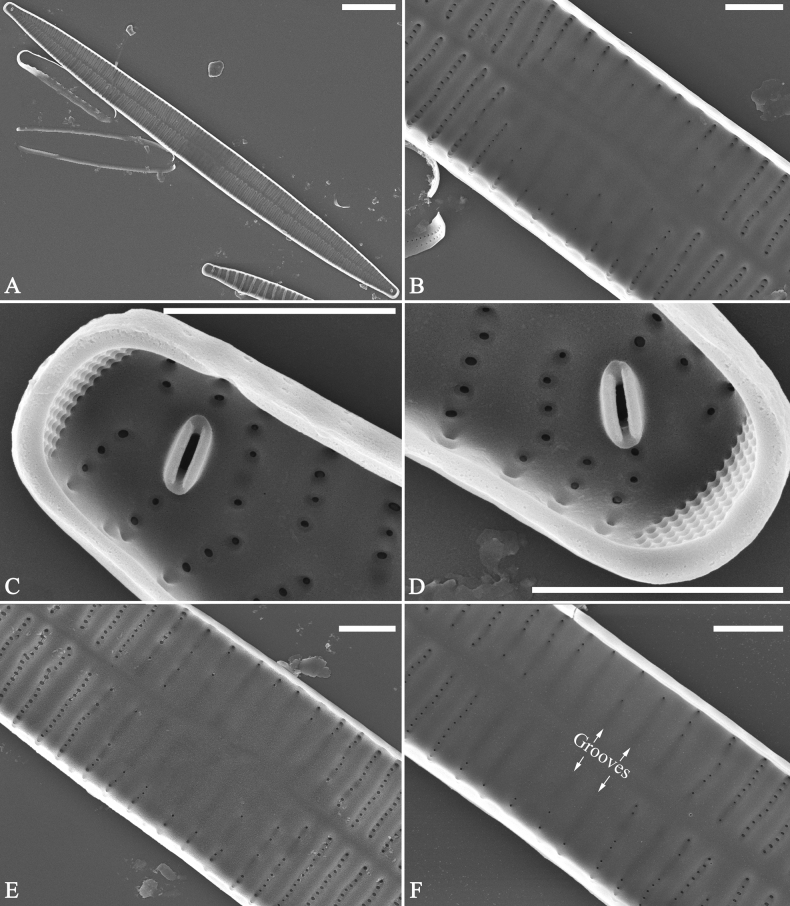
*Ulnariamenyuanensis* sp. nov., internal view, SEM**A** a complete valve **B** middle part from **A**, note shallow grooves and short striae **C, D** two apical details from **A**, note the bilabiate rimoportulae and apical pore fields **E, F** other middle parts, note shallow grooves and short striae. Scale bars: 10 μm (**A**); 2 μm (**B–F**).

**Figure 47.**
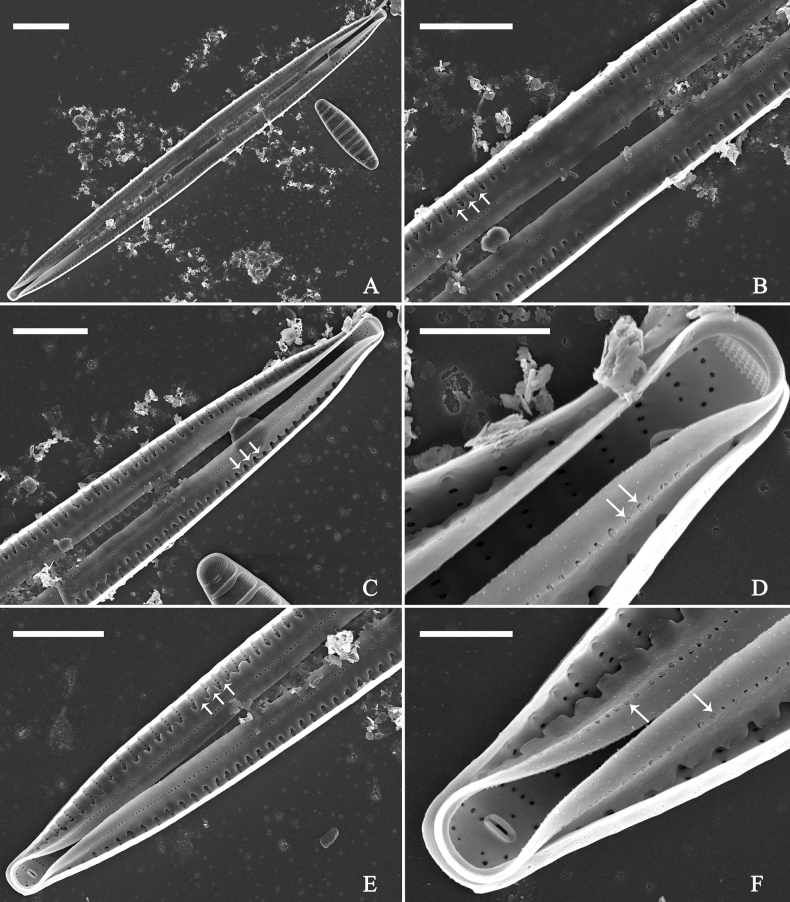
*Ulnariamenyuanensis* sp. nov., internal view, SEM**A** a valve with associated valvocopula **B** middle part detail from **A**, note serrated projections (three arrows) **C, D** apical details from **A**, note serrated projections (**C**, three arrows) and closing plates (**D**, two arrows) **E, F** details from other apex from **A**, note serrated projections (**E**, three arrows) and closing plates (**F**, two arrows). Scale bars: 10 μm (**A**); 5 μm (**B, C, E**); 2 μm (**D, F**).

Figs 44 , 45 , 46 , 47

##### Holotype.

Slide JIUDIA202307, specimen circled on slide, illustrated as Fig. 44D.

##### Registration.

PhycoBank http://phycobank.org/103813

##### Type locality.

China. Qinghai province: Menyuan County, an unnamed river, at a sampling location named Kengtan (37°27'28"N, 101°23'15"E, 2940 m a.s.l.), collected by Bing Liu, July 18, 2019.

##### Description.

***LM*** (Fig. 44). Valves lanceolate (fusiform) with cuneate to rostrate apices (Fig. 44A–J, see also Figs 45A, 46A). Valve dimensions (n = 31): length 60–104 μm, wide 5–7 μm at centre. Sternum distinct, extending length of valve. Central area not clearly visible due to presence of many ghost striae (Fig. 44A–J, see also Figs 45B, E, F, 46B, E, F). Striae parallel, radiate only approaching each pole, 12–14 in 10 μm, denser near poles. Striae on both sides of sternum, some alternate, some opposite.

***SEM*** (Figs 45–47). Valve characterized by a series of relatively wide virgae, interconnected with vimines and closing plates affixed with a few struts to each areolar wall (Figs 45A–F, 46A–F, closing plate see Fig. 47D, F). Central area circumscribed by both short marginal striae and isolated areolae (Figs 45B, E, F, 46B, E, F). Unperforate internal shallow grooves distinct in central area (e.g., Fig. 46B, E, F). Ocellulimbus composed of ca. 13 pervalvar and 7 transverse rows of porelli. Striae uniseriate. Areolae not apically elongated from sternum to mantle (Fig. 46B–F). One rimoportula located at each pole, externally expressed as a simple hole (Fig. 45C, D), internally bilabiate, situated close to sternum (Fig. 46C, D). Valvocopula a closed hoop, surrounding the valve internal margin (Fig. 47A). Valvocopula bearing a mostly continuous row of poroids dividing pars interior from pars exterior, located at midline (Fig. 47B, C, E), lacking ornamentation at both poles (Fig. 47D, F). On its advalvar edge, valvocopula has a row of serrated projections, each corresponding internally to a virga (Fig. 47B, C, E, three arrows, respectively).

##### Etymology.

Named after Menyuan County, where the species was found.

##### Ecology and distribution.

*Ulnariamenyuanensis* was commonly found in the surface sediment collected in Kengtan with *U.blancoi*. Thus, *U.menyuanensis* lives on the stone surfaces of a plateau river. So far, its distribution is known only from the type locality.

##### Discussion.

*Ulnariamenyuanensis* is characterized by its fusiform valve outline, the presence of distinct ghost striae, and cuneate to rostrate apices. It differs from *U.ramesii* (Héribaud) T. Ohtsuka by the latter’s linear-lanceolate valve outline (see Morales et al. 2007, p. 73, figs 87–94, as Synedraulnavar.ramesi) and differs from *U.oxyrhynchus* (Kützing) Aboal by the latter’s acute apices (see Morales et al. 2007, p. 65, figs 62–67, as Synedraulnavar.oxyrhynchus). *Ulnariaverhaegeniana* Van de Vijver, De Haan, Mertens & Cocquyt has parallel margins almost to valve apices while *U.menyuanensis* has lanceolate valve outline and possesses distinct ghost striae (see Van de Vijver et al. 2017, p. 222 for comparison).

#### ﻿ Ulnaria neobiceps

Taxon classificationPlantaeLicmophoralesUlnariaceae

Bing Liu sp. nov.

AEFF919F-8334-5F81-B2E4-38D9EC4E5846

**Figure 48.**
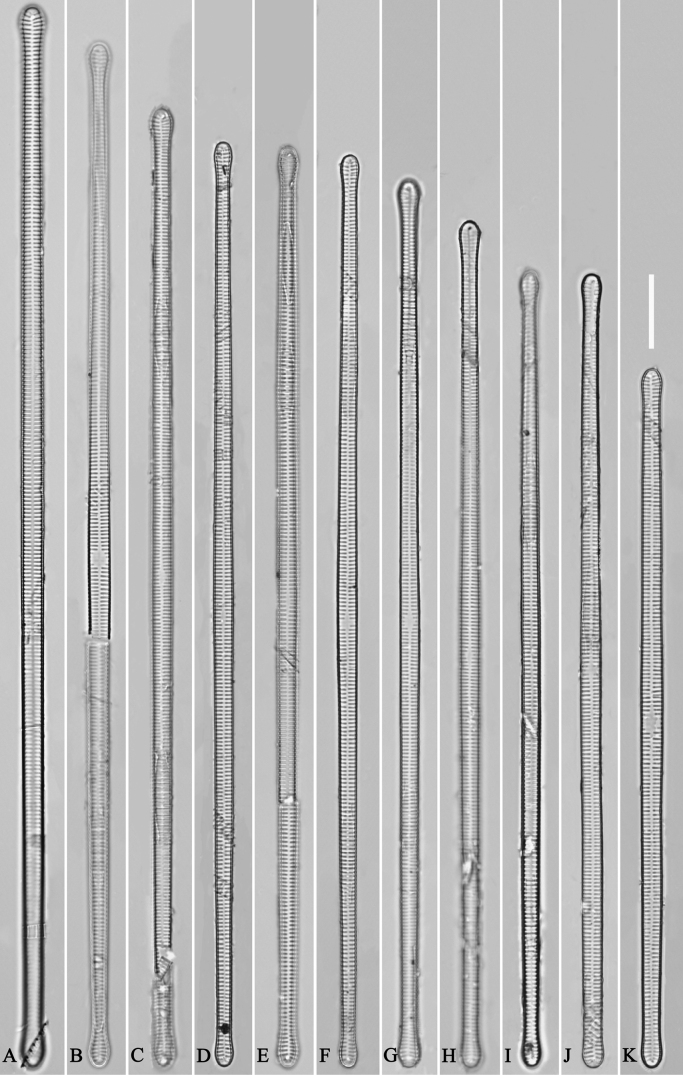
*Ulnarianeobiceps* sp. nov., ×400, LM**A–K** eleven valves exhibiting a size diminution series, note the linear-lanceolate valve outline and variable central areas **A** micrograph of holotype specimen. Scale bar: 20 μm.

**Figure 49.**
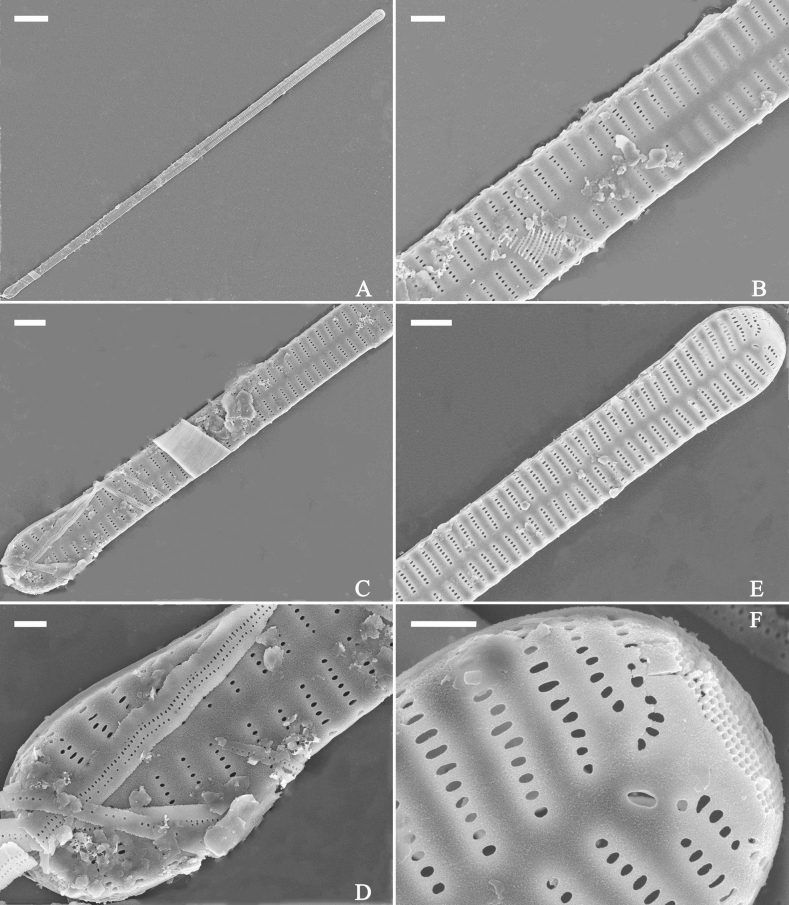
*Ulnarianeobiceps* sp. nov., external view, SEM**A** a complete valve **B** middle part detail from **A**, note central area complete absent **C–F** apical details from **A**, note the capitate apices. Scale bars: 20 μm (**A**); 2 μm (**B**); 3 μm (**C–E**); 1 μm (**F**).

**Figure 50.**
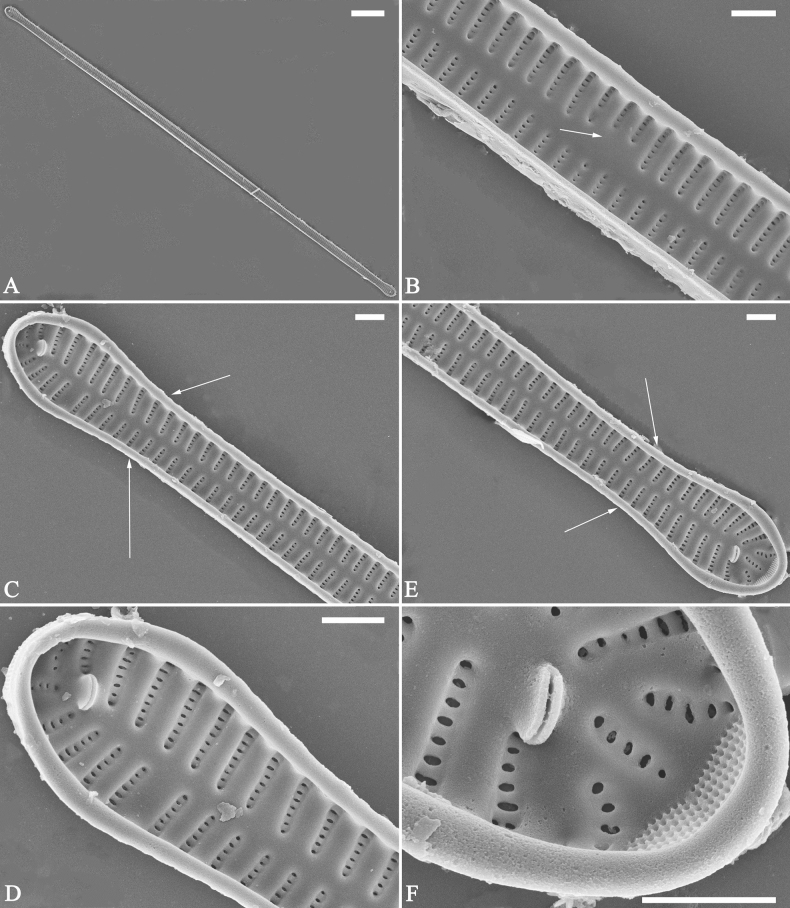
*Ulnarianeobiceps* sp. nov., internal view, SEM**A** a complete valve **B** middle part detail from **A**, note the very small central area (arrow) **C, E** two poles from **A**, note that valve margins do not constrict near the capitate apices (two arrows, respectively) **D, F** two apical details, note the capitate apices and apical pore field. Scale bars: 20 μm (**A**); 2 μm (**B–F**).

**Figure 51.**
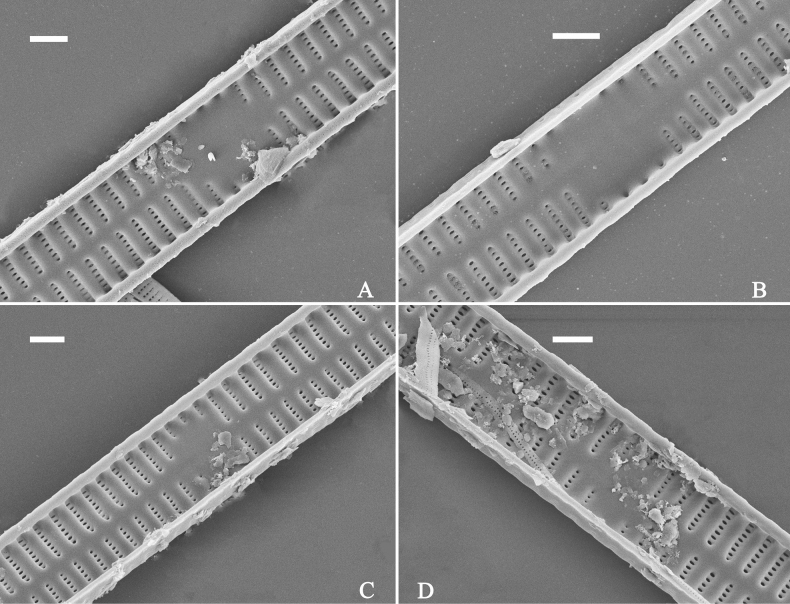
*Ulnarianeobiceps* sp. nov., internal view, SEM**A–D** four middle valve parts showing very variable central areas. Scale bars: 2 μm (**A–D**).

**Figure 52.**
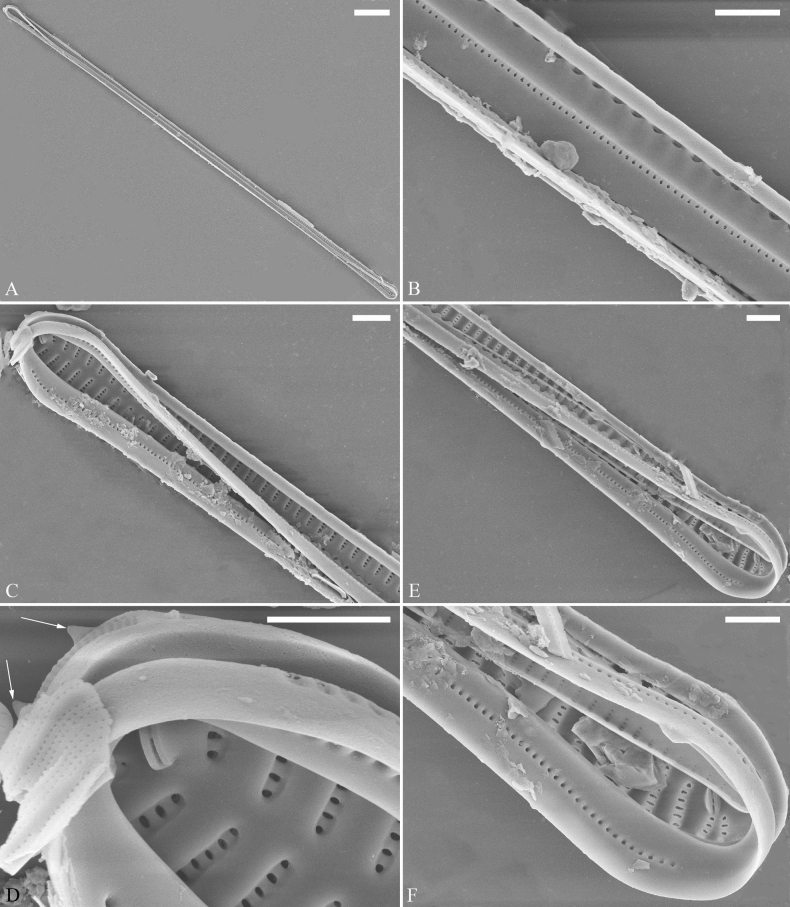
*Ulnarianeobiceps* sp. nov., internal view, SEM**A** a valve with valvocopula **B–F** details from **A**, showing the valvocopula structure, note two horn-like projections protruding over ocellulimbus. Scale bars: 20 μm (**A**); 3 μm (**B, C, E**); 2 μm (**D, F**).

**Figure 53.**
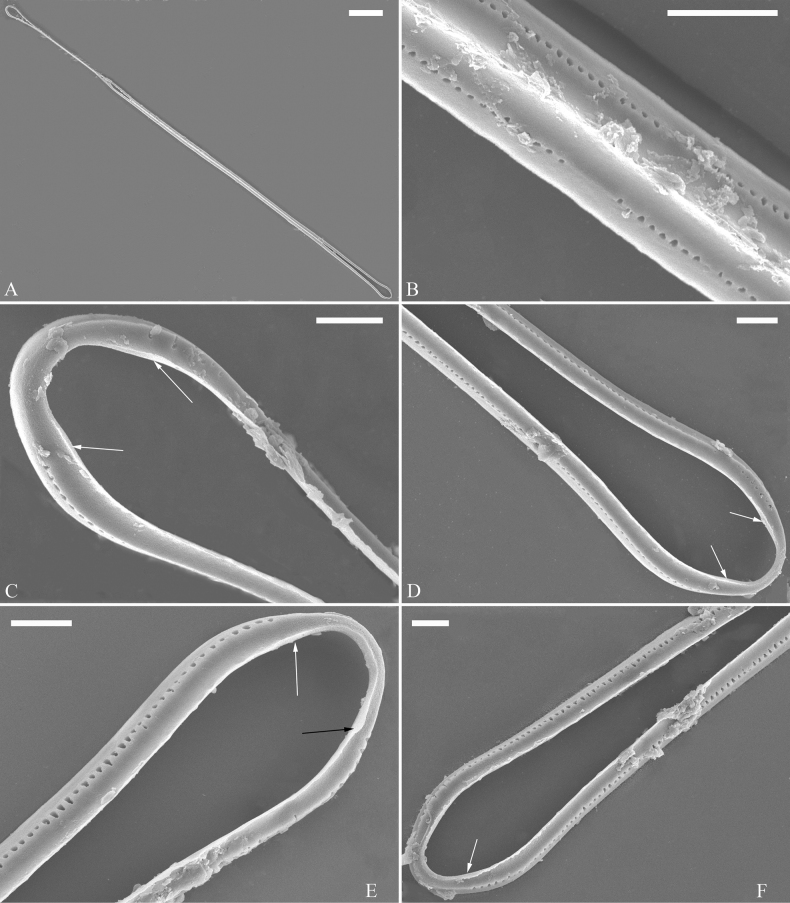
*Ulnarianeobiceps* sp. nov., valvocopula, SEM**A** a complete valvocopula **B–D** details from **A**, showing row of poroids interrupted in the middle and shelf-like projection at each apex (**C**, **D**, two arrows respectively) **E, F** other two apical details of valvocopula have the same apical structure. Scale bars: 20 μm (**A**); 3 μm (**B**); 2 μm (**C–F**).

Figs 48 , 49 , 50 , 51 , 52 , 53

##### Holotype.

Slide JIUDIA202308, specimen circled on slide, illustrated as Fig. 48A.

##### Registration.

PhycoBank http://phycobank.org/103814.

##### Type locality.

China. Qinghai province: Menyuan County, an unnamed river, at a sampling location named Kengtan (37°27'28"N, 101°23'15"E, 2940 m a.s.l.), collected by Bing Liu, July 18, 2019.

##### Description.

***LM*** (Fig. 48). Valves linear-lanceolate with distinct capitate apices (Fig. 48A–K, see also Figs 49A, 50A). Valve dimensions (n = 23): length 202–307 μm, width 4.5–6.7 μm at centre. Sternum distinct, extending length of valve. Central area very variable: hyaline region extending to both margins (e.g., Fig. 48F, K), can be circumscribed by both short marginal striae and isolated areolae (e.g., Fig. 48B, C, G, J), nearly absent (Fig. 48H) or completely lacking (Fig. 48A, D, E, I). Striae parallel, radiate only approaching each pole, 9–11 in 10 μm, increasing near each pole. Striae on both sides of sternum, some opposite each other, and others alternate.

***SEM*** (Figs 49–53). Valve characterized by a series of relatively wide virgae and interconnected with vimines (Fig. 49B–F). Central area completely lacking (Fig. 49B), hyaline area circumscribed by both short marginal striae and isolated areolae (Figs 50B, 51A, C, D), or hyaline area extending to both margins (Fig. 51B). Striae uniseriate, on both sides of sternum, opposite or alternate, areolae gradually become elongated from sternum to mantle (Figs 50B–F, 51A–D). Ocellulimbus composed of ca. 24 pervalvar and 8 transverse rows of porelli. Two horn-like projections protruding over the ocellulimbus (Fig. 52D, two arrows). One rimoportula located at each pole, externally expressed as a simple hole (Fig. 49F), internally bilabiate, situated close to sternum at an angle (Fig. 50D, F). Valvocopula closed, surrounding the valve internal margin (Fig. 52A), bearing a mostly continuous midline row of poroids dividing pars interior from pars exterior (Figs 52B–F, 53A–F), lacking ornamentation at both poles (Figs 52D, F, 52C–F). On its advalvar edge, valvocopula has a row of serrated projections, each corresponding internally to a virga (Fig. 52B, C, E). Shelf-like projections present at pars interior of each apex (Fig. 53C–F, two arrows, respectively).

##### Etymology.

The epithet *biceps* has been occupied by *Ulnariabiceps* (Kützing) Compère, so here the epithet *neobiceps* is used, which is a combination of *neo* (new) and *biceps* (two capitate) reflecting its two distinctly capitate poles.

##### Ecology and distribution.

*Ulnarianeobiceps*, *U.blancoi*, and *U.menyuanensis* were commonly found in the same sampling site of Kengtan. Thus, *U.neobiceps* lives on the stone surfaces of a plateau river. So far, its distribution is known only from the type locality.

##### Discussion.

*Ulnarianeobiceps* is characterized by its linear-lanceolate valve outline, variable central area, distinctly capitate apices, and long and slender valve. Both *U.neobiceps* and *U.capitata* (Ehrenberg) Compère have capitate apices, but the apices of *U.capitata* are rhomboid-capitate (see Morales et al. 2007, p. 49, figs 13–17; p. 51, figs 18–20, as *Synedracapitata*) whereas the apices of *U.neobiceps* are rounded-capitate. Moreover, *U.neobiceps* has very variable central area while *U.capitata* lacks central area.

#### ﻿ Ulnaria chengduoensis

Taxon classificationPlantaeLicmophoralesUlnariaceae

Bing Liu sp. nov.

82643DCA-64CC-50BB-A3EA-F40492A5647F

Figs 54 , 55 , 56

##### Holotype.

**Figure 54.**
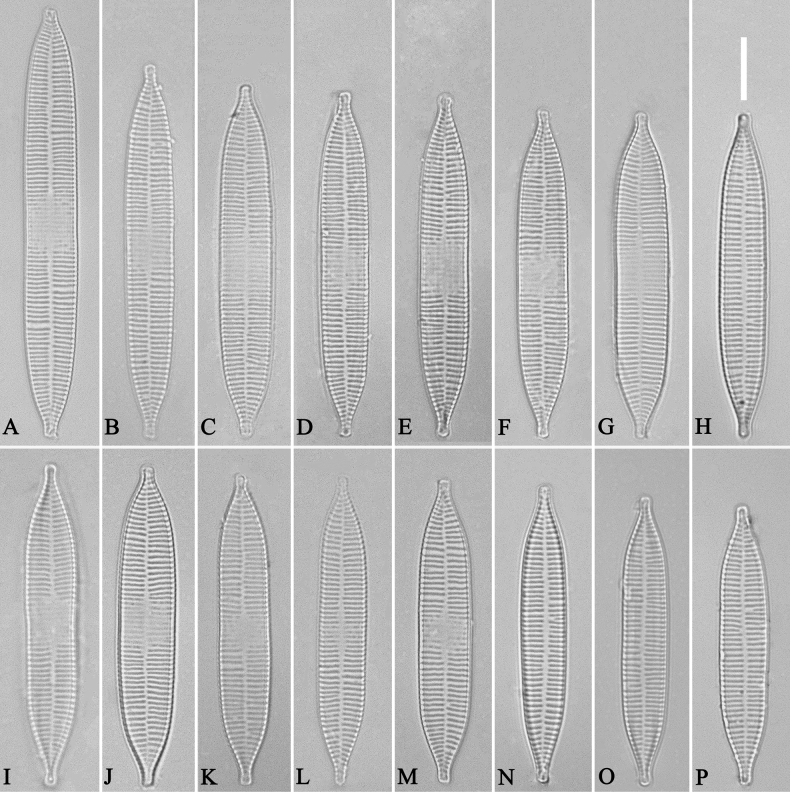
*Ulnariachengduoensis* sp. nov., ×1000, LM**A–P** sixteen valves exhibiting a size diminution series, note the linear-lanceolate valve outline and variable central areas **A** micrograph of holotype specimen. Scale bar: 10 μm.

Slide JIUDIA202309, specimen circled on slide, illustrated as Fig. 54A.

##### Registration.

PhycoBank http://phycobank.org/103815.

##### Type locality.

China. Qinghai province: Chengduo County, Baima River, at a sampling location (33°22'21"N, 97°0'18"E, 3690 m a.s.l.), collected by Bing Liu, July 22, 2019.

##### Description.

**Figure 55.**
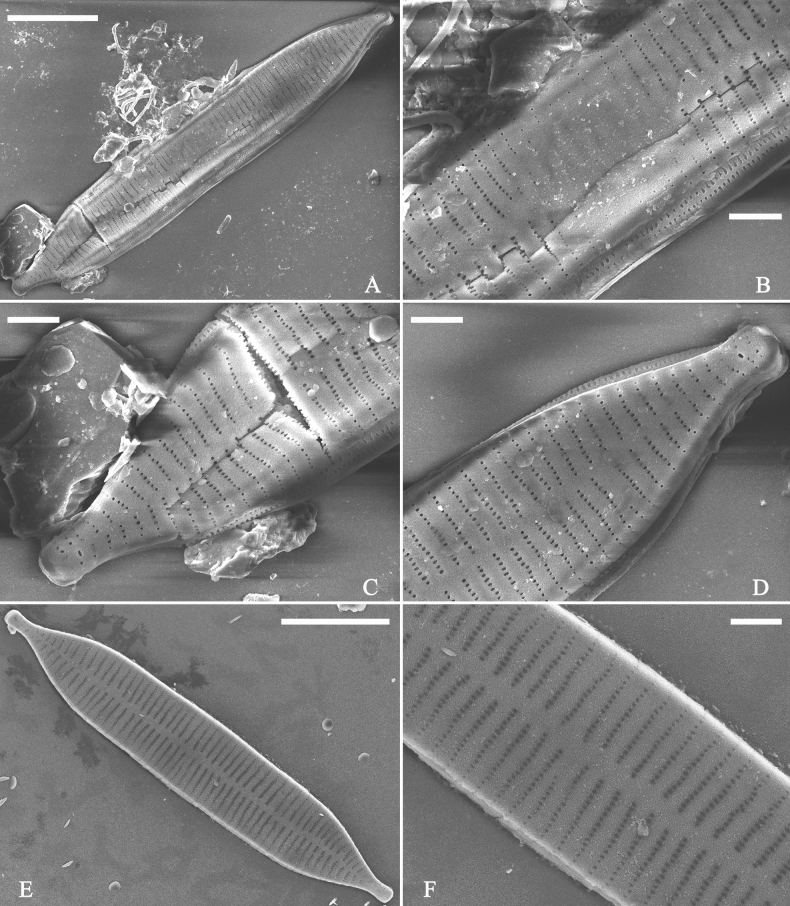
*Ulnariachengduoensis* sp. nov., external view, SEM**A** a broken valve **B** middle part detail from **A**, note the central area flanked by marginal short striae **C, D** two apical details from **A**, note the rostrate apices **E** a complete valve **F** middle part detail from **E**, note central area absent. Scale bars: 10 μm (**A, E**); 2 μm (**B–D, F**).

**Figure 56.**
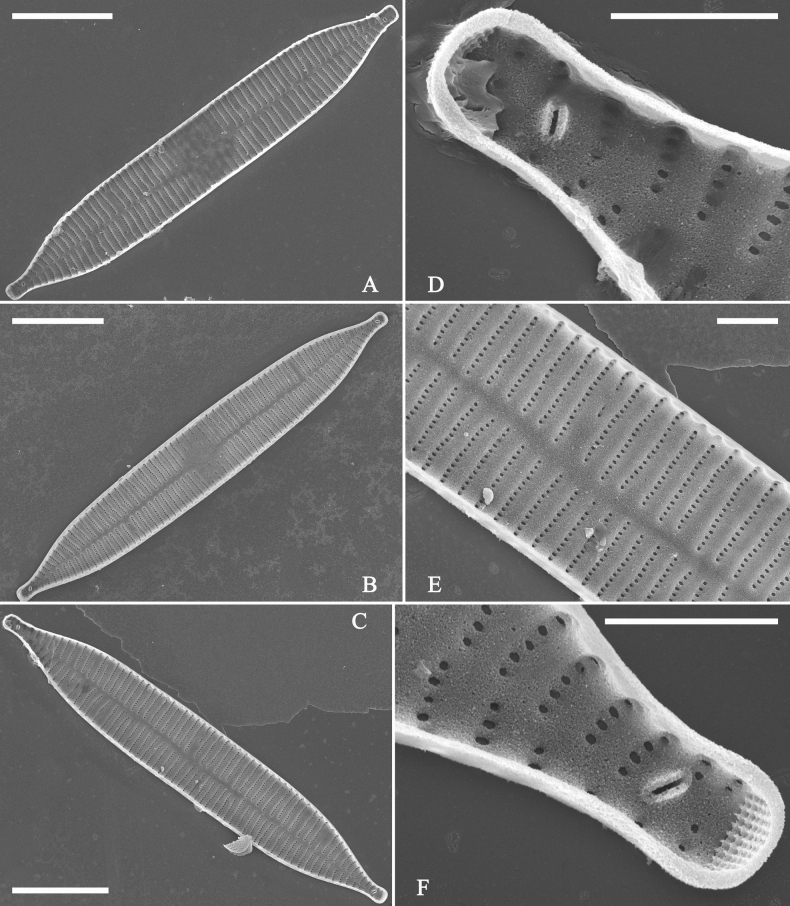
*Ulnariachengduoensis* sp. nov., internal view, SEM**A–C** three complete valves, note the linear-lanceolate valve outlines and variable central areas **D–F** details from **C**, note rostrate apices and absent central area. Scale bars: 10 μm (**A–C**); 2 μm (**D, E**); 1 μm (**F**).

***LM*** (Fig. 54). Valves linear with rostrate apices (Fig. 54A–P, see also Figs 55E, 56A–C). Valve dimensions (n = 51): length 42–66 μm, width 6–8 μm at centre. Sternum distinct, extending length of valve. Central area not clearly visible due to presence of ghost striae (Fig. 54A–G, I–K, M) or completely lacking (Fig. 54H, L, N–P). Striae parallel, radiate only approaching each pole, 12–15 in 10 μm. Across sternum some striae opposite one another, others alternate.

***SEM*** (Figs 55, 56). Valve characterized by a series of relatively wide virgae, interconnected with vimines (Fig. 55B–D). Striae uniseriate, areolae often apically elongated (Fig. 56E). Central area variable: a hyaline area circumscribed by both short marginal striae and isolated areolae (Figs 55B, 56A, B), or completely absent (Figs 55F, 56E). One rimoportula located at each pole, externally expressed as a simple hole (Fig. 55C, D), internally bilabiate, situated close to sternum (Fig. 56D, F). Ocellulimbus composed of ca. 12 pervalvar and 9 transverse rows of porelli (Fig. 56F).

##### Etymology.

Named after Chengduo County, where this species was found.

##### Ecology and distribution.

Epilithic in a plateau river. The following environmental parameters were measured in the field: Conductivity was 422.7 ± 1.3 μS∙cm^-1^, pH was 8.2 ± 0.1 and water temperature was 12.2 ± 0.5 °C. So far, its distribution is known only from the type locality.

##### Discussion.

*Ulnariachengduoensis* is characterized by its linear valve outline, variable central area, narrow rostrate valve apices. It differs from *U.ramerii* and *U.verhaegeniana* by its variable central area, i.e., it may completely lack a central area whereas *U.ramerii* always possesses an ellipsoid to rectangular central area (see Morales et al. 2007, p. 73, figs 87–94, as Synedraulnavar.ramesi) and *U.verhaegeniana* always has a large, apically elongated, rectangular central area (see Van de Vijver et al. 2017, p. 223, figs 1–13).

#### ﻿ Ulnaria qinghainensis

Taxon classificationPlantaeLicmophoralesUlnariaceae

Bing Liu sp. nov.

DBEF86D6-86F0-551A-BC60-6131B3ADF5CF

**Figure 57.**
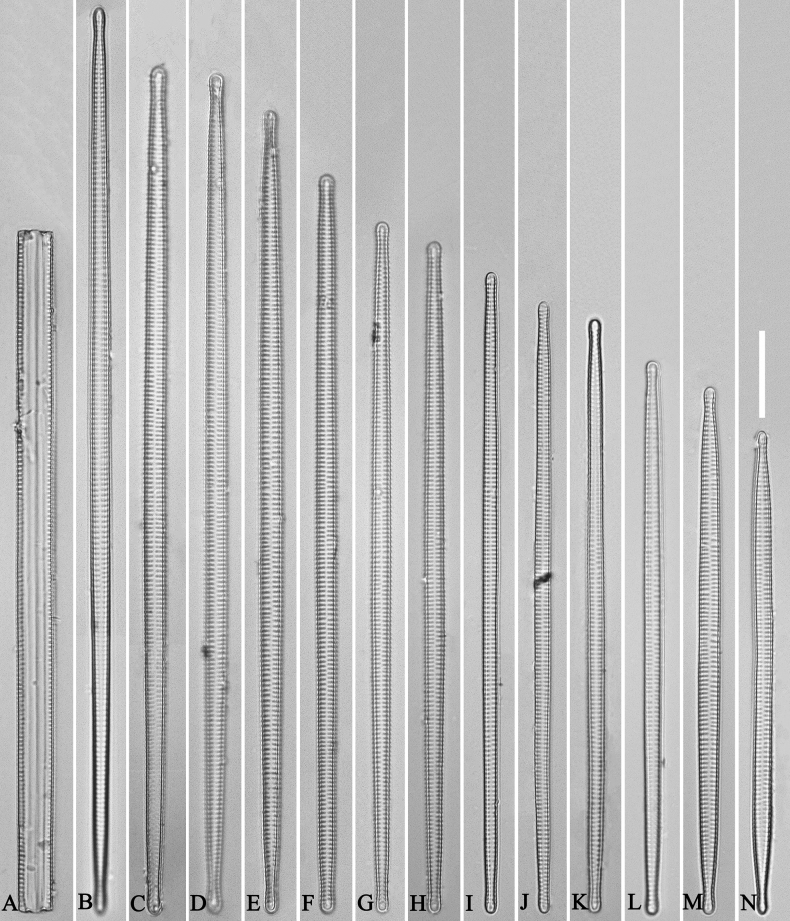
*Ulnariaqinghainensis* sp. nov., ×630, LM**A** a frustule in girdle view **B–N** thirteen valves exhibiting a size diminution series, note the narrowly lanceolate valve outlines and absent central areas **B** micrograph of holotype specimen. Scale bar: 20 μm.

**Figure 58.**
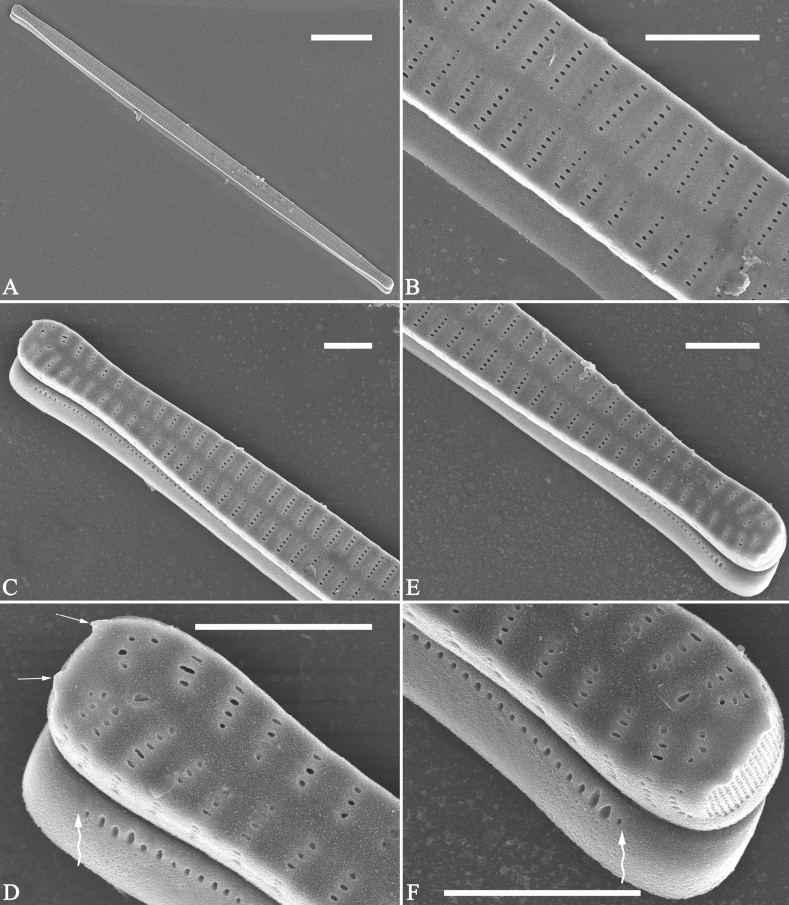
*Ulnariaqinghainensis* sp. nov., external view, SEM**A** a valve with valvocopula **B** detail of middle part from **A**, note absent central area **C–F** two poles from **A**, note two horn-like projections (**D**, two arrows) and row of poroids terminating before each apex of valvocopula (**E**, **F**, wavy arrow respectively). Scale bars: 20 μm (**A**); 4 μm (**B–F**).

**Figure 59.**
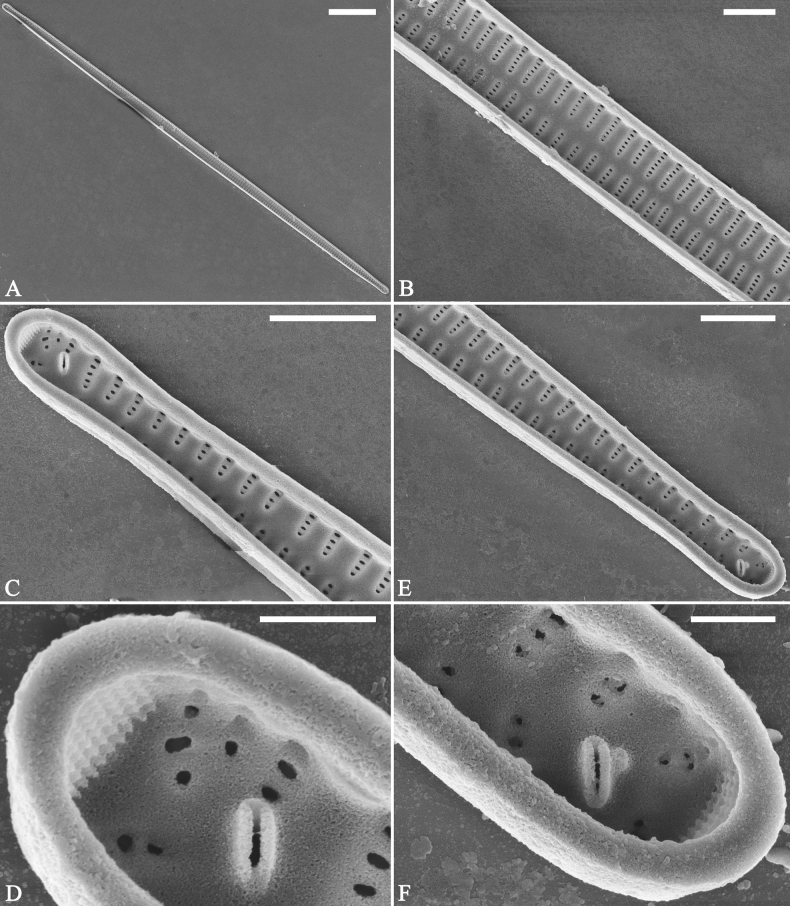
*Ulnariaqinghainensis* sp. nov., internal view, SEM**A** a complete valve **B** middle part from **A**, note central area is absent **C–F** two apical details from **A**. Scale bars: 20 μm (**A**); 4 μm (**B, C, E**); 1 μm (**D, F**).

**Figure 60.**
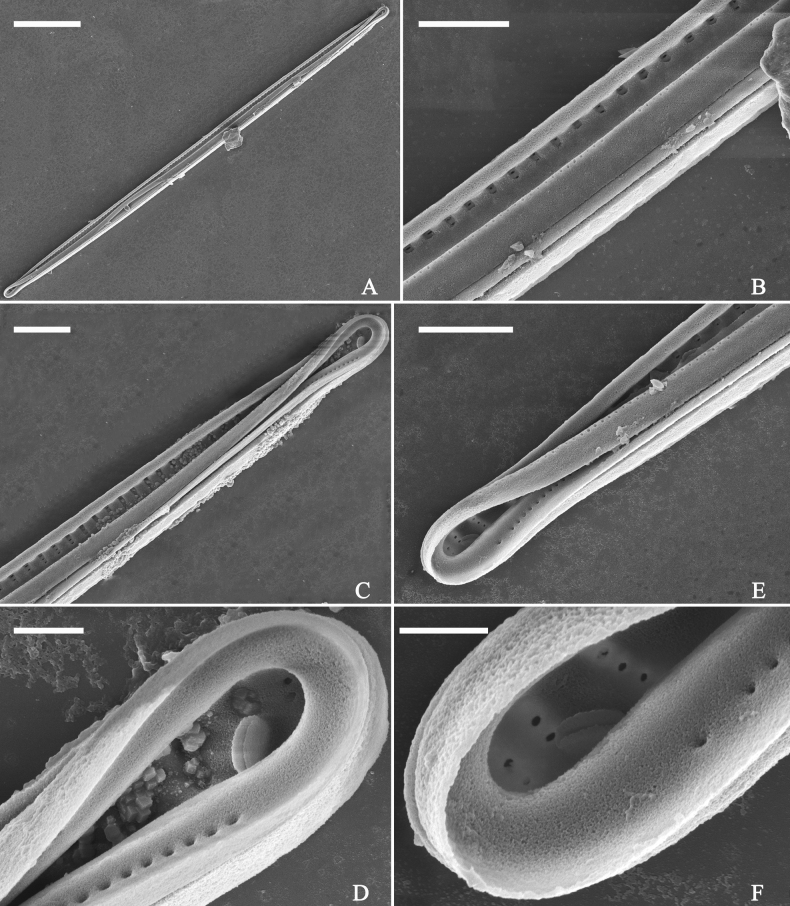
*Ulnariaqinghainensis* sp. nov., internal view, SEM**A** valve with valvocopula **B** middle part from **A**. **C–F** Two apical details from **A**. Scale bars: 20 μm (**A**); 4 μm (**B, C, E**); 1 μm (**D, F**).

Figs 57 , 58 , 59 , 60

##### Holotype.

Slide JIUDIA202310, specimen circled on slide, illustrated as Fig. 57C.

##### Registration.

PhycoBank http://phycobank.org/103816.

##### Type locality.

China. Qinghai province: Chengduo County, Baima River, at a sampling location (33°22'21"N, 97°0'18"E, 3690 m a.s.l.), collected by Bing Liu, July 22, 2019.

##### Description.

***LM*** (Fig. 57). Frustule rectangular in girdle view (Fig. 57A). Valves linear-lanceolate with sub-capitate apices (Fig. 57B–N, see also Figs 58A, 59A). Valve dimensions (n = 35): length 88–223 μm, width 3.1–5.0 μm at centre. Sternum distinct, extending length of valve. Central area completely absent. Striae parallel, radiate at apices, and mostly opposite one another across sternum. Stria density 9–11 in 10 μm.

***SEM*** (Figs 58–60). Valve characterized by a series of relatively wide virgae, interconnected with vimines (Fig. 58B–F). Striae uniseriate. Stria mostly opposite across sternum, equidistant until radiate at poles, areolae often apically elongated, especially near the mantle (Figs 58B–F, 59B, C, E). Central area completely absent (Figs 58B, 59B). Ocellulimbus composed of ca. 18 pervalvar and 9 transverse rows of porelli. Two horn-like projections protruding over the ocellulimbus (Fig. 58D, two arrows). One rimoportula located at each pole, externally expressed as a simple hole (Fig. 58D, F), internally bilabiate, situated close to sternum (Fig. 59D, F). Valvocopula closed, surrounding the valve internal margin (Fig. 60A), bearing a mostly continuous row of poroids dividing pars interior from pars exterior, located at the midline (Fig. 60B–F), lacking ornamentation at both poles (Fig. 60D, F). On its advalvar edge, valvocopula has a row of serrated projections, each corresponding internally to a virga (Fig. 60B, C, E).

##### Etymology.

Named after Qinghai province, where this species was found.

##### Ecology and distribution.

Epilithic in a plateau river. *Ulnariaqinghainensis* and *U.chengduoensis* were commonly found in the same sampling site. The environmental parameters were measured in the field and the results are given in the description of the former taxon above. So far, its distribution is known only from the type locality.

##### Discussion.

*Ulnariaqinghainensis* is characterized by its linear-lanceolate valve outline, lacking central area, sub-capitate apices, and slender valves. It differs from *U.obtusa* (W. Smith) E. Reichardt by its narrower valves (3.1–5.0 μm vs 5–8 μm) and its much higher stria density (9–11 in 10 μm vs 3–4 in 10 μm) (see Williams and Van de Vijver 2021, p. 175).

#### ﻿ Ulnaria fanjingensis

Taxon classificationPlantaeLicmophoralesUlnariaceae

Bing Liu sp. nov.

FE58F79A-717B-52AB-BFB5-BCEDE4E737A7

**Figure 61.**
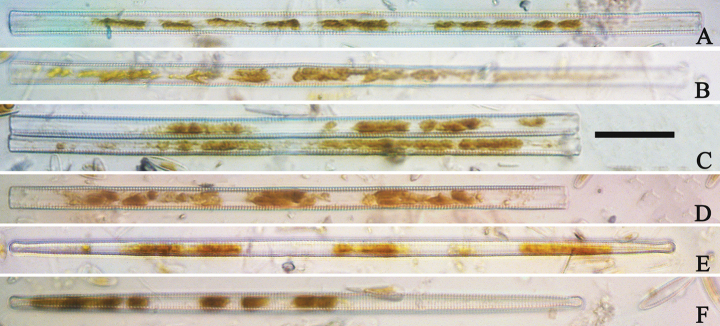
*Ulnariafanjingensis* sp. nov., ×400, LM**A–D** living cells in girdle view, note numerous plate-like chromatophores **E, F** two living cells in valve view, note numerous plate-like chromatophores. Scale bar: 20 μm.

**Figure 62.**
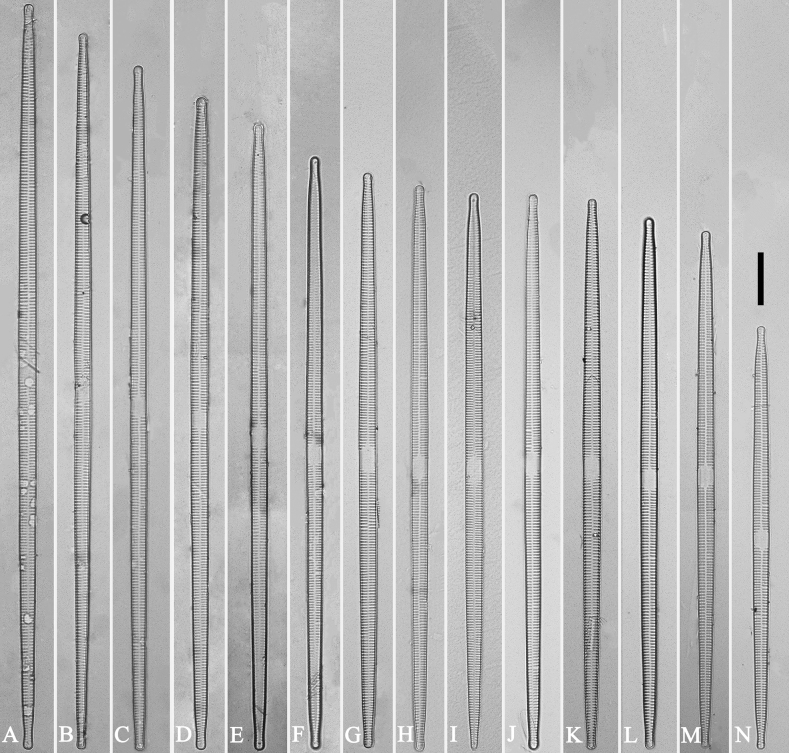
*Ulnariafanjingensis* sp. nov., ×400, LM**A–N** fourteen valves exhibiting a size diminution series, note the lanceolate valve outline and hyaline central area **C** micrograph of holotype specimen. Scale bar: 20 μm.

**Figure 63.**
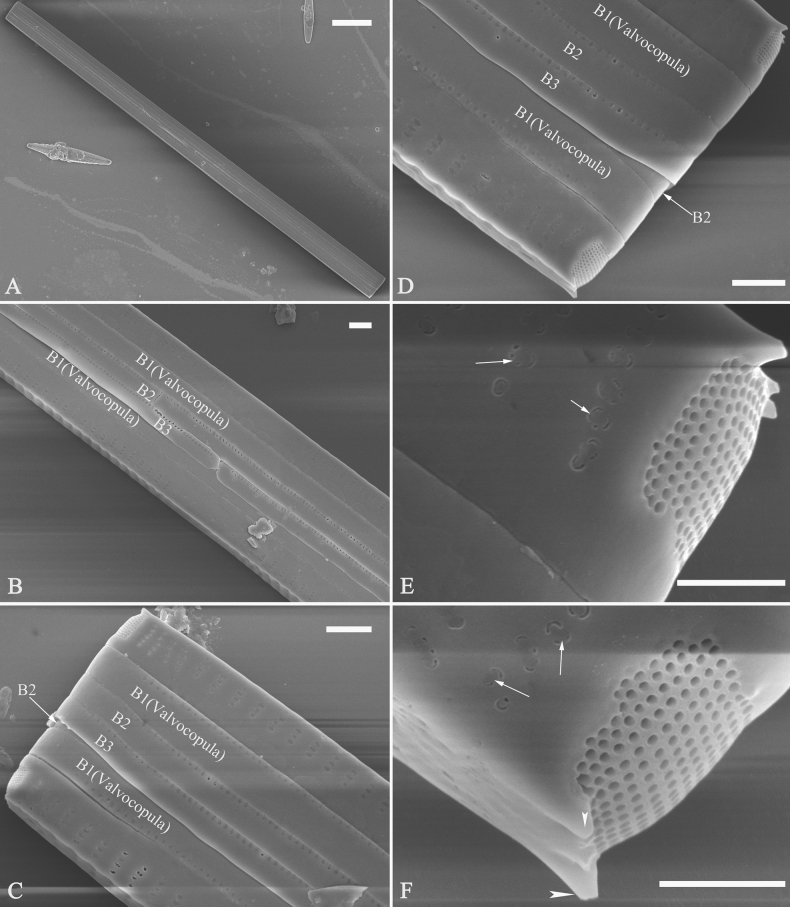
*Ulnariafanjingensis* sp. nov., girdle view, SEM. **A** a frustule in girdle view **B–D** details from **A**, note the 3:2 configuration of girdle bands **E, F** apical details from **D**, showing the closing plates (two arrows respectively) and two horn-like projections (**F**, two arrowheads). Scale bars: 20 μm (**A**); 2 μm (**B–D**); 1 μm (**E, F**).

**Figure 64.**
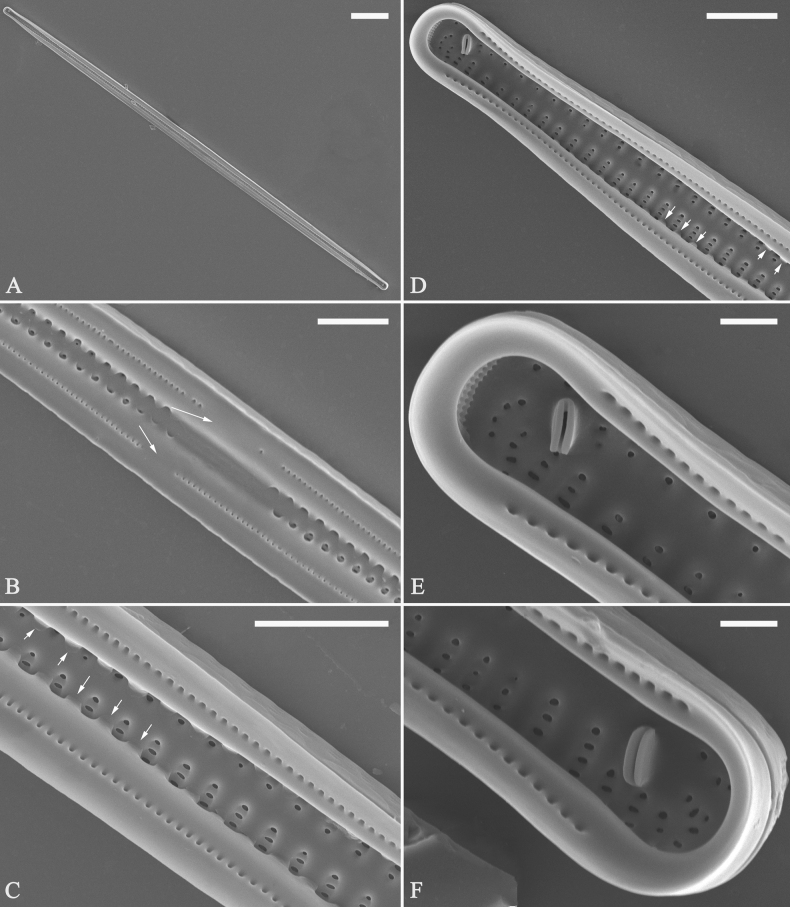
*Ulnariafanjingensis* sp. nov., internal view, SEM. **A** a valve with valvocopula **B** detail from **A**, note the row of poroids interrupted in the middle (two arrows) **C, D** details from **A**. **E, F** Two apical details from **A**. Scale bars: 20 μm (**A**), 4 μm (**B–D**), 1 μm (**E, F**).

**Figure 65.**
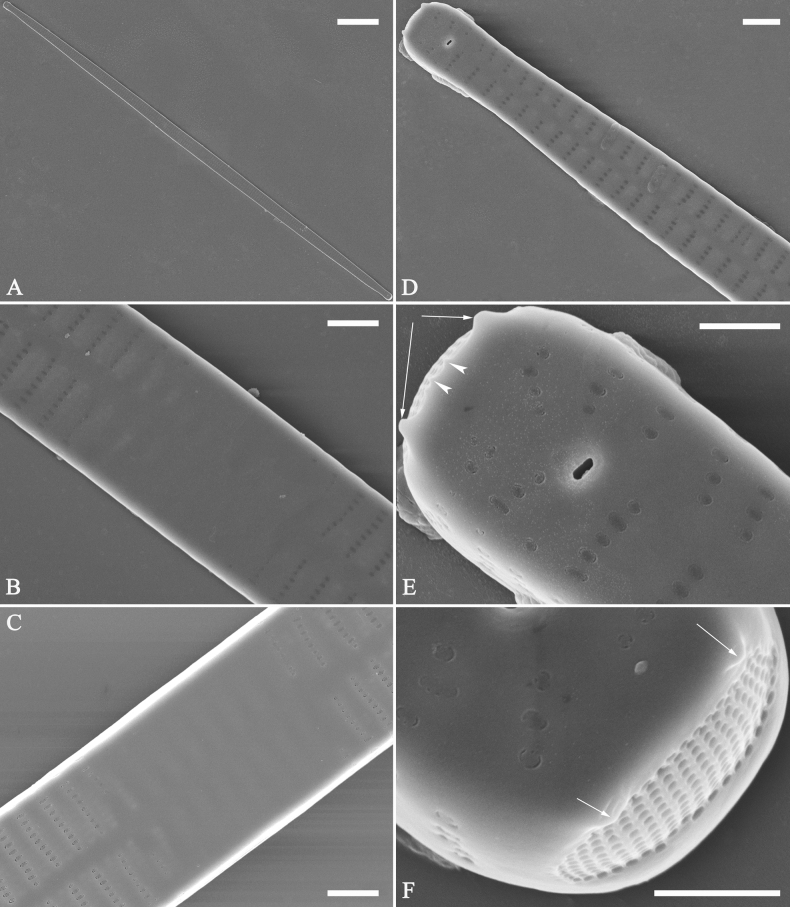
*Ulnariafanjingensis* sp. nov., external view, SEM**A** a complete valve **B** middle detail from **A**, showing the clear central area **C** middle detail of another valve showing the central area **D** a pole from **A**, note the slightly radiated striae and sub-capitate apex **E, F** two apices from **A**, note two largest horn-like projections and ocellulimbus. Scale bars: 20 μm (**A**); 2 μm (**B–D**); 1 μm (**E, F**).

**Figure 66.**
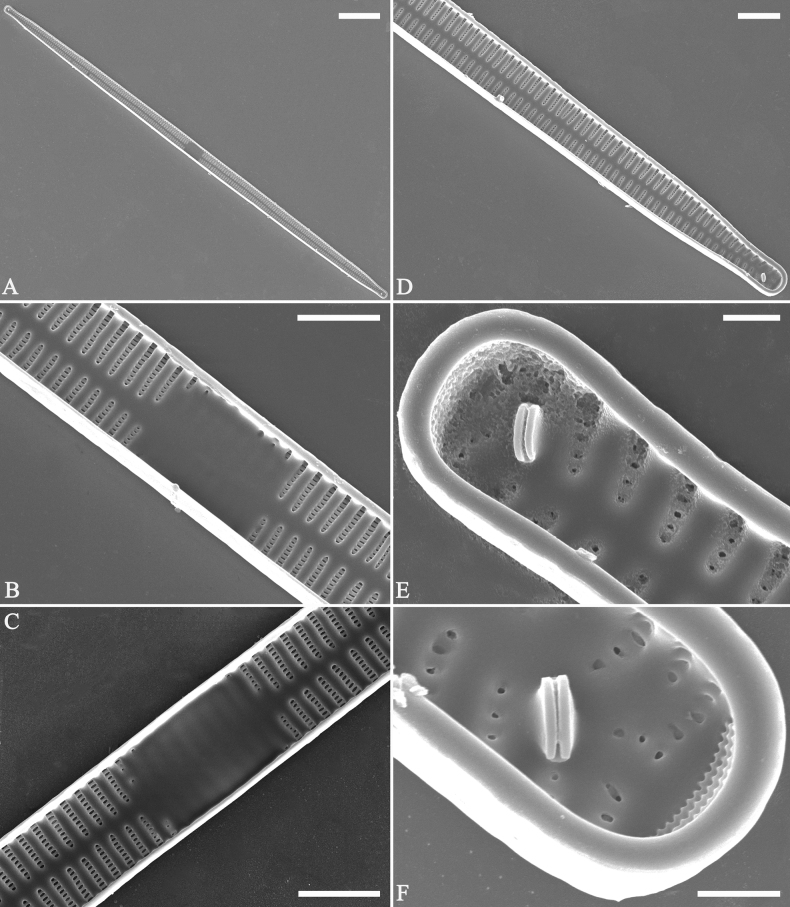
*Ulnariafanjingensis* sp. nov., internal view, SEM**A** a complete valve **B** middle part detail from **A**, note the clear central area **C** detail of another valve center showing the clear central area **D** a pole from **A**, note the tapering valve and rostrate apex **E, F** two apical details from **A**, note bilabiate rimoportulae and rostrate apices. Scale bars: 20 μm (**A**); 5 μm (**B–D**); 1 μm (**E, F**).

Figs 61 , 62 , 63 , 64 , 65 , 66

##### Holotype.

Slide JIUDIA202311, specimen circled on slide, illustrated as Fig. 62D.

##### Registration.

PhycoBank http://phycobank.org/103817.

##### Type locality.

China. Guizhou province: Fanjing Mountain National Nature Reserve, Heiwang river, near Mile Daochang (27°49'10"N, 108°46'18"E, 494 m a.s.l.), collected by Bing Liu, December 31, 2015.

##### Description.

***LM*** (Figs 61, 62). Living cells with numerous plate-like chromatophores (Fig. 61A–F, note that these cells may be in an unhealthy condition). Valves lanceolate, narrow, with rostrate to sub-capitate apices (Figs 61E, F, 62A–N). Valve dimensions (n = 56): length 165–291 μm, width 4.8–6.3 μm at centre. Sternum distinct, extending length of valve. Central area extending to both margins forming apically rectangular fascia. Striae parallel, radiate only approaching each apex, and mostly opposite one another across sternum. Stria density 9–12 (often 10) in 10 μm.

***SEM*** (Figs 63–66). Frustule rectangular in girdle view (Fig. 63A, see also Fig. 61A–C). Striae continuing onto mantle, absent from centre (Fig. 63B). Epivalve associated with valvocopula and two copulae (Fig. 63B–D, bands labelled B1–B3, B1 = valvocopula); hypovalve associated with valvocopula and one copula (Fig. 63C, D, bands labelled B1–B2, B1 = valvocopula), forming a 3:2 configuration of girdle bands. Valvocopula a closed hoop, same shape as valve outline, closely attached to mantle interior (Fig. 64A), bearing a mostly continuous row of poroids dividing pars interior from pars exterior, located at the midline (Fig. 64B–F), interrupted in the middle (Fig. 64B, two arrows). A row of serrated projections is present on its advalvar edge, each corresponding internally to a virga (Fig. 64C, D, five arrows respectively), lacking ornamentation at both poles (Fig. 64E, F). Each ocellulimbus composed of ca. 12 transverse rows (unequal in length) and ca. 18 pervalvar rows (unequal in length) (Figs 63E, F, 65F). A few serrated apical outgrowths protruding over ocellulimbus, largest two appearing horn-like (Figs 63F, 65E, F, two arrows, respectively). Valve face and mantle intersecting almost at right angle (Fig. 65A–F). Valve characterized by a series of relatively wide virgae, interconnected with thin vimines and closing plates affixed with a few struts to each areolar wall (Fig. 65D–F). Valves with uniseriate striae; each series situated mostly opposite each other, equidistant until radiate at poles (Fig. 66A–F). Central area apically rectangular, extending to both margins (Figs 65B, C, external, 66B, C, internal). One rimoportula located at each pole (Figs 65E, 66E, F). External opening of rimoportula expressed as a simple hole (Fig. 65E); internally bilabiate, situated close to sternum, aligned with striae (Fig. 66E, F).

##### Etymology.

Named after Fanjing Mountain, where the species was found.

##### Ecology and distribution.

The sampling site is close to the headwaters of Heiwan River, which originates in the Fanjing Mountain National Nature Reserve. The diatom samples were scraped off of the stone surfaces. The following environmental parameters were measured in the field. Electric conductivity was 54.9 ± 1.4 μS∙cm^-1^, pH was 7.6 ± 0.1, and water temperature was10.4 ± 0.1 °C. Since the diatom sample was scraped off of the surfaces of stones and the conductivity is below 100 μS∙cm^-1^, *U.fanjingensis* can be considered an epilithic diatom characteristic of poor electrolyte content fresh water. So far, its distribution is known only from the type locality.

##### Discussion.

*Ulnariafanjingensis* is characterized by its lanceolate valve outline, apically rectangular central area, rostrate to sub-capitate apices, and long valves. It is similar to *U.dongtingensis* Bing Liu, but they have different valve outlines: *U.dongtingensis* has narrow-lanceolate valves with parallel central margins the length of the central area whereas *U.fanjingensis* has parallel central margins extending beyond the central area (see Liu et al. 2019b, p. 134, fig. 14).

#### ﻿ Ulnaria hupingensis

Taxon classificationPlantaeLicmophoralesUlnariaceae

Bing Liu sp. nov.

1CD231CE-B032-5419-BAB5-8A1D2DBBE385

**Figure 67.**
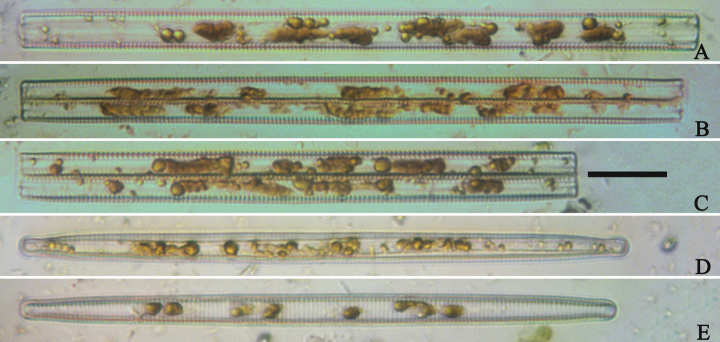
*Ulnariahupingensis* sp. nov., ×630, LM**A–C** living cells in girdle view, note numerous irregular chromatophores **D, E** two living cells in valve view, note numerous discoid chromatophores. Scale bar: 20 μm.

**Figure 68.**
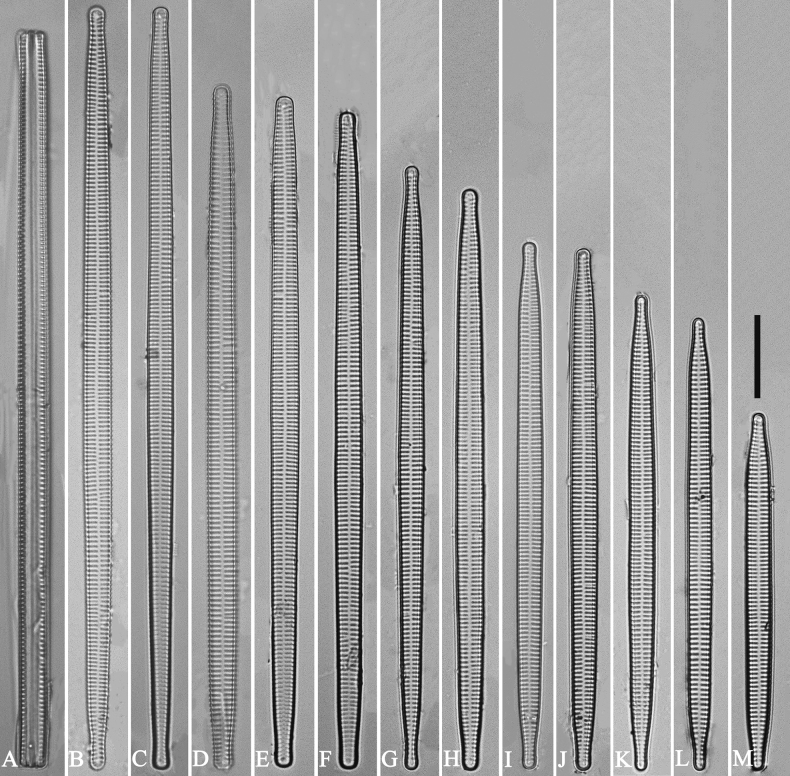
*Ulnariahupingensis* sp. nov., ×630, LM. **A** a frustule in girdle view **B–M** twelve valves exhibiting a size diminution series, note the lanceolate valve outline and completely absent central area **B** micrograph of holotype specimen. Scale bar: 20 μm.

**Figure 69.**
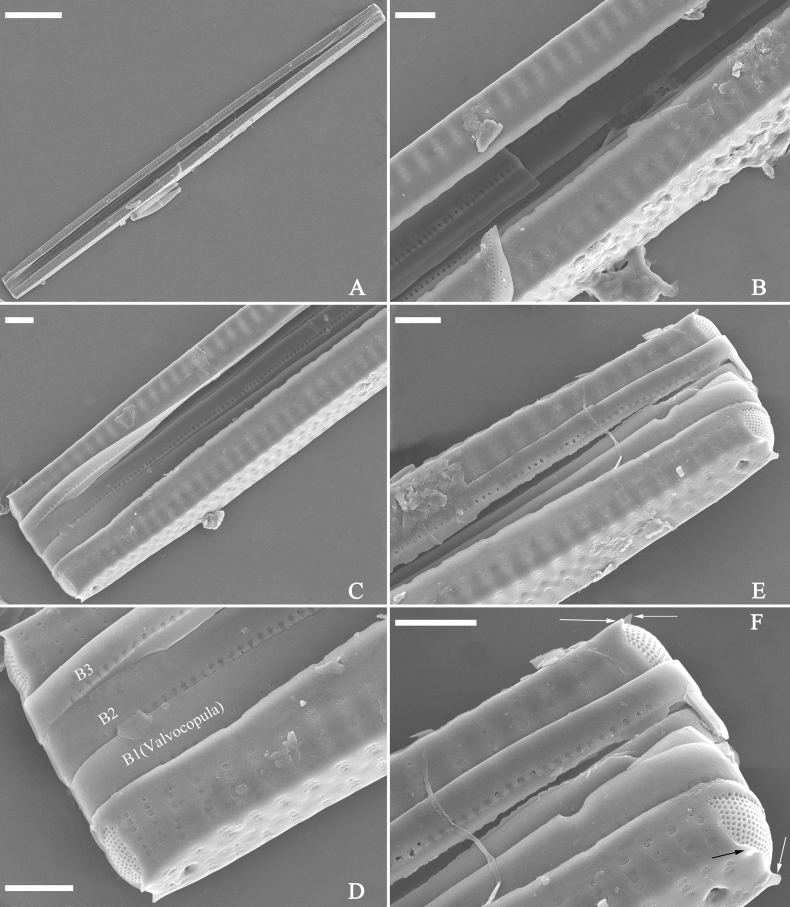
*Ulnariahupingensis* sp. nov., girdle view, SEM**A** a frustule in girdle view **B–E** details from **A**, note three copulae (labelled B1 to B3) associated with the epivalve **F** apical detail from **E**, showing two horn-like projections (two arrows respectively). Scale bars: 20 μm (**A**); 2 μm (**B–F**).

**Figure 70.**
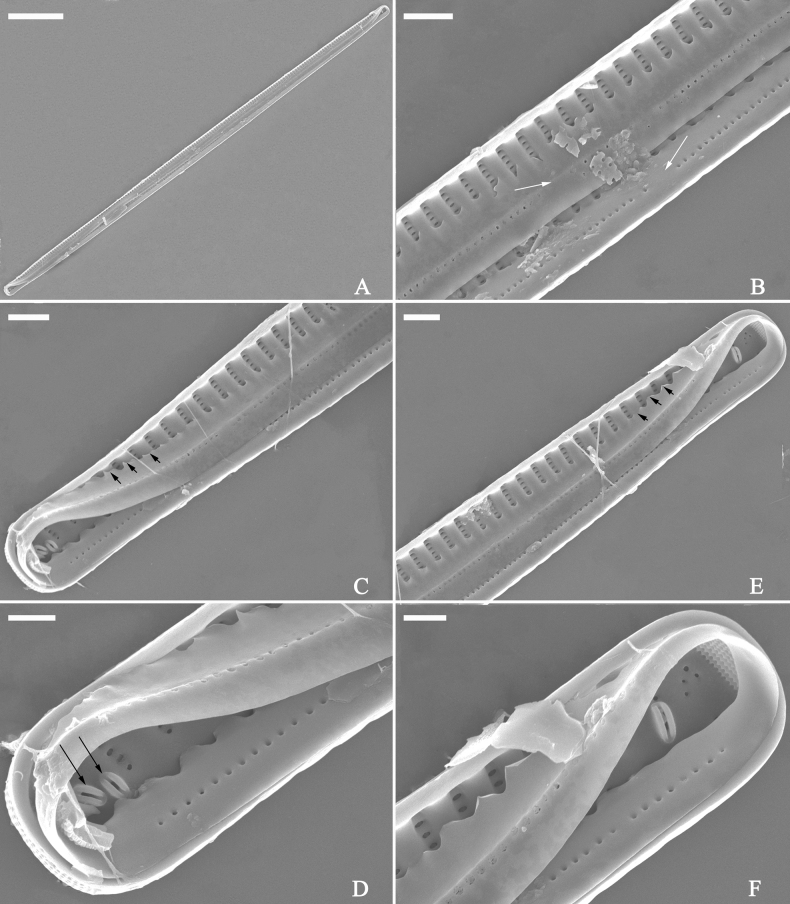
*Ulnariahupingensis* sp. nov., internal view, SEM**A** a valve with valvocopula **B** middle detail from **A**, note the row of poroids interrupted in the middle (two arrows) **C, E** two poles from **A**. **D** Apical detail from **C**, note two rimoportulae at one apex (two arrows) **F** apical detail from **E**, note one rimoportula at one apex. Scale bars: 20 μm (**A**); 2 μm (**B–F**).

**Figure 71.**
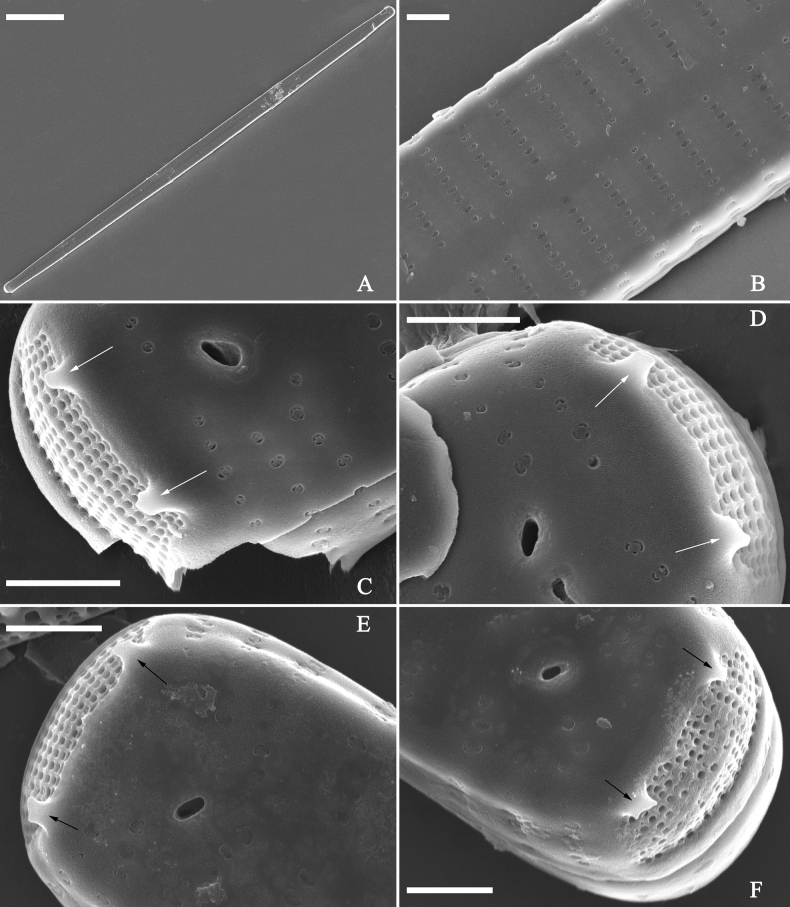
*Ulnariahupingensis* sp. nov., external view, SEM**A** a complete valve **B** middle part detail from **A**, note central area complete absent **C, D** two apical details from **A**, showing two horn-like projections protruding over the oceullulimbus (two arrows, respectively) **E, F** two other apical details showing two horn-like projections protruding over the ocellulimbus (two arrows, respectively). Scale bars: 20 μm (**A**); 1 μm (**B–F**).

**Figure 72.**
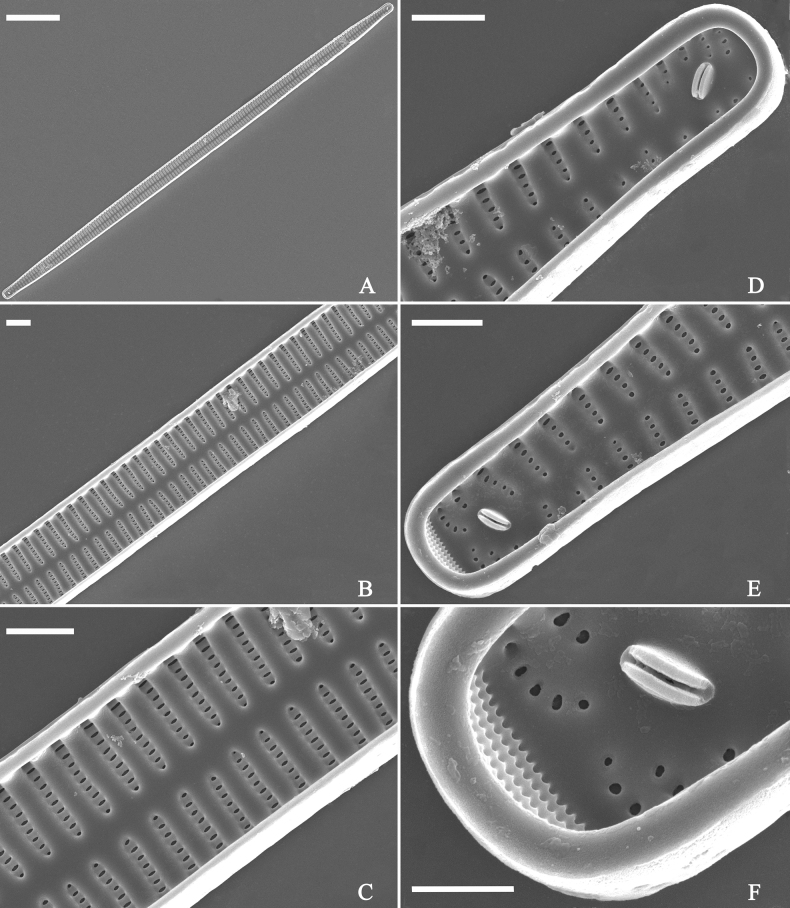
*Ulnariahupingensis* sp. nov., internal view, SEM**A** a complete valve **B, C** middle part details from **A**, note central area is completely absent **D–F** apical details from **A**. Scale bars: 20 μm (**A**); 2 μm (**B–F**).

Figs 67 , 68 , 69 , 70 , 71 , 72

##### Holotype.

Slide JIUDIA202312, specimen circled on slide, illustrated as Fig. 68B.

##### Registration.

PhycoBank http://phycobank.org/103818.

##### Type locality.

China. Hunan province: Huping Mountain National Nature Reserve, Xie River, a sampling location (30°1'9"N, 110°37'46"E, 400 m a.s.l.), collected by Bing Liu, March 14, 2021.

##### Description.

***LM*** (Figs 67, 68). Living cells with numerous discoid chromatophores (Fig. 67A–E, note that these cells may be in an unhealthy condition). Frustule in girdle view rectangular (Figs 67A–C, 68A, see also Fig. 69A). Valves lanceolate with protracted, rostrate to sub-capitate poles (Figs 67D, E, 68B–M, see also Figs 70A, 71A, 72A). Valve dimensions (n = 59): length 81–200 μm, width 5.4–7.0 μm at centre. Sternum distinct, extending length of the valve. Central area completely absent. Striae parallel, radiate only approaching each apex, and mostly opposite one another across sternum. Stria density 9.5–11.5 (often 11) in 10 μm.

***SEM*** (Figs 69–72). Frustule comprising several girdle bands (Fig. 69A–F). Epivalve associated with valvocopula and two copulae (Fig. 69D, labelled B1 to B3, B1 = Valvocopula); number of copulae associated with hypovalve not verified. Valvocopula a closed hoop, same shape as valve outline, closely attached to mantle interior, surrounding valve margin (Fig. 70A), bearing a mostly continuous row of poroids dividing pars interior from pars exterior, located at midline (Fig. 70B–F), poroids lacking at centre of the valve (Fig. 70B, two arrows). On its advalvar edge, valvocopula bears a row of serrated projections, each corresponding to a virga internally (Fig. 70C, E, three arrows, respectively). Valvocopula lacking ornamentation at both poles (Fig. 70D, F). Valve face and mantle intersecting almost at right angle (Fig. 71A, B). Valve characterized by a series of relatively wide virgae, interconnected with vimines and closing plates affixed with few struts to the areola wall (Fig. 71B). Striae uniseriate (Figs 71B, 72B–E), continuing onto mantle (Fig. 69B–F). Ocellulimbus composed of ca. 19 pervalvar and 11 transverse rows of porelli. Two horn-like projections protruding over the ocellulimbus (Figs 69F, 71C–F, two arrows, respectively). Internally, virgae transversely extending from sternum to mantle, striae situated almost opposite each other across sternum, areolae become more elongated closer to the mantle (Fig. 72B–E). Central area completely lacking (Figs 71A, B, external, 72A, B, internal). One rimoportula usually present at each pole, occasionally two produced at one end (Figs 70D, two arrows, 71D). External opening of rimoportula expresses as a simple hole, forming different shapes (Fig. 71C–F); internally bilabiate, situated close to sternum at an angle (Fig. 72D–F).

##### Etymology.

Named after Huping Mountain National Nature Reserve, where the species was found.

##### Ecology and distribution.

Epilithic in a mountain stream with oligotrophic waters. The following environmental parameters were measured in the field: Conductivity was 263 ± 1 μS∙cm^-1^, pH was 8.4 ± 0.1 and water temperature was 12.4 ± 0.4 °C. So far, its distribution is known only from the type locality.

##### Discussion.

*Ulnariahupingensis* is characterized by its lanceolate valve outline, lacking central area, and rostrate to sub-capitate apices. It differs from *U.qinghainensis* by its wider valves (5.4–7.0 μm vs 3.1–5.0 μm) and from *U.obtusa* by its much higher stria density (9.5–11.5 in 10 μm vs 3–4 in 10 μm) (see Williams and Van de Vijver 2021, p. 175).

#### ﻿ Ulnaria xieriverensis

Taxon classificationPlantaeLicmophoralesUlnariaceae

Bing Liu sp. nov.

72D21C66-FB8F-5BF5-802A-8EE629318A23

**Figure 73.**
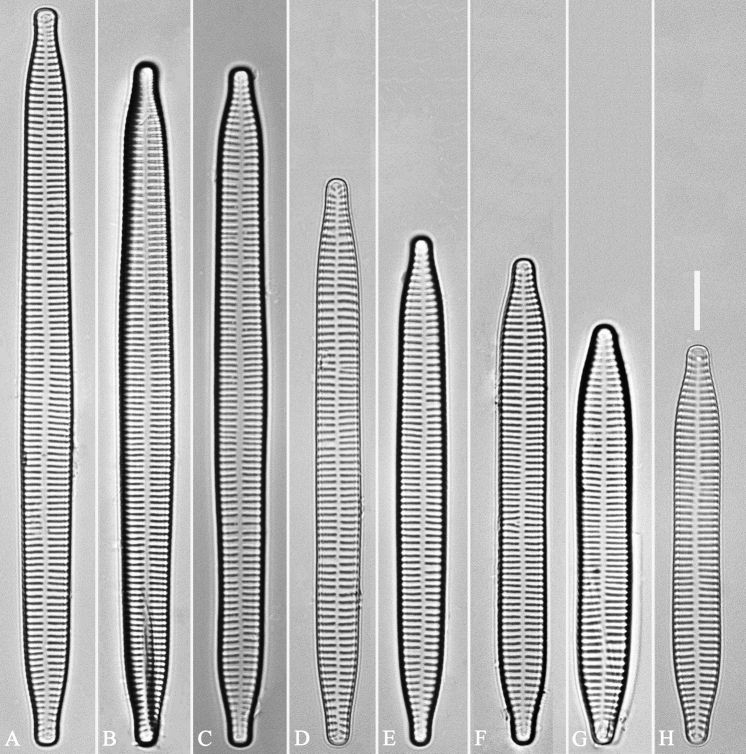
*Ulnariaxieriverensis* sp. nov., ×1000, LM**A–H** eight valves exhibiting a size diminution series, note the almost parallel but slightly undulate valve margins in larger specimens (**A–C**) and parallel valve margins in smaller specimens (**D–H**). **A** Micrograph of holotype specimen. Scale bar: 10 μm.

**Figure 74.**
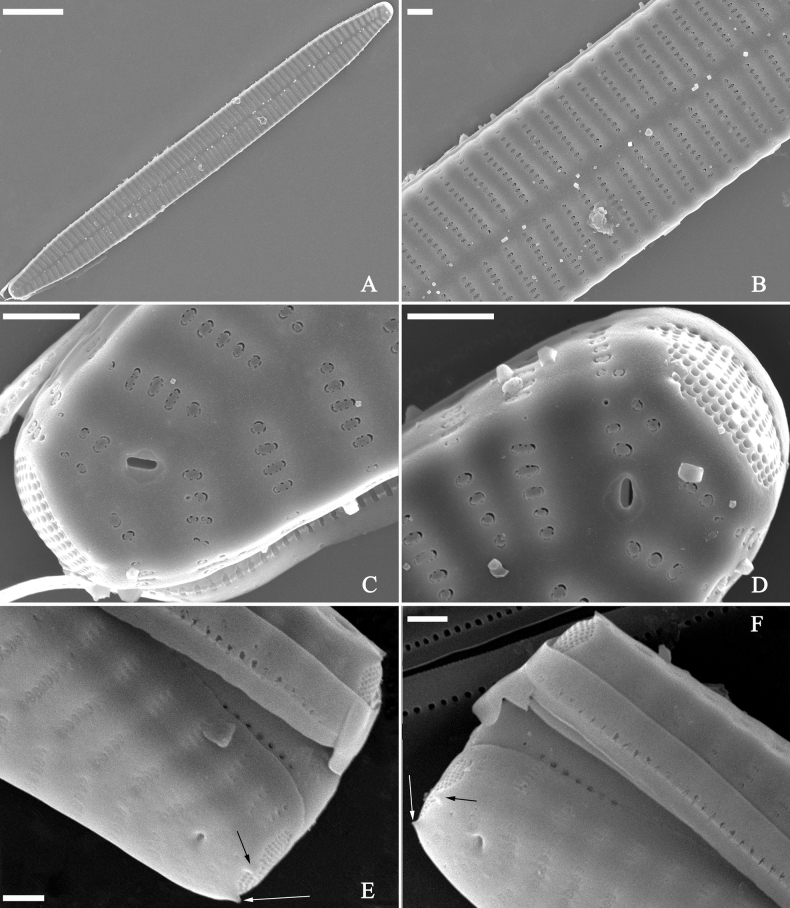
*Ulnariaxieriverensis* sp. nov., external view, SEM**A** a complete valve **B** middle part detail from **A**, note central area is completely absent **C, D** two apical details from **A**. **E, F** Two other apical details, note two horn-like projections protruding over the ocellulimbus (two arrows respectively). Scale bars: 10 μm (**A**); 1 μm (**B–F**).

**Figure 75.**
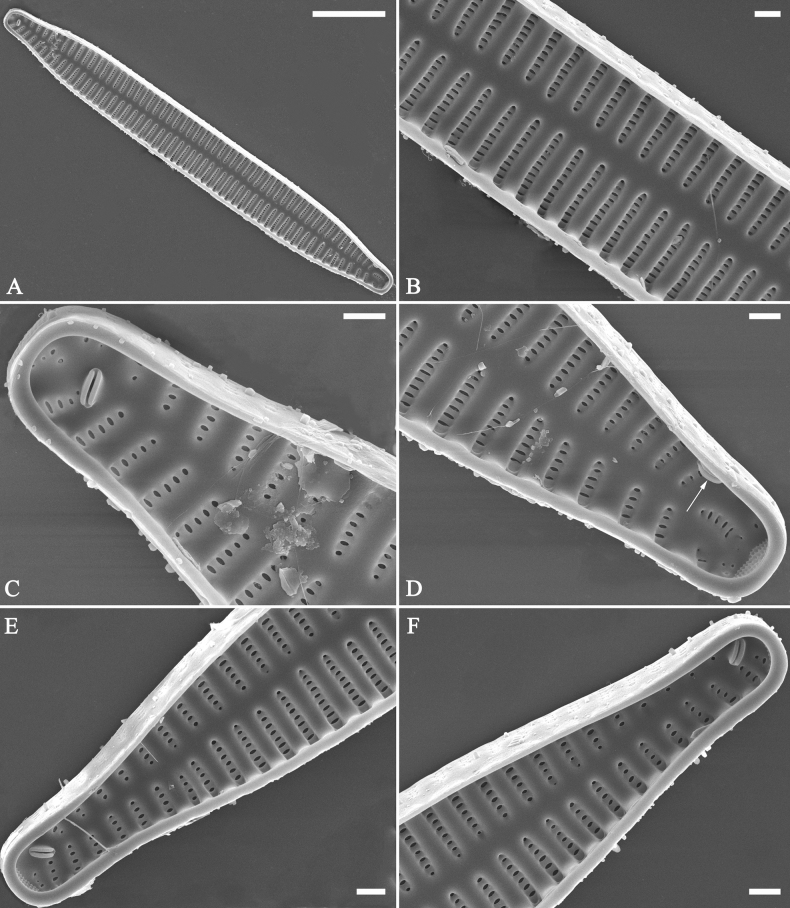
*Ulnariaxieriverensis* sp. nov., internal view, SEM**A** a complete valve **B** middle part detail from **A**, note central area is completely absent **C, D** two apical details from **A**, note one rimoportula located against the mantle (**D**, arrow) **E, F** two other apical details. Scale bars: 10 μm (**A**); 1 μm (**B–F**).

**Figure 76.**
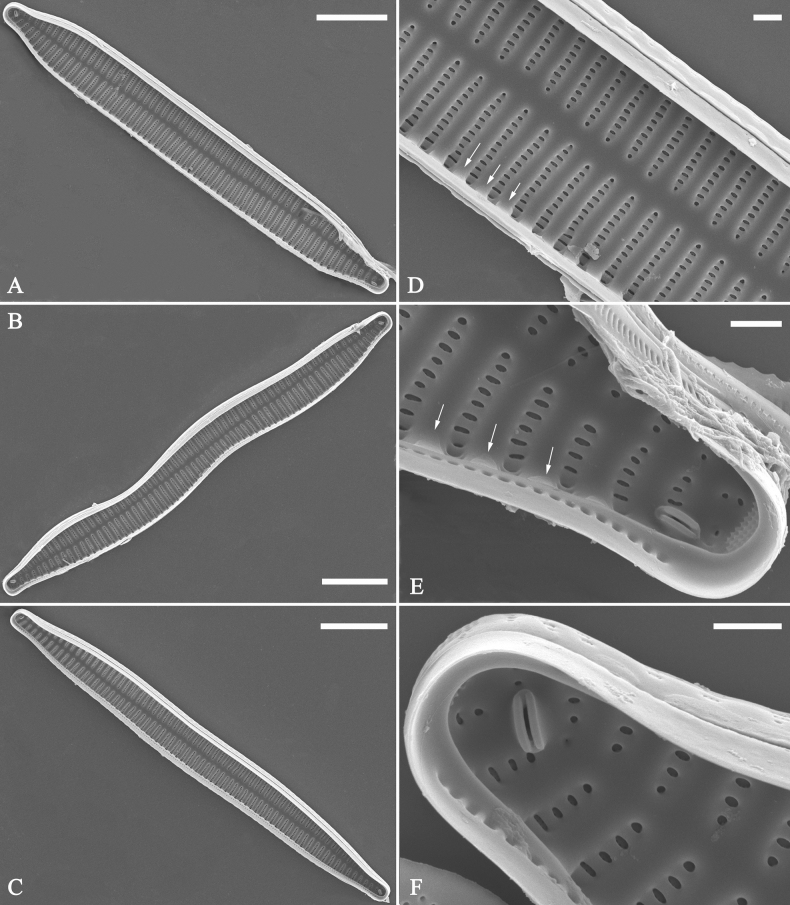
*Ulnariaxieriverensis* sp. nov., internal view, SEM**A** a complete valve with valvocopula **B, C** two complete valves with valvocopula, note undulate valve margins **D** middle detail from **A**, note the central area is completely absent and a row of serrated projections over virgae (three arrows) **E, F** two apical details from **A**, note serrated projections over virgae (**E**, three arrows). Scale bars: 10 μm (**A–C**); 1 μm (**D–F**).

Figs 73 , 74 , 75 , 76

##### Holotype.

Slide JIUDIA202313, specimen circled on slide, illustrated as Fig. 73A.

##### Registration.

PhycoBank http://phycobank.org/103819.

##### Type locality.

China. Hunan province: Huping Mountain National Nature Reserve, Xie River, a sampling location (30°1'9"N, 110°37'46"E, 400 m a.s.l.), collected by Bing Liu, March 14, 2021.

##### Description.

***LM*** (Figs 73). Valves generally linear (i.e., valve margins almost parallel), slightly undulate in larger specimens (Fig. 73A–C, see also Fig. 76B, C), parallel in smaller specimens (Fig. 73D–H, see also Figs 74A, 75A, 76A). Valve tapers towards apex, then it is protracted to broadly rostrate poles. Valve dimensions (n = 33): length 64–120 μm, width 6.0–8.6 μm at centre. Sternum distinct, extending length of valve. Central area completely absent. Striae parallel, radiate only approaching each pole, and mostly opposite one another across sternum. Stria density 10.5–12 (often 11) in 10 μm.

***SEM*** (Figs 69–72). Valve characterized by relatively wide virgae, interconnected with vimines, closing plates affixed with a few struts to the areolar wall (Fig. 74B–D). Ocellulimbus composed of ca. 16 pervalvar and 9 transverse rows of porelli. Two horn-like projections protruding over the ocellulimbus (Fig. 74E, F, two arrows, respectively). Striae uniseriate. Stria situated opposite each other across sternum, equidistant until radiate at poles, areolae become elongated closer to the mantle (Fig. 75B–F). One rimoportula present at each pole, externally expressed as a simple hole (Fig. 74C–F), internally bilabiate, situated close to sternum (Fig. 75C, E, F), sometimes displaced, against mantle (Fig. 75D). Central area completely lacking (Figs 74A, B, 75A, B, 76A–D). Valvocopula a closed hoop (Fig. 76A–C). Valvocopula bearing a mostly continuous row of poroids dividing pars interior from pars exterior, located at the midline (Fig. 76D–F), lacking ornamentation at both poles (Fig. 76E, F). On its advalvar edge, valvocopula has a row of serrated projections, each corresponding internally to a virga (Fig. 76D, E, three arrows, respectively).

##### Etymology.

Named after Xie River, where the species was found.

##### Ecology and distribution.

Epilithic in a mountain stream with oligotrophic waters. *Ulnariahupingensis* was found in the same sampling site with *U.xieriverensis* and the environmental parameters see above. So far, its distribution is known only from the type locality.

##### Discussion.

*Ulnariaxieriverensis* is characterized by its linear valve outline, absence of central area, and rostrate apices. It differs from *U.hupingensis* by its linear valve outline.

#### ﻿ Ulnaria pandurata-uniseriata

Taxon classificationPlantaeLicmophoralesUlnariaceae

Bing Liu sp. nov.

729EB5F6-44B1-590D-B42A-51FDD3DFE2BE

**Figure 77.**
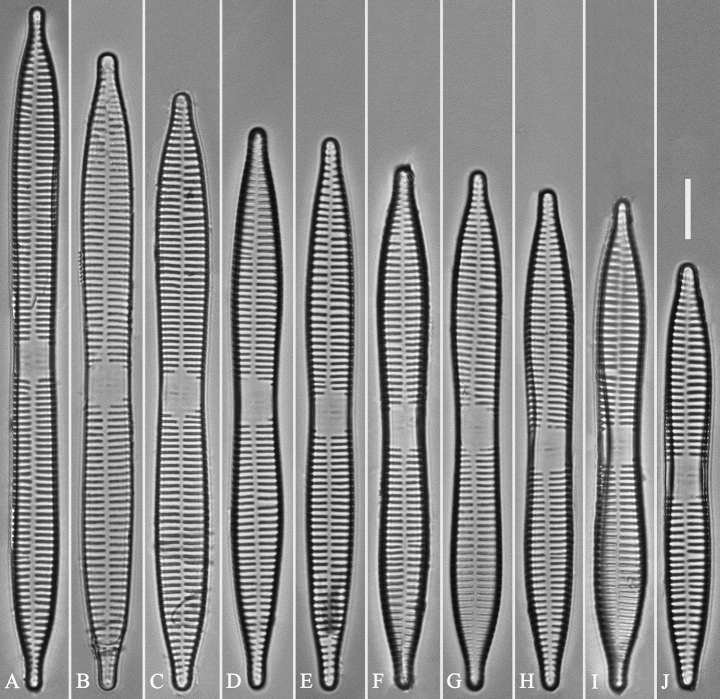
*Ulnariapandurata-uniseriata* sp. nov., ×1000, LM**A–J** ten valves exhibiting a size diminution series, note the panduriform valve outline **C** micrograph of holotype specimen. Scale bar: 10 μm.

**Figure 78.**
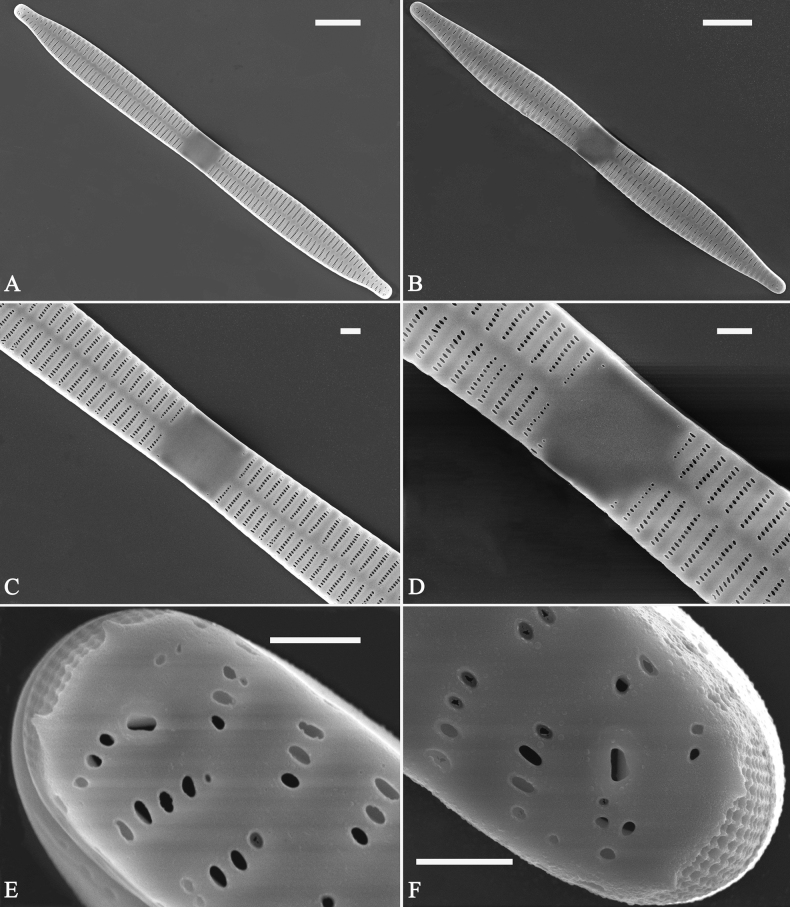
*Ulnariapandurata-uniseriata* sp. nov., external view, SEM**A, B** two complete valves, note panduriform valve outline **C, D** two middle part details from **A**, **B**, respectively, note rectangular central areas **E, F** two apical details from **B**, note two horn-like projections protruding over ocellulimbus. Scale bars: 10 μm (**A, B**); 1 μm (**C, D**); 2 μm (**E, F**).

**Figure 79.**
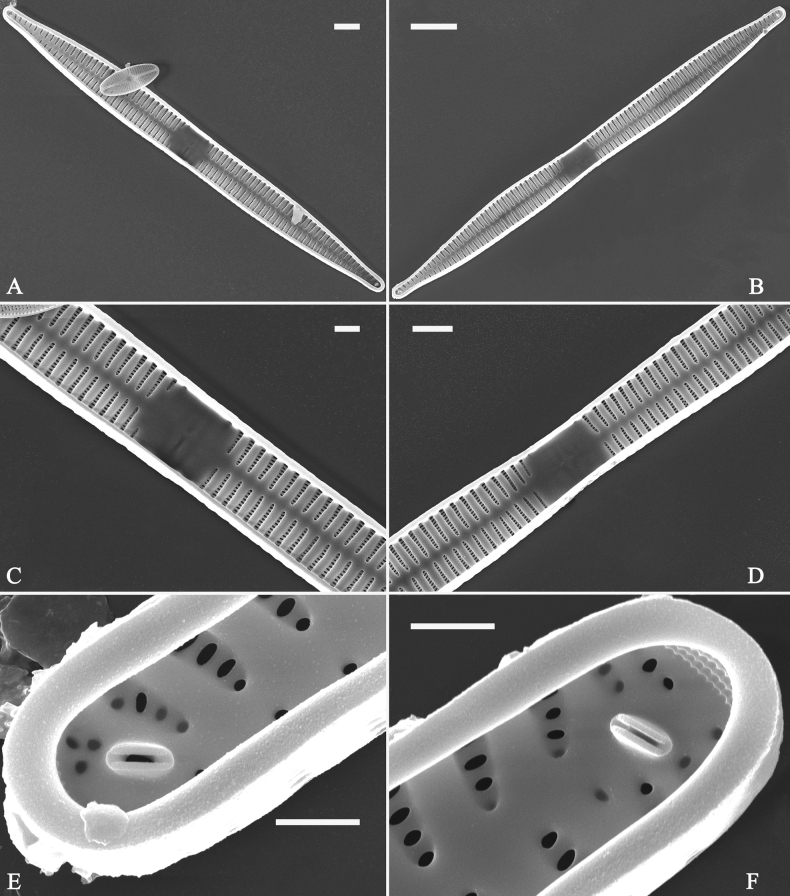
*Ulnariapandurata-uniseriata* sp. nov., internal view, SEM**A, B** two complete valves, note panduriform valve outline. **C, D** middle part details from **A**, **B**, respectively, note constricted central parts **E, F** two apical details from **B**. Scale bars: 10 μm (**A, B**); 1 μm (**C, D**); 2 μm (**E, F**).

**Figure 80.**
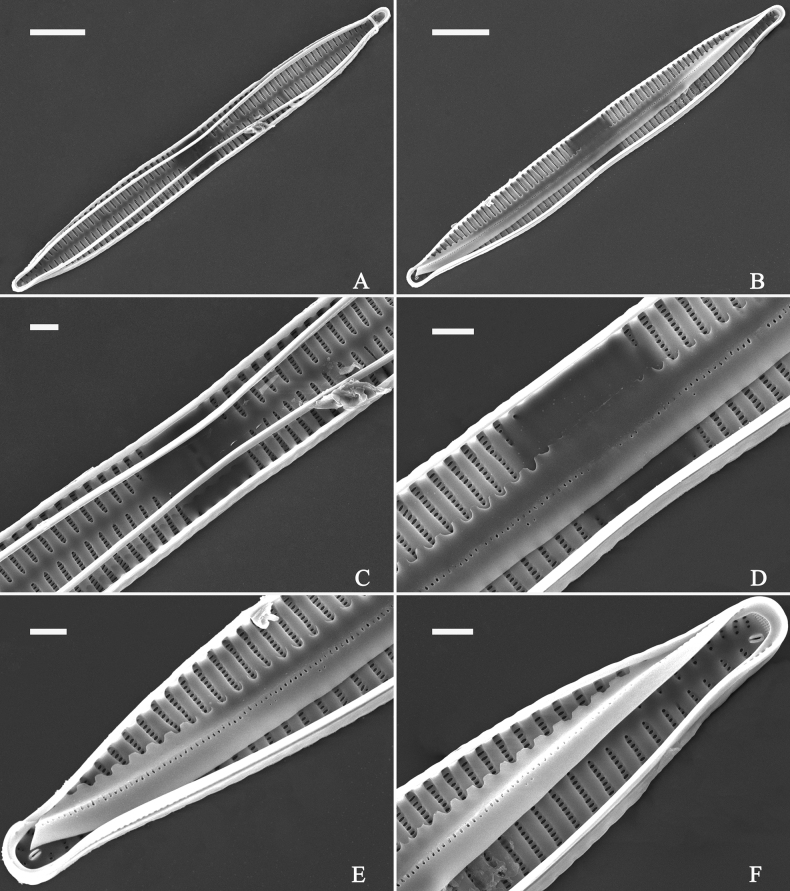
*Ulnariapandurata-uniseriata* sp. nov., internal view, SEM**A, B** two complete valves with valvocopula **C, D** two middle details from **A, B**, respectively **E, F** two apical details from **B**. Scale bars: 10 μm (**A, B**); 2 μm (**C–F**).

Figs 77 , 78 , 79 , 80

##### Holotype.

Slide JIUDIA202314, specimen circled on slide, illustrated as Fig. 77C.

##### Registration.

PhycoBank http://phycobank.org/103820.

##### Type locality.

China. Hunan province: Lanshan County, Shun River, a sampling location (25°11'29"N, 112°7'47"E, 490 m a.s.l.), collected by Bing Liu, October 5, 2021.

##### Description.

***LM*** (Figs 77). Valves panduriform with protracted, rostrate apices (Fig. 77A–J, see also Figs 78A, B, 79A, B, 80A, B). Valve dimensions (n = 28): length 70–116 μm, width 6.2–8.2 μm at centre. Sternum distinct, extending length of the valve. Central area distinct, rectangular fascia. Striae parallel, radiate only approaching each apex, and mostly opposite across sternum. Stria density 9–11 in 10 μm.

***SEM*** (Figs 78–80). Valve characterized by relatively wide virgae, interconnected with vimines (Fig. 78A–D). Ocellulimbus composed of ca. 17 pervalvar and 8 transverse rows of porelli. Two horn-like projections protruding over the ocellulimbus (Fig. 78E, F). Striae uniseriate, situated opposite each other across sternum, equidistant until radiate at poles, areolae become elongated closer to the mantle (Figs 78C, D, 79C, D). One rimoportula present at each pole, externally expressed as a simple hole (Fig. 78E, F), internally bilabiate, situated close to sternum (Figs 79E, F, 80E, F). Central area rectangular hyaline region (Figs 78C, D, 79C, D). Valvocopula a closed hoop, surrounding the valve internal margin (Fig. 80A, B), bearing a mostly continuous row of poroids dividing pars interior from pars exterior, located at the midline (Fig. 80B, D–F), lacking ornamentation at both poles (Fig. 80E, F). On its advalvar edge, valvocopula has a row of serrated projections, each corresponding internally to a virga (Fig. 80B, D–F).

##### Etymology.

The epithet *pandurata>-uniseriata* is a combination of the terms *pandurate* and *uniseriate* reflecting the panduriform outline of the valve and its mostly uniseriate striae.

##### Ecology and distribution.

Epilithic in a mountain stream with oligotrophic waters. The following environmental parameters were measured in the field: Conductivity was 53.9 ± 0.6 μS∙cm^-1^, pH was 8.4 ± 0.3 and water temperature was 21.9 ± 0.1 °C. So far, its distribution is known only from the type locality.

##### Discussion.

*Ulnariapandurata-uniseriata* is characterized by its panduriform valve outline, rectangular central area, mostly uniseriate striae, and its rostrate apices. It differs from *U.jumlensis* (Jüttner) D.M. Williams & Karthick by its narrower valve (6.2–8.2 μm vs 7.5–11.2 μm) and lower stria density (9–11 in 10 μm vs 13–14 in 10 μm) (see Jüttner et al. 2000, p. 249).

### ﻿Ultrastructure and configuration of girdle bands

**Figure 81.**
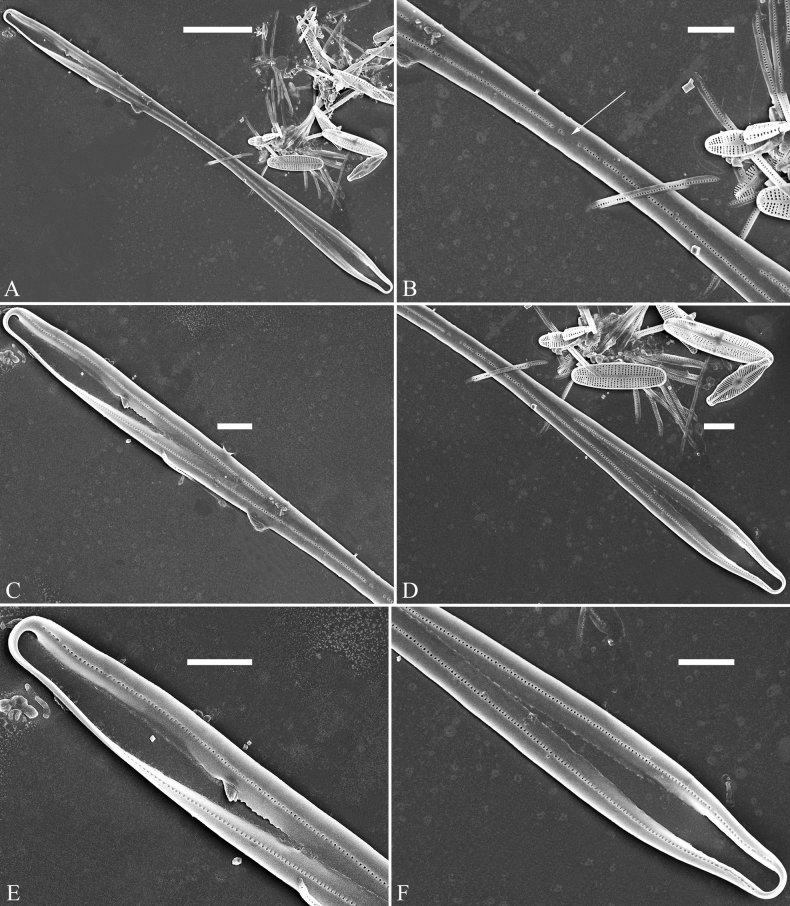
Valvocopula of *Ulnariaulnabiseriata*, SEM. Scale bar: 20 μm (**A**); 5 μm (**B–F**).

**Valvocopula ultrastructure**: In the above “Taxonomic treatment”, we illustrate a whole valvocopula that is separate from the valve in *U.neobiceps* sp. nov. (Fig. 53), which is a species possessing uniseriate striae. Here, we add an observation of a whole valvocopula from *U.ulnabiseriata* (Fig. 81), which is a species possessing mostly biseriate striae. Based on our observations all *Ulnaria* valvocopulae have similar ultrastructure: 1) the valvocopula is a closed hoop (Figs 53A, 81A); 2) usually, a row of poroids, which are interrupted in the middle (Figs 53B, 81B) and at two apices (Figs 53C–F, 81E, F), located at the midline, dividing pars interior from pars exterior; 3) the poroids of valvocopula are occluded by the closing plates same as those on valve surface (see Figs 43C, 47D, F); 4) on its advalvar edge (i.e., the edge of pars interior) except at its apical ends, valvocopula produces a row of serrated projections (which the other copulae do not produce) aligned with each virga (Fig. 81C–F); 5) at its apical end of the valvocopula, the advalvar edge forms a shelf which projects into the cell interior underneath the ocellulimbus (Figs 38F, 53C–F).

**Figure 82.**
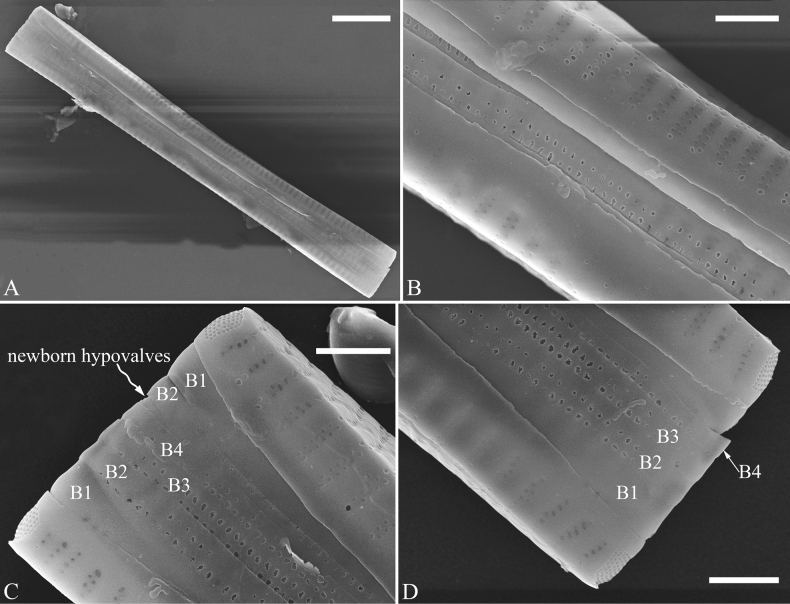
Configuration of girdle bands in *Ulnariaoxybiseriata*, SEM**A–D** four bands associated with the epivalve. Scale bars: 10 μm (**A**); 2 μm (**B–D**).

**Configuration of girdle bands**: The number of girdle bands associated with the hypovalve may vary, as can the number of girdle bands associated with the epivalve in the same species. Evidence is found in *U.oxybiseriata* D.M. Williams & Bing Liu and *U.jishou-biseriata* sp. nov. as the number of girdle bands associated with the epivalve can be either 3 (see Liu et al. 2019b, fig. 3C, E, F) or 4 (Fig. 82C, D, labelled B1 to B4) in *U.oxybiseriata* and *U.jishou-biseriata* (see Figs 21, 22). Thus, we interpret the configuration of girdle bands will change during the cell cycle. Some specific details are provided below and summarized in Table 5.

**Table 5.** Ultrastructure and configurations of girdle bands in 11 *Ulnaria* species.

Species	Mantle depth (μm)	Poroids in girdle bands	Configuration of girdle bands (dividing cells)	Configuration of girdle bands (non-dividing cells)
*U.constricta-biseriata* sp. nov.	2–4	Two rows of poroids produced in both 2^nd^ and 3^rd^ band, but the 2^nd^ row of poroids discontinuous	3:3	3:2
*U.dongtingensis*	2–3	One row of poroids in all girdle bands	No data (one ratio should be 3:3)	3:2
*U.fanjingensis* sp. nov.	2–3	One row of poroids in girdle bands except third copula which has second interrupted row of poroids	No data (one ratio should be 3:3)	3:2
*U.gaowangjiensis*	3–4	Second interrupted row of poroids in 4^th^ copula	4:4	No data
*U.hupingensis* sp. nov.	2–3.5	Second interrupted row of poroids in 2^nd^ copula	No data	Three girdle bands associated with the epivalve
*U.jinbianensis* sp. nov.	2–3	One row of poroids in all girdle bands	No data (one ratio should be 4:4)	4:2 at beginning
*U.jishou-biseriata* sp. nov.	2–3	Second interrupted row of poroids in 3^rd^ copula	No data	3:3 or 4:3
*U.oxybiseriata*	2–3	Second interrupted row of poroids in 3^rd^ copula	3:3	4:3
*U.sinensis*	2–4	One row of poroids in all girdle bands	No data (one ratio should be 4:4)	4:2
*U.sangzhi-biseriata* sp. nov.	2–3	Second interrupted row of poroids in 3^rd^ and 4^th^ copulae	No data (one ratio should be 4:4)	4:2
*U.ulnabiseriata*	2–3	One row of poroids in all girdle bands	4:4	4:2

**Figure 83.**
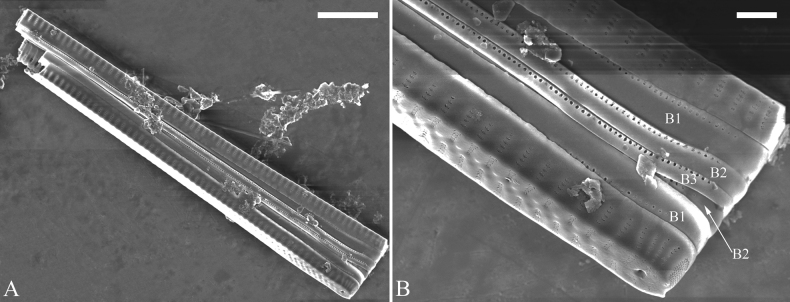
Configuration of girdle bands in *Ulnariadongtingensis*, SEM**A, B** a 3:2 configuration of girdle bands in non-dividing cells. Scale bars: 10 μm (**A**); 2 μm (**B**).

**Figure 84.**
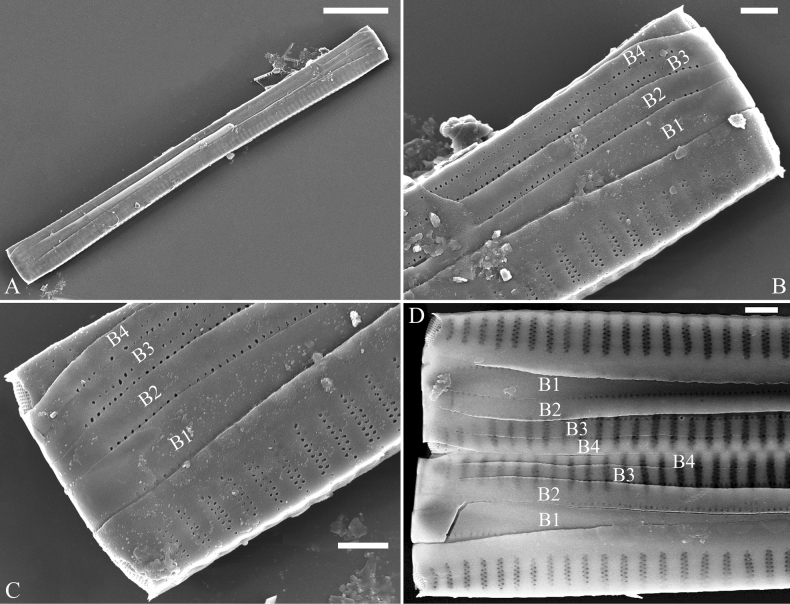
Configuration of girdle bands in *Ulnariagaowangjiensis*, SEM**A–C** four bands associated with the epivalve **D** A 4:4 configuration of girdle bands in dividing cells. Scale bars: 20 μm (**A**); 2 μm (**B–D**).

**Figure 85.**
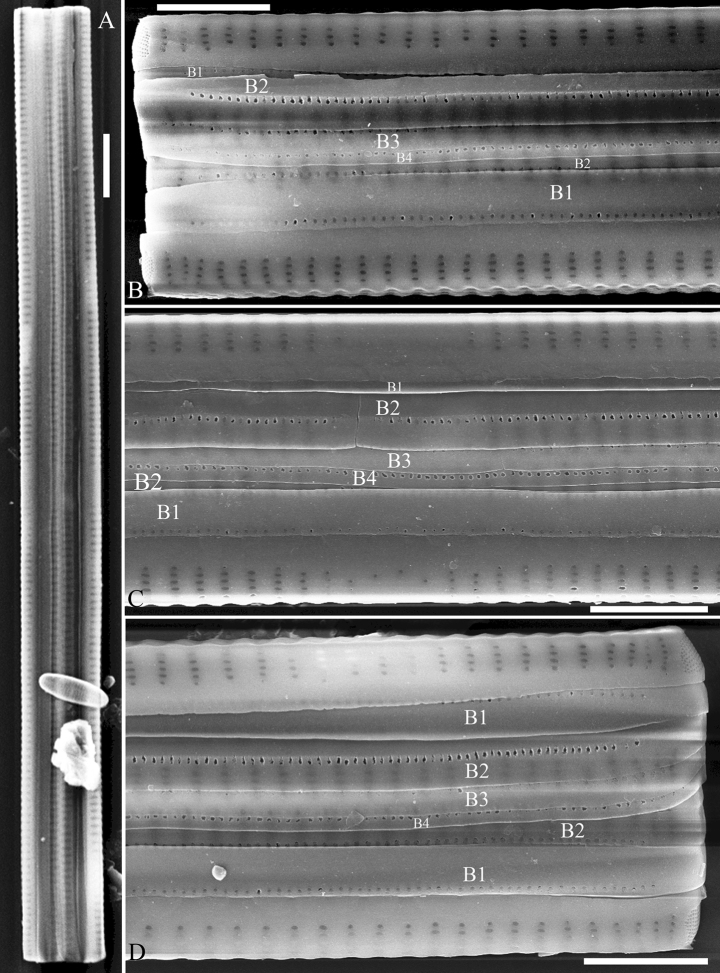
Configuration of girdle bands in *Ulnariajinbianensis*, SEM**A–D** a 4:2 configuration of girdle bands in non-dividing cells. Scale bars: 10 μm (**A**); 5 μm (**B–D**).

In *Ulnariaconstricta-biseriata* sp. nov., the epivalve is associated with a valvocopula and two copulae, so it has an overall configuration of 3:3 in dividing cells or 3:2 in non-dividing cells (Fig. 15). Two rows of poroids are present on both the second and third band; the second row of poroids is discontinuous (Fig. 15B–E). In the original description of *U.dongtingensis* (Liu et al. 2019b, p. 127), the girdle band configuration was not recorded. Further investigation revealed a 3:2 configuration of girdle bands in non-dividing cells (Fig. 83). In *U.fanjingensis* sp. nov., it has a 3:2 configuration in non-dividing cells (see Fig. 63B–D). In *U.gaowangjiensis* Bing Liu & D.M. Williams, its original description (Liu et al. 2017, p. 253) stated a 3:3 configuration in dividing cells but this is incorrect because further investigation revealed a 4:4 configuration in dividing cells (Fig. 84). In *U.hupingensis* sp. nov., three girdle bands are associated with the epivalve (see Fig. 69D). In *U.jishou-biseriata* sp. nov., it has a 3:3 or 4:3 configuration in dividing cells (see Figs 21, 22). In *U.oxybiseriata*, there is a 3:3 configuration in dividing cells (see Liu et al. 2019b, fig. 3C, E, F), but some non-dividing cells have a 4:3 configuration (Fig. 82). *Ulnariajinbianensis* S. Blanco & Bing Liu (Fig. 85), *U.sangzhi-biseriata* sp. nov. (Fig. 31), and *U.sinensis* Bing Liu et D.M. Williams (see Liu et al. 2017, figs 11–13), all have a 4:2 configuration of girdle bands in non-dividing cells. *Ulnariaulnabiseriata* has a 4:4 configuration in dividing cells (see Liu et al. 2017, figs 37–40).

Based on our published data (Liu et al. 2017, 2019a, b) and the above observations, the following conclusions can be drawn: 1) the valvocopula is different from the other copulae in ultrastructure; 2) the number of girdle bands associated with the epivalve is usually 3 or 4, but it varies among different *Ulnaria* species and even within the same species; 3) the configuration of girdle bands in dividing cells is either 4:4 or 3:3, i.e., the epivalve and hypovalve have the same number of girdle bands; 4) the deep mantle (often more than 2 μm) plus all girdle bands of a cell causes the cell depth to usually larger than the valve width so that the *Ulnaria* cells usually lie in girdle view on a slide.

## ﻿Discussion

### ﻿The life history of *Ulnaria*

There are rarely reports of the whole life history in the araphid diatoms, but a few papers mention the transverse perizonal bands of the initial cell. For example, Williams noted “no sign of transverse perizonal bands at all” in *Fragilariformavirescens* (Ralfs) D.M. Williams & Round (Williams 2001). Williams & Metzeltin noted that the auxospore/initial cells were rather large (more than 250 μm), curved along their length, with an irregular basal siliceous layer and the valve outline sometimes interrupted by undulations or a central inflation in a species of *Ulnaria* (Williams and Metzeltin 2004). Liu and Williams (2020) revealed the life history (except auxospore) of *Hannaeainaequidentata* (Lagerstedt) Genkal & Kharitonov and proposed that the life history of *H.inaequidentata* can be divided into the following four series of successive stages: auxospore, initial cell, pre-normal vegetative cell, and normal vegetative cell. There is a distinct pre-normal stage (i.e., from the initial cell finally to the normal cell) during which the life history must go through at least several generations (Liu and Williams 2020). Mann and Chepurnov observed the postauxospore shape modification of *Neidiumampliatum* (Ehrenberg) Krammer and found that its rostrate poles develop very quickly during the first divisions of the initial cell (Mann and Chepurnov 2005, p. 339) so that its pre-normal stage is very short. In *Ulnaria*, we found that *U.ulnabiseriata* has the same life history as *H.inaequidentata*. Although more investigations will be carried out in the future, a distinct pre-normal vegetative cell stage existing in both *Hannaea* and *Ulnaria* can be determined. The lack of transverse perizonium bands may be the cause of the chaos process – from irregular initial cells to irregular pre-normal vegetative cells – during its developing period from the initial cell to the normal vegetative cell (Liu and Williams 2020, p. 81).

### ﻿Living cells of *Ulnaria*

**Figure 86.**
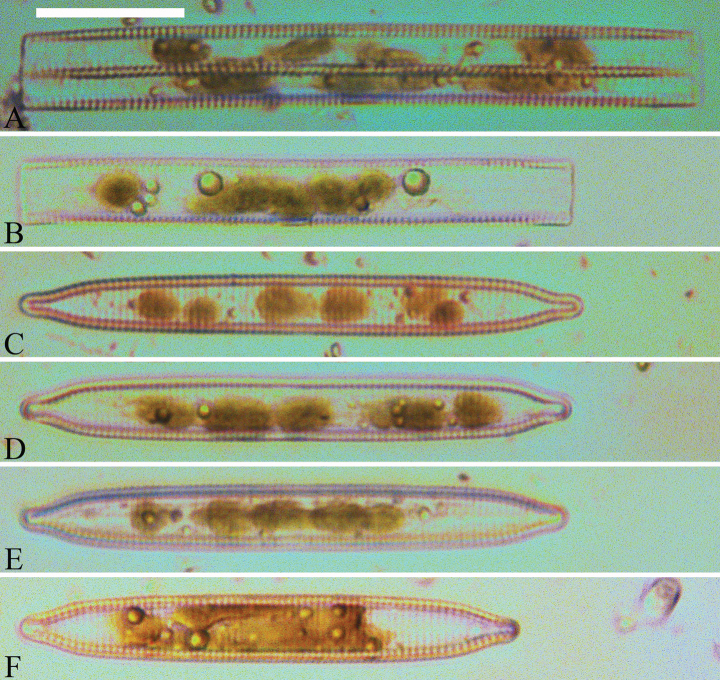
*Ulnariaoxybiseriata*, ×630, LM**A, B** living cells in girdle view, note numerous irregular chromatophores **C–F** four living cells in valve view, note numerous discoid chromatophores. Scale bar: 20 μm.

**Figure 87.**
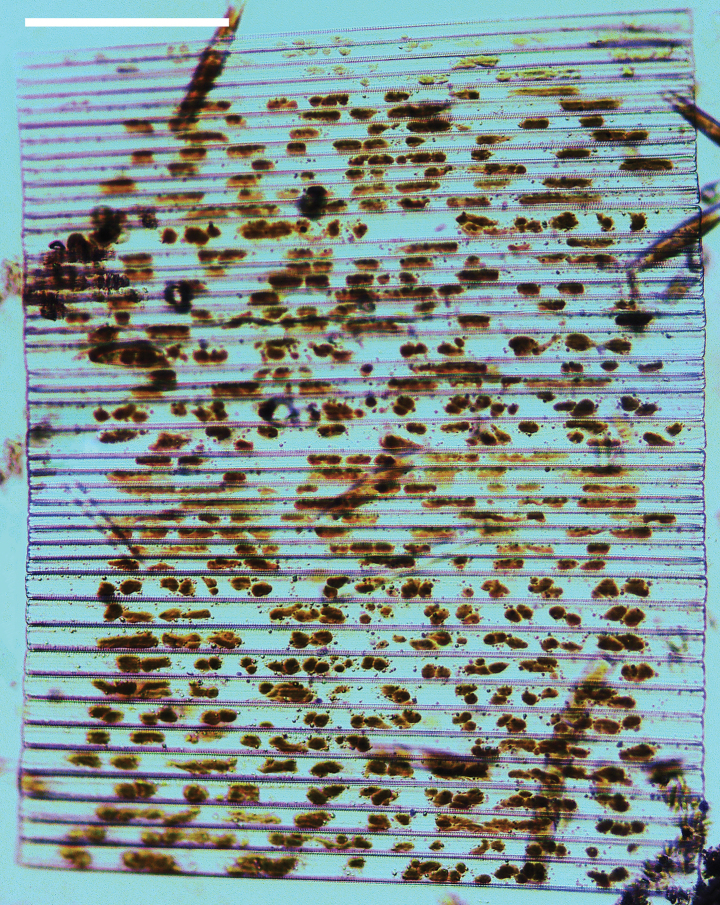
*Ulnariasinensis*, ×200, LM. A colony formed by more than 40 cells joined face-to-face, note numerous discoid chromatophores. Scale bar: 100 μm.

We observed living cells of five *Ulnaria* species (*U.fanjingensis* sp. nov., Fig. 61; *U.hupingensis* sp. nov., Fig. 67; *U.jishou-biseriata* sp. nov., Fig. 19; *U.oxybiseriata* Fig. 86; *U.sinensis*, Fig. 87). Only *U.sinensis* forms ribbon-like colonies (Fig. 87 showing a colony of *U.sinensis* composed of more than 40 cells joined face-to-face) whereas in the other four *Ulnaria* species are solitary. Round et al. (1990) pointed out that in *Synedra* (=*Ulnaria*) “Plastids: usually two long plates lying against the girdle and overlapping slightly on to the valve face. These may split up in unhealthy material, giving the impression of numerous discoid chromatophores”. Few authors discuss observations on the plastids of *Ulnaria* cells since Round et al. (1990). Morales et al. (2014) discussed living colonies of two *Ulnaria* species but did not mention their plastids. We observed that *U.jishou-biseriata* has two long valve-appressed chloroplasts per cell (Fig. 19). This agrees with the observation of Round et al. (1990). We also found that numerous discoid chromatophores are present in *U.fanjingensis* (Fig. 61), *U.hupingensis* (Fig. 67), *U.oxybiseriata* (Fig. 86), and *U.sinensis* (Fig. 87). The samples including these species were collected from the localities far away from our laboratory and their living cells were photographed after at least 24 hours of being collected, so their plastids may have changed from a long plate to numerous discs as the cells became unhealthy.

### ﻿Basal siliceous layer

**Figure 88.**
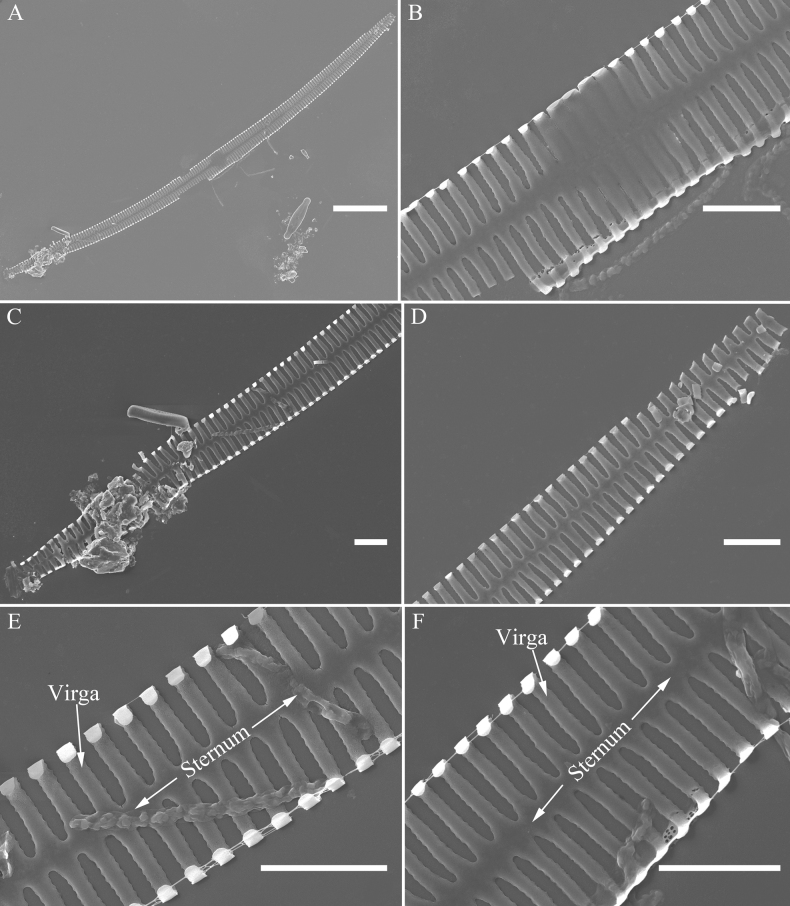
*Ulnariajishou-biseriata* sp. nov., internal view, SEM**A–F** an incompletely silicified valve showing valve basic structure composed of sternum and virgae. Scale bar: 20 μm (**A**); 4 μm (**B–F**).

**Figure 89.**
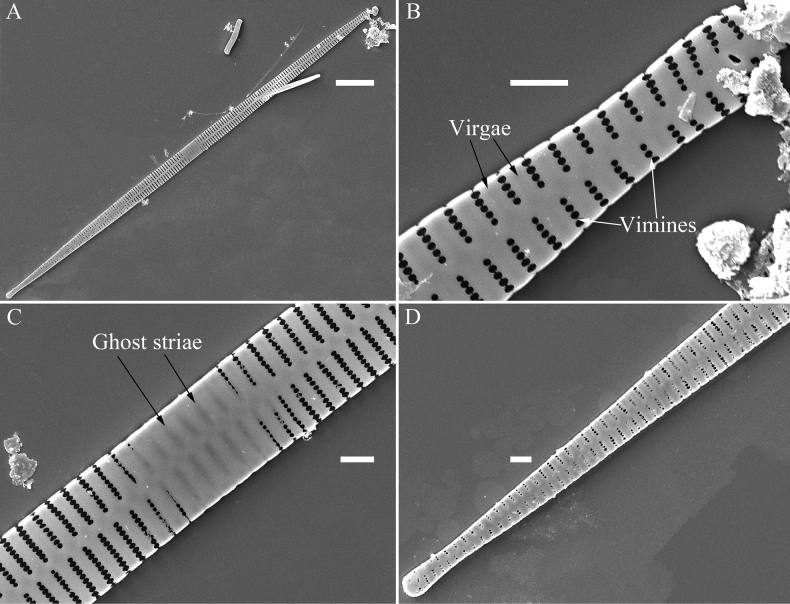
*Ulnariafanjingensis* sp. nov., external view, SEM**A–D** an incompletely silicified valve showing basic structure composed of sternum, vimines, and virgae, note some ghost striae in the central area. Scale bars: 20 μm (**A**); 2 μm (**B–D**).

**Figure 90.**
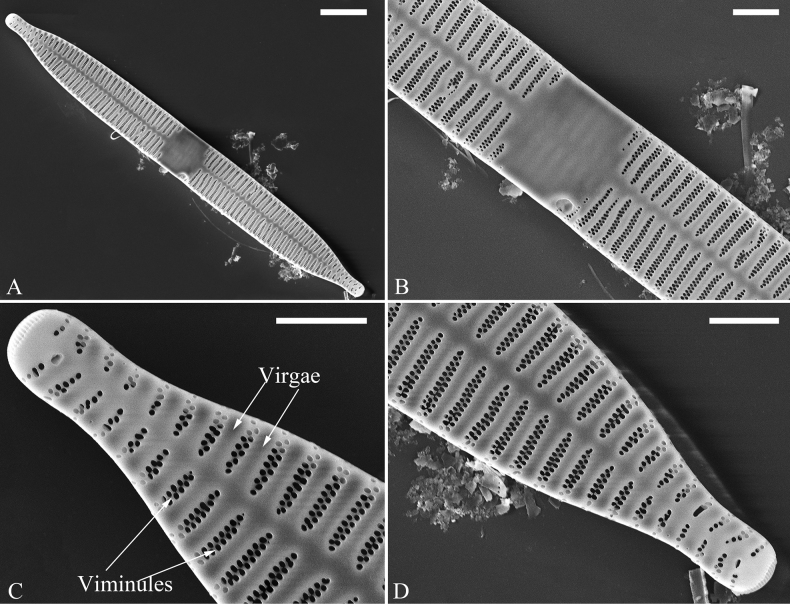
*Ulnariagaowangjiensis*, external view, SEM**A–D** an incompletely silicified valve showing basic structure composed of sternum, viminules, and virgae. Scale bars: 10 μm (**A**); 3 μm (**B–D**).

Ross et al. defined the basal siliceous layer as “the layer that forms the basic structure of the various components of the frustule” (Ross et al. 1979, p. 525). In *Ulnaria*, its basal siliceous layer composed of sternum, virgae, vimines/viminules, and striae. Researchers around the world have described many new *Ulnaria* species by using these terms (see Appendices 1–3 and references therein). However, some incompletely silicified valves can provide a good understanding of its basal siliceous layer even though I have illustrated and labelled most terms (see Figs 1, 2). Figure 88 shows the basal siliceous layer of *U.jishou-biseriata* sp. nov. This incompletely silicified valve is composed of sternum and virgae but the viminules were not developed, though the central area is already formed (Fig. 88). Figure 89 shows the basal siliceous layer of *U.fanjingensis* sp. nov. This incompletely silicified valve is composed of sternum, virgae, and vimines, and the central area formed and with ghost striae (Fig. 89). Figure 90 shows the basal siliceous layer of *U.gaowangjiensis*. This incompletely silicified valve is composed of sternum, virgae, and viminules, and the central area formed but the closing plates did not (Fig. 90). During the raphid diatom ontogeny, Round et al. (1990) revealed that the pattern of silica deposition as the raphid valve forms is that the primary side of the sternum forms first, then the secondary side of the sternum forms, and finally the formation of the valve. The ontogeny of *Ulnaria* is not well known. Based on our observations (Figs 88–90), the pattern of silica deposition in the valve formation is likely that the sternum forms first, next the virgae form, finally the formation of the valve.

**Figure 91.**
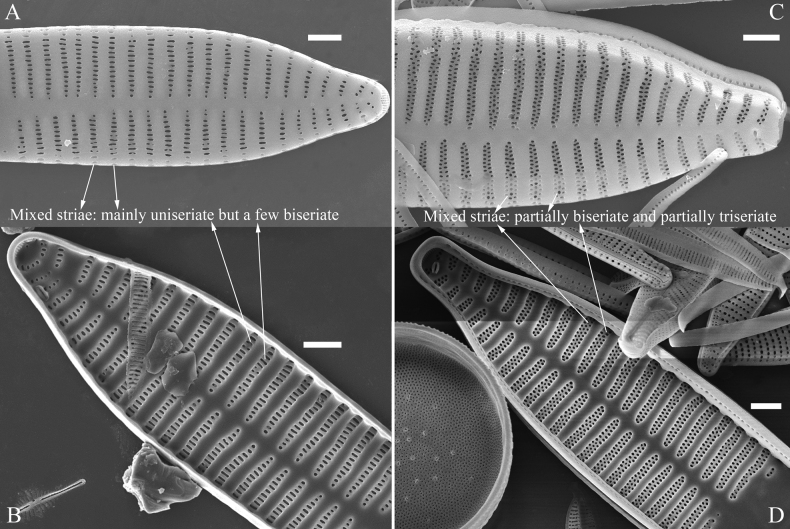
Mixed striae of *Ulnaria*, SEM**A, B** mixed striae in *U.jinbianensis***C, D** mixed striae in *U.pandurata-biseriata* sp. nov. Scale bars: 2 μm (**A–D**).

**Figure 92.**
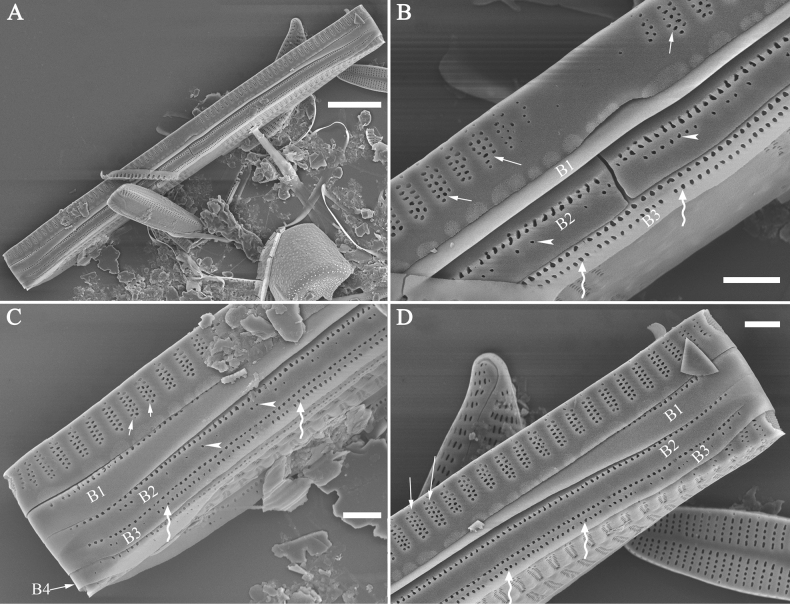
Mixed striae and poroids of girdle bands in *Ulnariagaowangjiensis*, SEM**A** a frustule **B–D** mixed striae in mantle (arrows), isolated poroids in second band (B2, arrowheads), and two rows of poroids in third band (B3, wavy arrows). Scale bars: 10 μm (**A**); 2 μm (**B–D**).

The striae are usually divided into three types: uniseriate, biseriate and multiseriate striae (Ross et al. 1979, Round et al. 1990). However, the situation is more complicated in *Ulnaria* because “mixed stria” may exist in some *Ulnaria* species. Following its definition (see above morphological terminology section), the mixed striae can be divided into three types at least. Figure 91 illustrates and labels these three types of mixed stria. The first type of mixed stria is one which is mostly uniseriate with small sections being biseriate, and an example of which is seen in *U.jinbianensis* (Fig. 91A, B). The second type of mixed stria is one which is the reverse being mostly biseriate with a small section (usually 1 to 2 areolae near sternum) of uniseriate. This type of mixed stria is the most common striae in “biseriate stria *Ulnaria*” (e.g., in *U.constricta-biseriata*, *U.jishou-biseriata*). The third type of mixed stria is one which is composed of a biseriate, a triseriate, and a small uniseriate (usually 1 to 2 areolae near sternum) sections, and example of which is seen in *U.pandurata-biseriata* (Fig. 91C, D). Thus, in fact, there are not whole biseriate striae in *Ulnaria* because the biseriate striae often become uniseriate – a very short part composed of usually 1 to 2 areolae – near sternum (e.g., in *U.constricta-biseriata*, *U.jishou-biseriata*). However, the uniseriate striae can be complete throughout the whole valve (e.g., in *U.menyuanensis*, *U.neobiceps*, *U.chengduoensis*, *U.qinghainensis*, *U.fanjingensis*, *U.hupingensis*, and *U.xieriverensis*). Additionally, in the distribution of striae on the valve from pole to pole, in the “biseriate stria *Ulnaria* species”, a few complete uniseriate striae maybe occur near each apex (see Figs 17, 24, 27, 33, 36). To date, no *Ulnaria* species have been found to possess mostly triseriate or quadraseriate striae. But a few quadraseriate striae are found in the mantle of *U.gaowangjiensis* (Fig. 92). The origin of the term *viminule* is obscure and although attributed to Cox (2012), it does not appear in that paper or any others but was used comprehensively by Morales et al. (2019). For the uniseriate striae there are only virgae and vimines but for the biseriate striae or multiseriate striae there are tiny ribs interconnecting each other to define the areolae. To distinguish the vimines in the uniseriate striae from the tiny ribs in the bi- or multiseriate striae, we formally define the viminules which have been used but not defined in Morales et al. (2019), Morales (2021), and Wetzel and Ector (2021).

**Figure 93.**
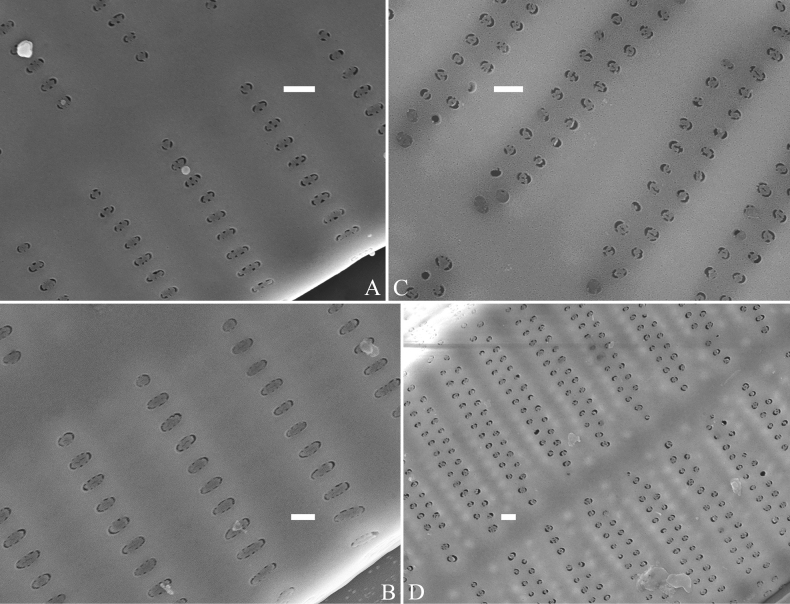
Closing plates of *Ulnaria*, external view, SEM**A***U.wulingensis***B***U.fanjingensis* sp. nov. **C***U.oxybiseriata***D***U.jishou-biseriata* sp. nov. Scale bars: 300 nm (**A, B, D**); 600 nm (**C**).

Closing plates occlude the external openings of areolae in *Ulnaria* (sensu Williams 1986). The structure of the closing plates in both uniseriate and mostly biseriate striae is similar, though the closing plates are different in size (Fig. 93). The closing plates are a plate that does not completely occlude the outside opening of areola. This plate is usually solid (sometimes with 1–3 perforations) and has a few struts affixed to each areolar wall. The mature closing plates lie flush with the valve surface. The closing plates in the uniseriate and mostly biseriate striae are illustrated and compared in Figure 93. The areola (pore) occlusions were defined in Ross and Sims (1972), Ross et al. (1979) and Mann (1981). Cox (2004) reassesses their structure and terminology and concludes the closing plate in *Ulnaria* cannot be attributed to a cribrum, hymen, rica, or rota, but it may be considered as a vola which is a catch-all term. To be precise, we use closing plate rather than vola.

### ﻿Central area, Ocellulimbus and Rimoportula

**Figure 94.**
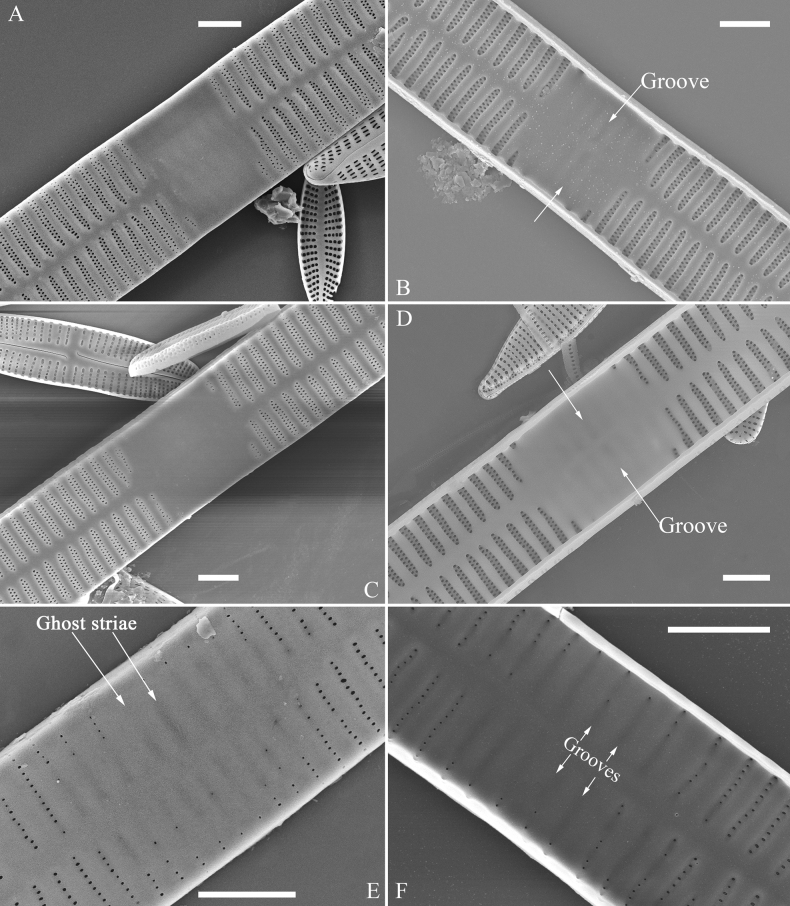
Central areas and ghost striae in *Ulnaria*, SEM**A, B***U.gaowangjiensis***C**, **D***U.ulnabiseriata***E**, **F***U.menyuanensis* sp. nov. Scale bars: 3 μm (**A–F**).

Ross et al. (1979) defined the central area as “an expanded or otherwise distinct portion of the axial area midway along its length”. The essence of this definition is that the central area is a hyaline area of no ornamentation. The central area is variable in *Ulnaria* taxa. Some species have a distinct central area (e.g., *U.gaowangjiensis* and *U.ulnabiseriata*, Fig. 94,), some have a very variable central area (e.g., *U.blancoi* and *U.neobiceps*), and some have distinctive ghost striae in the central area (e.g., *U.menyuanensis*, Figs 44, 94E, F). The ghost striae are formed by the unperforated grooves in the valve interior of the central area (sensu Lange-Bertalot and Ulrich 2014). *Ulnariamenyuanensis* has distinctive ghost striae in the central area just because in its valve central area there are remarkable internal unperforated grooves present (see Fig. 94E, F). Interestingly, ghost striae appear in the incompletely silicified valve of *U.fanjingensis* sp. nov. (Fig. 89) but do not appear in the mature valves (Fig. 62) because the internal grooves are fully silicified in the mature valves (see Fig. 66B, C). Most species described in this study have ghost striae in their central areas (except these species lacking a central area) because there are different levels of shallow grooves present. If the grooves are very shallow or not present, then no ghost striae are observed under LM.

**Figure 95.**
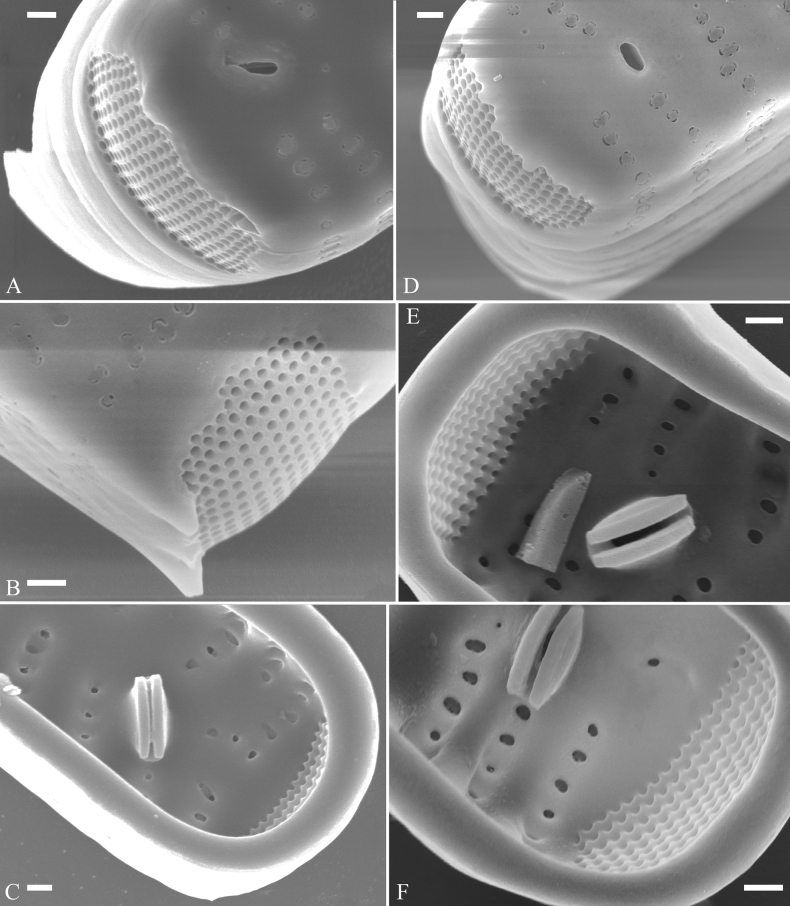
Ocellulimbus and rimoportula in *Ulnaria*, SEM**A–C***U.fanjingensis* sp. nov. (**A, B** external views **C** internal view) **D–F***U.jishou-biseriata* sp. nov. (**D** external view **E**, **F** internal views). Scale bars: 300 nm (**A–F**).

At first, the ocellulimbus was defined as a special type of pore field, a “plate set into the polar valve mantle” (Williams 1986, p. 146). In *Ulnaria*, ocellulimbus is composed of some pervalvar and transverse rows of porelli. Both pervalvar and transverse rows are unequal in length. The porelli are not occluded. The apical end of the valvocopula forms a shelf which projects into the cell interior underneath the ocellulimbus and it may block some porelli (see Figs 23D, E, 29C, D, 43E, F). In the normal vegetative cells, the inset ocellulimbus has pervalvar rows of porelli which are vertical (Fig. 95) whereas in the initial cell the ocellulibus extends onto the valve surface (Fig. 5F).

**Figure 96.**
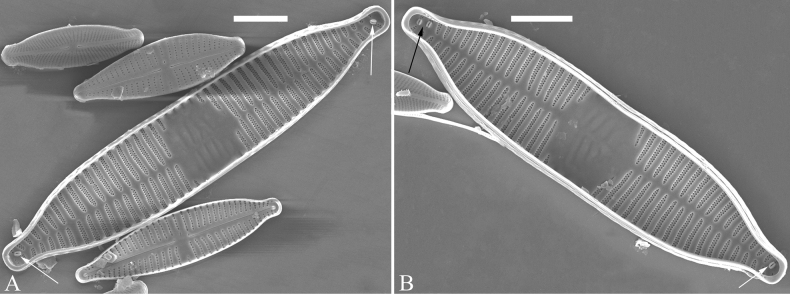
Rimoportulae in *Ulnariapandurata-biseriata* sp. nov., internal view, SEM. Scale bars: 6 μm (**A, B**).

**Figure 97.**
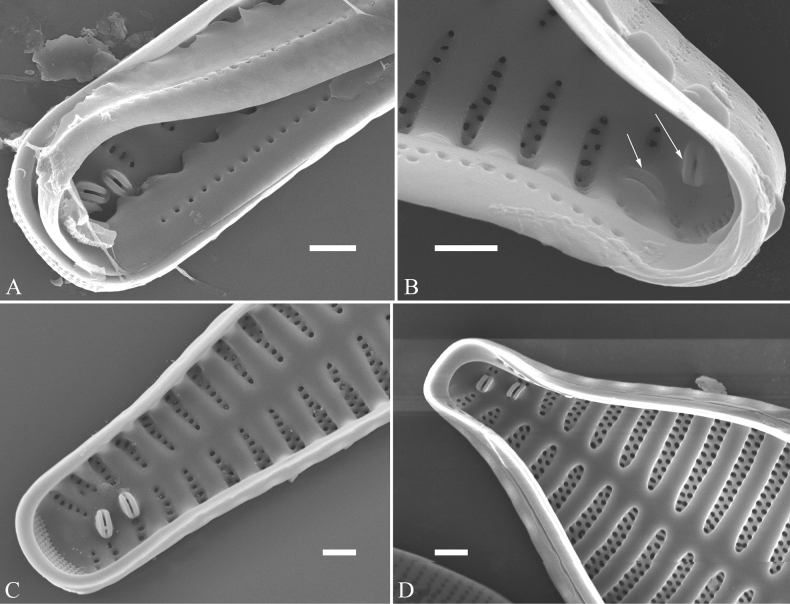
Rimoportulae in *Ulnaria*, internal view, SEM**A***U.hupingensis* sp. nov. **B***U.oxybiseriata***C***U.jishou-biseriata* sp. nov **D***U.pandurata-biseriata* sp. nov. Scale bars: 1 μm (**A–D**).

The rimoportulae in all *Ulnaria* have the same structure (more or less) – they are simple holes externally and bilabiate internally – with the number present and some aspects of the shape and position varying. One rimoportula is present at each apex in most *Ulnaria* species. However, in three *Ulnaria* species, *U.colcae* Van de Vijver & Cocquyt, *U.macilenta* E. Morales, C.E. Wetzel & S.F. Rivera, and *U.titicacaensis* E. Morales, Ector & P.B. Hamilton, only one rimoportula is present at one apex per valve (Van de Vijver and Cocquyt 2009, Morales et al. 2014). Interestingly, some *Ulnaria* species which usually have two rimoportulae (one at each apex) per valve, can also have three rimoportulae per valve (one at an apex and two at the other apex). Examples are in *U.pandurata-biseriata* sp. nov. (Fig. 96A, two rimoportulae per valve; Figs 96B, 97D, two rimoportulae at one apex, thus three rimoportulae per valve), *U.hupingensis* sp. nov. (Fig. 97A, two rimoportulae at one apex), *U.oxybiseriata* (Fig. 97B, two rimoportulae at one apex), and *U.jishou-biseriata* sp. nov. (Fig. 97C, two rimoportulae at one apex).

### ﻿Valvocopula ultrastructure and the configuration of girdle bands

In the 14 *Ulnaria* taxa described in this study, only in *U.chengduoensis* sp. nov. was the valvocopula not observed. We also document the whole valvocopula that is separate from the valve in *U.neobiceps* sp. nov. (Fig. 53) and *U.ulnabiseriata* (Fig. 81). Williams (2020, p. 5, figs 15–17) illustrated the valvocopula of *Ulnariavitrea* (Kützing) E. Reichardt. Later, Williams and Van de Vijver (2021, p. 173, figs 40–43) illustrated the valvocopula of *U.aequalis* (Kütz.) D.M. Williams et Van de Vijver. The valvocopula of the *Ulnaria* we describe here has a similar ultrastructure to those previously published.

In a diatom cell, the configuration of girdle bands is defined as the ratio between the number of girdle bands associated with the epivalve and those associated with the hypovalve (sensu Mann 1982). We may be the first to use this term in *Ulnaria*. Mann (1982, p. 173) pointed out that “There is a determinate number of bands in the mature cingulum, which again appears to be common condition in raphid diatoms”. The configuration of girdle bands can also be a common condition in araphid diatoms based our investigations. Other examples are *Ctenophorasinensis* Bing Liu & D.M. Williams, *Hannaeainaequidentata*, and *Diatomasinensis* Bing Liu & Rioual. There is a 4:2 configuration of girdle bands in the normal vegetative cells of both *C.sinensis* (Liu et al. 2020) and *D.sinensis* (Yuan et al. 2022). There is a 4:2 configuration of girdle bands in the normal but not dividing vegetative cells of *H.inaequidentata* whereas a 4:4 configuration appears in the dividing vegetative cells (Liu and Williams 2020). In 11 of the *Ulnaria* species investigated by us (Table 5), the number of girdle bands associated with the epivalve are either three or four and there are at least two girdle bands associated with the hypovalve. Thus, each cell has at least five girdle bands. The mantle depth is more than 2 μm (Table 5). Thus, the *Ulnaria* cell depth, which includes the depth of two mantle plus the depth of at least five overlapped girdle bands, is often larger than the width of the valve so that the *Ulnaria* cells often lie in girdle view on a slide. Published reports of the configuration of girdle bands in *Ulnaria* are rare. Only Zakharova et al. (2020, p. 42, fig. 3) stated, “tree (sic., should be three) closed girdle bands of each valve are pointed by arrowheads” which means there is a 3:3 configuration of girdle bands in *U.pilum* Kulikovskiy & Lange-Bertalot. The different methods of processing the diatom samples may be one of the reasons. The usual practice causes the diatom frustule to usually separate into two valves rather than to keep the frustule intact. Acquiring the specimens with intact frustules is the key to demonstrate the configuration of girdle bands. Based on our experience, if the diatom samples are heated gently for a shorter time, the intact frustules with all girdle bands can be easily found.

## ﻿Summary and future work

The life history of *Ulnaria* was revealed in this study for the first time. A distinct pre-normal vegetative cell stage exists during the process from the initial cells to the normal vegetative cells.

The configuration of girdle bands of 11 species and the valvocopula ultrastructure of 13 species are observed, illustrated, and described. A conclusion can be drawn at this moment that the configuration of girdle bands in *Ulnaria* cells is a common condition based on this study and our previous studies. The configuration of girdle bands changes during the cell cycle and the number of girdle bands in a cell will reach its maximum at the late stage of cell dividing. Thus, to prove all the girdle bands are closed, you must observe the cell at the late stage of cell division, which is difficult to achieve in practice. Herein, I recommend the closed valvocopula as a defining character for the genus *Ulnaria* because it is more feasible in practice and an equally effective alternative to “closed girdle bands”.

Most *Ulnaria* species possess uniseriate striae. Three types of mixed striae in *Ulnaria* are observed in this study. If we hypothesize that the first type of mixed striae is a transitional type from the uniseriate striae to mostly biseriate (the second type of mixed striae), and the third type of mixed striae is a transitional type from the mostly biseriate to the mostly triseriate striae, then we predict that the existence of *Ulnaria* species, which possesses mostly triseriate striae, is waiting to be found in nature.

## Supplementary Material

XML Treatment for Ulnaria constricta-biseriata

XML Treatment for Ulnaria jishou-biseriata

XML Treatment for Ulnaria pandurata-biseriata

XML Treatment for Ulnaria sangzhi-biseriata

XML Treatment for Ulnaria wuling-biseriata

XML Treatment for Ulnaria blancoi

XML Treatment for Ulnaria menyuanensis

XML Treatment for Ulnaria neobiceps

XML Treatment for Ulnaria chengduoensis

XML Treatment for Ulnaria qinghainensis

XML Treatment for Ulnaria fanjingensis

XML Treatment for Ulnaria hupingensis

XML Treatment for Ulnaria xieriverensis

XML Treatment for Ulnaria pandurata-uniseriata

## ﻿Additional information

### Author ORCIDs

Bing Liu https://orcid.org/0000-0002-8516-325X

### Data availability

All of the data that support the findings of this study are available in the main text or Supplementary Information.

## ﻿Appendix 1

**Table A1.** Seven *Ulnaria* taxa possessing both uniseriate striae and marginal spines.

No.	Taxon	Valve outline	Valve dimensions (μm): length (L), width (W)	Central area	Striae density (in 10 μm)	Nature of girdle bands	References
1	*U.cochabambina*	Linear-lanceolate with subrostrate or sub-capitate apices	L: 88–296; W: 8–9	Square or rectangle fascia	8–10	Valvocopula closed	Morales et al. 2009, Williams 2011
2	*U.fragilariaeformis*	Linear-lanceolate with rostrate or sub-capitate apices	L: 105–120; W: 3–5	Absent	No data	Closed	Williams 2020
3	*U.monodii*	Linear with with sub-capitate apices	L: 140–400; W: 7.5–8.5	Absent	8–9	Closed	Cantonati et al. 2018
4	*U.pseudogaillonii*	Linear with attenuate and rounded apices	L: 220–410; W: 8–10	Absent	7–9	Unknown	Kobayasi and Idei 1979, Kobayasi et al. 2006
5	*U.sinensis*	Linear with rostrate apices	L: 296–512; W: 6–8	Absent	8–9	Closed	Liu et al. 2017
6	*U.undulata*	Linear with rostrate to sub-capitate apices	No data	Absent	No data	Closed	Williams 2020
7	*U.ungeriana*	Linear-lanceolate with cuneately rostrate apices	L: 85–230; W: 8–10	Very variable	(7) 8–10	Unknown	Williams 1986, Compère 2001

## ﻿Appendix 2

**Table A2.** Fourteen *Ulnaria* taxa possessing biseriate striae.

No.	Taxon	Valve outline	Valve dimensions (μm): length (L), width (W)	Central area	Striae density (in 10 μm)	Nature of girdle bands	References
1	*U.acuscypriacus*	Linear-lanceolate with sub-capitate apices	L: 140–300; W: 6–6.8	Rectangular or square as incomplete fascia	8.5–10	At least two copulae closed	Cantonati et al. 2018
2	*U.constricta-biseriata* sp. nov.	Linear-lanceolate, constricted at centre, with rostrate to sub-capitate apices	L: 66–166; W: 5.5–8	Rectangular or square fascia	10–12	Closed	This paper
3	*U.gaowangjiensis*	Linear with rostrate apices	L: 61–108; W: 6.5–8.5	Square fascia	9–11	Closed	Liu et al. 2017
4	*U.goulardii*	Lanceolate with a distinctive constriction at the centre, apex cuneately rostrate	L: 55–90; W: 8–10	Rectangular or square fascia	9–11	Unknown	Williams 1986, Wetzel et al. 2022
5	*U.gusliakovii*	Linear with rostrate apices	L: 110–256; W: 4.5–6	Absent	10	Closed	Genkal et al. 2022
6	*U.jishou-biseriata* sp. nov.	Lanceolate with rostrate apices	L: 139–200; W: 6–8	Variable	10–12	Closed	This paper
7	*U.lanceolata*	Linear with cuneately rostrate apices	L: 55–75; W: 8–10	Rectangular or square fascia	8–10	Unknown	Williams 1986, Compère 2001
8	*U.nyansae*	Linear with sub-capitate to capitate apices	L: 55–175; W: 8–15	Half fascia	(12) 13–15	Unknown	Williams 1986, 2011
9	*U.oxybiseriata*	Linear-lanceolate to lanceolate with apiculate apices	L: 56–78; W: 6–9	Rectangular or trapezoid fascia	10–12	Valvocopula closed	Liu et al. 2019a
10	*U.pandurata-biseriata*	Panduriform with rostrate apices	L: 37–60; W: 7–9.5	Variable	9–11	Valvocopula closed	This paper
11	*U.sangzhi-biseriata* sp. nov.	Linear-lanceolate with capitate to sub-capitate apices	L: 49–91; W: 6.5–8.2	Rectangular or square fascia	10–12	Closed	This paper
12	*U.ulnabiseriata*	Linear-lanceolate with rostrate to sub-capitate apices	L: 105–229; W: 6–8	Rectangular fascia	9–11	Closed	Liu et al. 2017
13	U.ulnavar.spathulifera	Linear with spatulate apices	L: 134–190; W: 8–9	Circular to rectangular, sometimes flanked by short striae	9–10	Unknown	Morales et al. 2007, Aboal et al. 2003
14	*U.wuling-biseriata* sp. nov.	Linear with rostrate to sub-capitate apices	L: 160–200; W: 6.5–8	Rectangular or trapezoid fascia	10–11	Valvocopula closed	This paper

## ﻿Appendix 3

**Table A3.** Forty-two *Ulnaria* taxa possessing uniseriate striae but no marginal spines.

No.	Taxon	Valve outline	Valve dimensions (μm): length (L), width (W)	Central area	Striae density (in 10 μm)	Nature of girdle bands	References
1	*U.acus*	Lanceolate	L: 90–100; W: 4–6	Variable	12–15	Closed	Williams and Blanco 2019
2	*U.aequalis*	Linear	L: 50–200; W: 5–10	Variable	4–6	Closed	Williams and Van de Vijver 2021
3	*U.amphirhynchus*	Linear with rostrate apices	No data	No data	No data	Unknown	Ehrenberg 1843, Bukhtiyarova and Compère 2006
4	*U.biceps*	Lanceolate with capitate apices	No data	No data	No data	Unknown	Kützing 1844, Compère 2001
5	*U.blancoi* sp. nov.	Lanceolate with rostrate to sub-capitate apices	L: 104–236: W: 4.6–6.8	Very variable	10–13	Valvocopula closed	This paper
6	*U.capitata*	Linear with capitate, rhomboid apices	L: 255–406; W: 8	No clear central area	10–11	Unknown	Morales et al. 2007, Compère 2001
7	*U.chengduoensis* sp. nov.	Linear with rostrate apices	L: 42–66; W: 6–8	Not clearly visible due to presence of ghost striae or absent	12–15	Unknown	This paper
8	*U.colcae*	Linear to linear-lanceolate with subrostrate apices	L: 18–46; W: 3.9–5.2	Asymmetrical, rectangular to circular fascia	16–17	Closed	Van de Vijver and Cocquyt 2009
9	*U.contracta*	Constricted valve outline with rostrate to subrostrate apices	L: 84–140; W: 5–6	Rectangular of variable length	11–14	Unknown	Morales et al. 2007, Morales and Vis 2007
10	*U.danica*	Lanceolate with rostrate to sub-capitate apices	No data	No data	No data	Unknown	Kützing 1844, Bukhtiyarova and Compère 2006
11	*U.delicatissima*	Needle-shaped with sub-capitate apices	L: 142–330; W: 4.2–5	Variable	8.5–10	Unknown	Lange-Bertalot and Ulrich 2014
12	*U.dongtingensis*	Narrow-lanceolate with rostrate to capitate apices	L: 106–260; W: 5–7	Square fascia	10–12	Closed	Liu et al. 2019b
13	*U.fanjingensis* sp. nov.	Lanceolate with rostrate to sub-capitate apices	L: 165–291; W: 4.8–6.3	Apically rectangular fascia	9–12	Closed	This paper
14	*U.ferefusiformis*	Linear-lanceolate with sub-capitate apices 76–152, 4–4.8	L: 76–152; W: 4.0–4.8	Indistinctly defined	12–14	Unknown	Kulikovskiy et al. 2016
15	*U.grunowii*	Narrowly fusiform with sub-capitate apices	L: 100–380; W: 2.0–4.0	Not or not distinctly separated	11–15.5	Unknown	Lange-Bertalot and Ulrich 2014, Kusber et al. 2017
16	*U.hunanensis*	Linear-lanceolate with sub-capitate to rostrate apices	L: 62–166; W: 3.3–5.2	Variable	12–14	Unknown	Liu et al. 2019a, Williams and Blanco 2019
17	*U.hupingensis* sp. nov.	Lanceolate with rostrate to sub-capitate	L: 81–200; W: 5.4–7.0	Completely absent	9.5–11.5	Closed	This paper
18	*U.japonica*	Needle-shaped	No data	Present	11–14	Closed	Tuji and Williams 2007, Tuji 2009
19	*U.jinbianensis*	Linear-lanceolate with rostrate to cuneate apices	L: 42–91; W: 5.5–7.5	Rectangular to trapezoid fascia	10–11	Closed	Liu et al. 2019b
20	*U.jumlensis*	Slightly panduriform with apiculate apices	L: 37–48; W: 6.5–9.5 at centre	Present	13–14	Closed	Jüttner et al. 2000, Williams and Karthick 2021
21	*U.macilenta*	Linear-lanceolate with subrostrate to broadly rounded apices	L: 18.5–95; W: 2.5–3.5	Square to rectangular fascia	18–19	Unknown	Morales et al. 2014
22	*U.menyuanensis* sp. nov.	Lanceolate with cuneate to rostrate apices	L: 60–104; W: 5–7	Not clearly visible due to presence of ghost striae	12–14	Valvocopula closed	This paper
23	*U.neobiceps* sp. nov.	Linear-lanceolate with distinct capitate apices	L: 202–307; W: 4.5–6.7	Very variable	9–11	Valvocopula closed	This paper
24	*U.obtusa*	Linear with rounded apices	L: 150–200; W: 5–8	Absent	3–4	Closed	Williams and Van de Vijver 2021
25	*U.oxyrhynchus*	Linear with acute, conical to subrostrate apices	L: 43–64; W: 6–7	Present, flanked by short striae	13–14	Unknown	Morales et al. 2007, Aboal et al. 2003
26	*U.pandurata-uniseriata* sp. nov.	Panduriform with rostrate apices	L: 70–116; W: 6.2–8.2	Distinct, fascia-shaped	9–11	Valvocopula closed	This paper
27	*U.pilum*	Needle-shaped with sub-capitate apices	L: 218–295; W: 5.6–6.3	Rectangular	10–11.5	Unknown	Kulikovskiy et al. 2016
28	*U.qinghainensis* sp. nov.	Linear-lanceolate with sub-capitate apices	L: 88–223; W: 3.1–5.0	Completely absent	9–11	Valvocopula closed	This paper
29	*U.ramesii*	Linear-lanceolate with rostrate apices	L: 50–92; W: 8–9	Ellipsoid to rectangular	11–14	Closed	Morales et al. 2007, Ohtsuka et al. 2007
30	*U.repanda*	Undulate with broadly rounded apices	L: 380–425; W: 5–9	Absent	7–12	Unknown	You et al. 2008, Williams 2011
31	*U.rhombus*	Rhombic with sub-capitate apices	L: 90–184; W: 4–5.5	Rhomboid, circumscribed by shortened striae	13–14	Unknown	Liu et al. 2019a
32	*U.rostrata*	Linear-lanceolate with rostrate apices	No data	Rectangular	No data	Closed	Tuji and Williams 2007, Tuji 2009
33	*U.schroeteri*	Filiform	L: 300–450; W: 2.5–4	Rectangular	15–18	Closed	Lange-Bertalot and Ulrich 2014, Williams and Blanco 2019
34	*U.splendens*	Linear with rostrate apices	L: 60–300; W: 5–10	Round to oval hyaline portion	6–10	Closed	Williams and Van de Vijver 2021
35	*U.titicacaensis*	Lanceolate with subrostrate apices	L: 15.5–98.5; W: 4–6.5	Oval to square fascia, asymmetrical	16–18	Unknown	Morales et al. 2014
36	*U.tooelensis*	Linear to linear-elliptical with sub-capitate apices	L: 14–97.5; W: 3.5–5.5	Rectangular central area on one side	11–15	Closed	Graeff et al. 2013
37	*U.tortuos*	Arc	L: 120–450; W: 10–12	Present	5–10	Closed	Williams and Metzeltin 2004, Williams 2011
38	*U.ulna*	Needle-shaped with sub-capitate apices	L: 230–320; W: 6.3–7.7	Absent	9.2–10.4	Closed	Lange-Bertalot and Ulrich 2014
39	*U.verhaegeniana*	Linear with acute apices	L: 55–90; W: 5–6	Apically rectangular	10–12	Unknown	Van de Vijver et al. 2017
40	*U.vitrea*	Lanceolate	L: 90–120; W: 4.5–6	Present, variable	No data	Closed	Williams 2020
41	*U.wulingensis*	Narrowly linear-lanceolate	L: 153–233; W: 2.7–4.8	Rectangular fascia	12–14	Valvocopula closed	Liu et al. 2019a
42	*U.xieriverensis* sp. nov.	Linear with rostrate apices	L: 64–120; W: 6.0–8.6	Absent	10.5–12	Valvocopula closed	This paper
